# Association Between Physical Activity, Heart Rate Variability and Major Depressive Disorders: An Umbrella Review

**DOI:** 10.1007/s40279-025-02365-5

**Published:** 2026-02-04

**Authors:** Jan-Niklas Kreppke, Gavin Brupbacher, Kerstin Rupf, Vivien Hohberg, Robyn Cody, Markus Gerber, Oliver Faude

**Affiliations:** 1 Department of Sport, Exercise and Health, University of Basel, Grosse Allee 6, 4052 Basel, Switzerland; 2 Clinic Oberwaid AG, 9016 St. Gallen, Switzerland; 3 Department of Consultation-Liaison Psychiatry and Psychosomatic Medicine, University Hospital Zurich, 8091 Zurich, Switzerland; 4 Institute for Implementation Science in Health Care, University of Zurich, 8006 Zurich, Switzerland

## Abstract

**Background:**

Physical activity (PA) has positive effects on physical and mental health. Nevertheless, two thirds of individuals with major depressive disorders (MDD) do not meet PA recommendations. Furthermore, MDD is associated with increased cardiovascular risk and autonomic nervous system dysfunction. This depression-related dysfunction is expressed by low heart rate variability (HRV). In contrast, regular PA has been shown to improve autonomic regulation over time as indicated by higher HRV values.

**Objective:**

The overarching aim of this umbrella review is to examine the complex interrelationship and mutual influence between MDD, PA, and HRV. We aim to answer the following questions: (I) How does PA affect depressive symptoms? (II) How are HRV and MDD related? (III) How does PA affect HRV?

**Methods:**

Medline, Embase, PsycINFO, SPORTDiscus, and Epistemonikos were searched for systematic reviews and meta-analyses from their inception until 5 July 2024. Reviews were included if they presented the effects of PA interventions on depression severity in individuals with MDD for part I, if they showed the differences in HRV parameters between healthy individuals and individuals with MDD for part II, and if they presented the effects of PA interventions on HRV parameters in healthy individuals for part III. The quality of the included reviews was assessed by Assessing the Methodological Quality of Systematic Reviews (AMSTAR 2) criteria.

**Results:**

This umbrella review included 90 reviews. Part I shows that various types of PA are effective in reducing symptoms of depression in individuals with MDD. In addition, there is a similar reduction with PA compared with pharmacological treatment. Part II demonstrates that time-based and frequency-based HRV values are lower in individuals with MDD than in healthy individuals. Part III shows that different types of PA can improve time-based, frequency-based and nonlinear HRV parameters in healthy individuals.

**Conclusion:**

This comprehensive umbrella review demonstrates the importance of PA to reduce symptoms of depression and to have a positive impact on autonomic function. Further studies suggest that PA can also improve HRV in individuals with MDD. In the future, randomized controlled trials should be conducted to examine which types and forms of PA are effective in positively affecting HRV in individuals with MDD according to standardized parameters in the area of HRV and depression severity.

**Trial Registration:**

The protocol for this umbrella review was a priori registered with PROSPERO: CRD42021278665.

**Supplementary Information:**

The online version contains supplementary material available at https://doi.org/10.1007/s40279-025-02365-5.

## Key Points

**Table:** 

Physical activity effectively reduces depression severity in individuals with major depressive disorder (MDD) and can improve heart rate variability (HRV), especially in healthy individuals.
Individuals with MDD have lower HRV than healthy individuals, indicating autonomic dysfunction. First analyses show that regular physical activity may improve HRV parameters in individuals with MDD.
There are a large number of systematic reviews and meta-analyses in the field, especially on the effects of physical activity on depression severity in individuals with MDD.

## Introduction

Physical activity (PA) is recommended in health guidelines and implemented in many therapies as an adjunct or standard therapy for individuals with mental illness [1, 2], because of positive effects on physical and mental health [3, 4]. Despite the knowledge of the benefits of PA, 28% of adults worldwide are insufficiently physically active [5]. This circumstance is even more pronounced in individuals with major depressive disorder (MDD). Individuals with MDD are less physically active than healthy controls and approximately 67% do not meet the PA recommendations [6]. Individuals with MDD are less likely to engage in regular PA, exercise and sport for several reasons, including comorbidities, increased fatigue, psychosocial stress, lack of motivation, lack of self-efficacy, and negative outcome expectations [7]. It is estimated that around 280 million individuals have MDD, making it the most common mental health disorder worldwide [8]. Lifetime prevalence ranges from 10 to 20% and it affects individuals of all ages, sexes/genders and socioeconomic status [9, 10].

Evidence supports a causal relationship between (lack of) PA and MDD [6, 11, 12]. PA has been shown to have a preventive effect on MDD [12–14]. People who meet WHO recommendations for PA have a 25% lower risk of developing depression than less physically active counterparts [13]. If individuals have MDD, PA is effective in reducing symptom severity [11]. There is also evidence that PA is as effective in these respects as pharmacological treatment or psychotherapy [1]. PA is traditionally defined as “any bodily movement produced by skeletal muscles that requires energy expenditure” and can be performed during leisure time or in an occupational context [15]. In this review, PA is used as an umbrella term for physical activity, sport and exercise. Sport is typically practiced for performance and competition or for the experience and can be seen as a form of PA [16, 17]. Exercise is typically planned or structured and requires repetitive bodily movements with the aim of maintaining or improving health through physical fitness, well-being, and social interaction [15]. It has been shown that different types of PA are effective in reducing depressive severity, such as aerobic and resistance training, walking or yoga [18]. PA also has a positive effect on depression remission odds, as seen in those who meet PA recommendations and over the course of a year have a lower risk of recurrent depression [19].

MDD is also associated with increased cardiovascular risk [20] and autonomic nervous system (ANS) dysfunction [21, 22]. The association between MDD and ANS dysfunction is potentially bidirectional. It is not clear whether depression symptoms lead to ANS dysfunction or whether ANS dysfunction increases the risk of depression [21, 23–25]. The exact pathogenesis in individuals with MDD remains unknown. The imbalance of the ANS in individuals with MDD is reflected by a sustained activation of the sympathetic nervous system without the normal counteraction of the parasympathetic nervous system [26, 27]. This depression-associated ANS dysfunction is expressed by elevated heart rate and low heart rate variability (HRV), among others [21, 28].

HRV characterizes the variability of the intervals between single heartbeats and is often used to determine ANS function. HRV can be measured using time and frequency domains, and nonlinear metrics, which are supposed to indicate parasympathetic and sympathetic nervous system activity [24, 29]. HRV is largely influenced by parasympathetic ANS activity which downregulates heart rate via acetylcholine release from the vagus nerve, and sympathetic ANS activity, which accelerates the heart via noradrenergic nerves [30]. Depending on the time and frequency domains, different interpretations are possible. Lower time-based parameters may indicate lower overall variability of the heart rate, point to lower parasympathetic and vagal activity, and indicate poorer overall autonomic activity, which is regulated by the parasympathetic and sympathetic activity. Lower frequency-based parameters may indicate lower parasympathetic and vagal activity and poorer activity of the parasympathetic and sympathetic systems [21, 31, 32].

Regular PA has been shown to improve autonomic regulation over time, as indicated by increased HRV due to higher parasympathetic activity and lower resting heart rate [33, 34]. PA can generally positively affect ANS activity, for example, by increasing parasympathetic activity [35]. Higher HRV values at rest are associated with better emotional regulation and mental state [36], and indicate that the ANS can adapt quickly to unexpected changes [37]. However, HRV adaptations are individual and depend on type, form and intensity of PA, recovery capacity, environment and other factors [38–40].

Current evidence suggests that PA, MDD and HRV are interrelated and may affect each other. Although various interactions have been reviewed individually, a simultaneous systematic review (SR) of all three domains is still missing. Currently, there are only a few studies that look at the effects of PA on HRV in individuals with MDD. Therefore, it is not yet possible to determine whether HRV in individuals with MDD can be positively affected by PA and which types of PA are effective [23, 41, 42]. Furthermore, umbrella reviews on the influence of PA on depression severity exist [43–45], but they do not specifically include adult individuals with MDD. To the best of the authors’ knowledge, there are meta-analyses (MAs) showing how PA affects HRV in healthy individuals and other clinical populations [35, 46], but not in individuals with MDD.

**Fig. 1:**
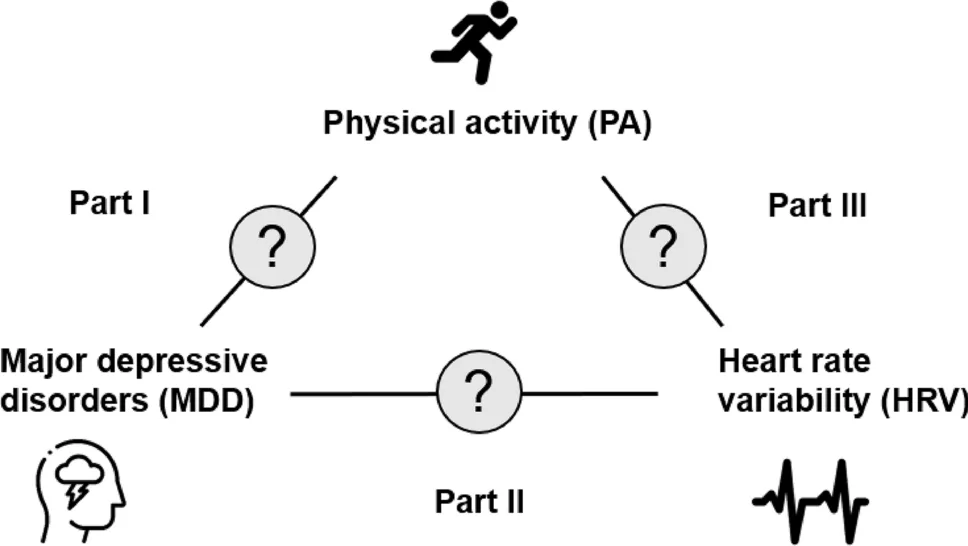
How do physical activity, major depressive disorder and heart rate variability affect each other? The three parts of this umbrella review

The various interactions of the three domains may also be important for understanding how the ANS is affected by PA and MDD. We conducted this umbrella review because the interrelationship between the three domains is still unclear (Fig. 1). Therefore, the overarching aim of this umbrella review is to examine the complex interrelationship and mutual influence between MDD, PA and HRV. In this regard, we provide answers to the following questions: (I) How does PA affect depression severity? (II) How are HRV and MDD related? (III) How does PA affect HRV?

## Methods

### Registration and Guidelines

We conducted an umbrella review of systematic reviews and meta-analyses on the interrelations between PA, HRV, and MDD. The review was registered a priori in PROSPERO (International Prospective Register of Systematic Review; registration number CRD42021278665) and was designed according to the PRISMA (Preferred Reporting Items for Systematic Reviews and Meta-Analyses) guidelines (Supplement 1 in the electronic supplementary material [ESM]) as well as the recommendations for umbrella reviews [47, 48].

### Literature Search

The systematic research was conducted in five databases: Medline (on Ovid), Embase (on Ovid), PsycINFO (on Ovid), SPORTDiscus (on EBSCO), and Epistemonikos (on epistemonikos.org) from their inception until 05 July 2024. The search strategy was examined by an information scientist from Basel Medical University Library using the Peer Review of Electronic Search Strategies (PRESS) guidelines [49]. Based on the PRESS guidelines, two researchers (JNK and KR) carried out the search independently. Because only SRs and MAs were included, reference lists, grey literature, and study registries were not searched and expert opinions were not consulted. The following terms were used: (depression AND physical activity) OR (depression AND heart rate variability) OR (physical activity AND heart rate variability). Synonyms were used for all the terms. The search strings were adapted to the databases (Supplement 2 in the ESM). Furthermore, specific filters were used for SRs and MAs and for adults.

### Review Selection

Literature was imported in the reference management software EndNote® X8 (Thomson Reuters, New York, NY, USA). Deduplication was performed in a first step using the Systematic Review Assistant—Deduplication Module for a high sensitivity and specificity [50]. In a second step, further duplicates were identified using Endnote and the online systematic review software Covidence [51].

**Fig. 2:**
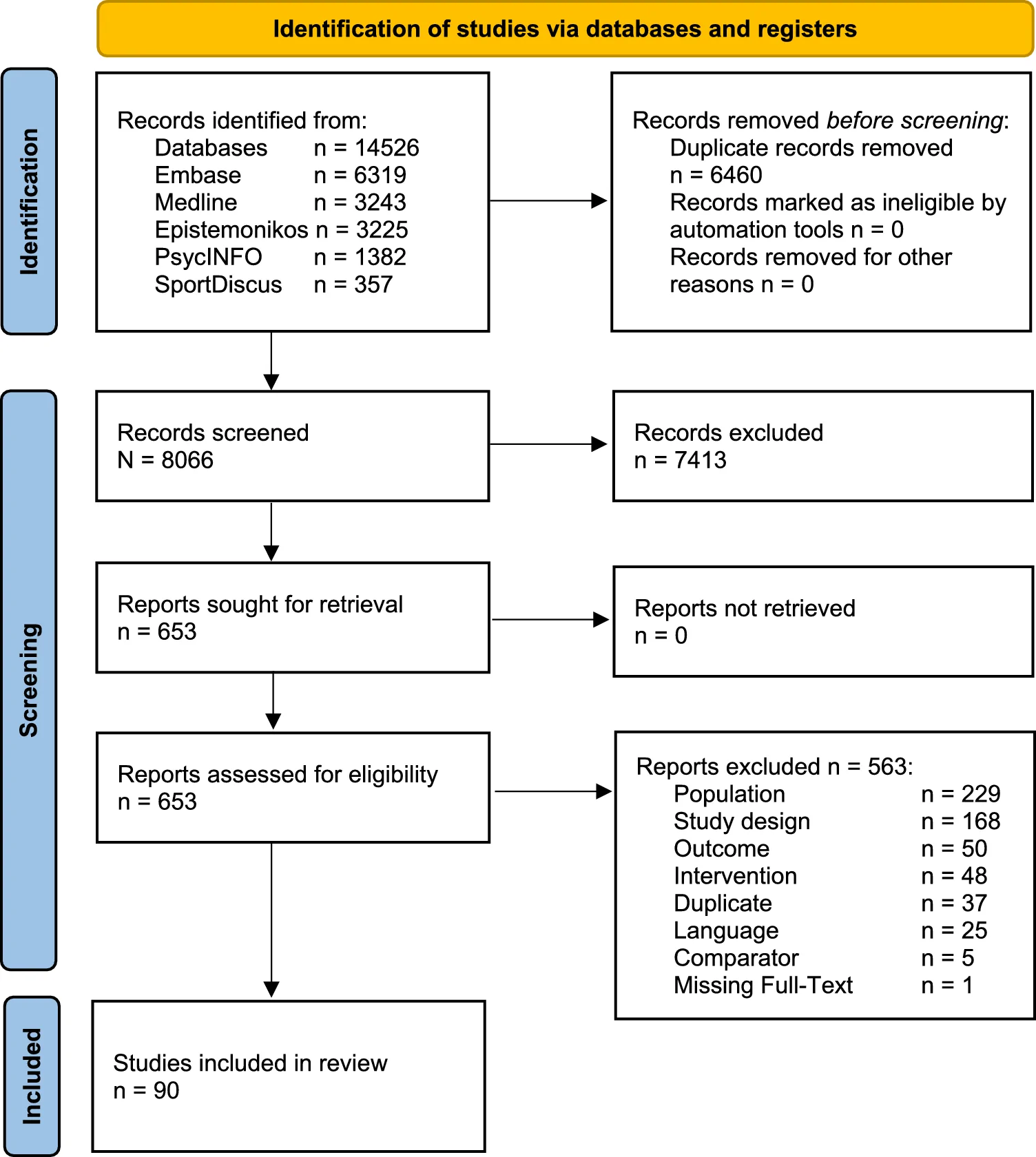
PRISMA flowchart

After deduplication, two reviewers (JNK and KR) independently screened titles and abstracts. Full-text screening based on inclusion and exclusion criteria was conducted by the same reviewers. For the screening process, that is, to judge eligibility and resolve issues, the online systematic review software Covidence [51] was used. Disagreements were discussed between the two reviewers. If disagreement remained, a third reviewer (OF) was consulted. Inter-rater agreement was reported using raw agreement in percent and Cohen’s kappa [52]. A flow diagram according to the PRISMA guidelines illustrates the number of included and excluded studies [48] (Fig. 2). The exclusion reason for every full text is listed in Supplement 3 (see ESM).

### Eligibility Criteria

The inclusion and exclusion criteria were set with the PICOS scheme (population, intervention, comparator, outcome, study design). The inclusion and exclusion criteria for the three parts of this umbrella review are presented in Table 1. We included only SRs or SRs with MA. In addition, cut-off values for eligibility were formed for the population criterion. For this purpose, in parts I, II, and III, an a priori analysis was made of the age distribution in possible reviews to be included. The authors decided that in the included SRs and MAs at least 75% of the included studies must adhere to the correct age (> 18 years), or results on this population must be reported separately. The same applies to the inclusion of individuals with MDD in part I and II. Here, SRs and MAs were included in which at least 75% of the included studies reported on individuals primarily diagnosed with MDD or this population was reported separately in the results.

**Table 1:** Inclusion and exclusion criteria for studies

		Inclusion criteria	Exclusion criteria
*Physical activity and major depressive disorder*			
P	Population	Individuals 18 years of age and older, with a primary diagnosis (ICD-10 or DSM-IV) of depression (Major Depressive Disorder) in inpatient or outpatient treatment	Younger than 18 years of age, depression is not the primary diagnosis (Major Depressive Disorder) or other mental illness
I	Intervention	Physical activity interventions; aerobic exercise, anaerobic exercise, resistance training, neuromuscular training, mind-body exercise	Interventions without physical activity
C	Comparison	Control or comparison group with no, different, or placebo treatment	
O	Outcome	Depression symptoms and/or severity (HAMD, BDI-II, HoNOS)	Depression symptoms or severity not recorded
S	Study type	Systematic reviews and meta-analyses	Other study type than systematic review or meta-analyses
*Heart rate variability and major depressive disorder*			
P	Population	Individuals 18 years of age and older, with a primary diagnosis (ICD-10 or DSM-IV) of depression (Major Depressive Disorder) and inpatient and/or outpatient treatment vs. healthy individuals 18 years of age and older	Younger than 18 years of age, depression is not the primary diagnosis (Major Depressive Disorder) or other mental illness
I	Intervention	No intervention	
C	Comparison	Comparison between healthy individuals and individuals with depression	No comparison of HRV between healthy individuals and individuals with depression
O	Outcome	Autonomic function measured by values of HRV	HRV values not recorded
S	Study type	Systematic reviews and meta-analyses	Other study type than systematic reviews or meta-analyses
*Physical activity and heart rate variability*			
P	Population	Healthy individuals 18 years of age and older	Individuals younger than 18 years
I	Intervention	Physical activity interventions; aerobic exercise, anaerobic exercise, resistance training, neuromuscular training, mind-body exercise	Interventions without physical activity
C	Comparison	Control or comparison group with no intervention	
O	Outcome	Autonomic function measured by HRV	HRV values not recorded
S	Study type	Systematic reviews and meta-analyses	Other study type than systematic reviews or meta-analyses

*BDI-II* Beck Depression Inventory, second edition, *DSM-IV* Diagnostic and statistical manual of mental disorders, fourth edition, *HAMD* Hamilton depression rating scale, *HoNOS* Health of the Nation Outcome Scales, *HRV* Heart rate variability, *ICD-10* International classification of diseases, 10th revision

### Quality Assessment

The quality of the included reviews was assessed by Assessing the Methodological Quality of Systematic Reviews (AMSTAR 2) criteria [53]. The quality assessment for the included literature on part I was conducted independently by two reviewers (JNK and KR). Further literature quality assessment for parts II and III was carried out by reviewer JNK. The overall quality using AMSTAR 2 was rated as being high, moderate, low, or critically low (Supplement 4 in the ESM).

### Data Extraction

Data were extracted in an a priori defined Excel® form. If important data or documents were missing from the included studies, we contacted the authors directly. The literature for part I was extracted independently by two reviewers (JNK and KR). Because the two reviewers showed > 80% agreement in the extraction, reviewer JNK extracted the literature for parts II and III [53]. The extracted data are listed in Supplements 5–7 of the ESM and included the following categories: reference with authors and publication year, characteristics with study type and number of included studies and individuals, objective, outcomes, type of PA, conclusion, and the quality rating by AMSTAR 2. When data were extracted from SRs with MA, the effect sizes as standardized mean differences (Cohen’s d, Hedges’ g) or unstandardized mean differences (MD) with 95% confidence intervals (CIs) were also reported. If reviews in part I compared the intervention against treatment as usual or no PA, this was reported as the control in Sect. 3. From the reviews, only results that were relevant to our research questions were extracted. It may therefore be that several results were extracted from one review.

### Analysis

We conducted a comprehensive quality assessment and descriptive overview of the included reviews. We present the results qualitatively from the SRs and MAs and the effect sizes quantitatively from the MAs.

In part I, effect sizes for changes in depression symptoms, depression severity, and treatment response rate were extracted from the meta-analyses. Effect sizes are presented as negative values if they indicate a reduction in depression severity and, thus, a beneficial effect.

In part II, the effect sizes of differences in time- and frequency-based as well as nonlinear HRV parameters between individuals with MDD and healthy controls were reported and analyzed. The time-based HRV parameters include the root mean sum of squared distance (RMSSD) and the standard deviation of the normal-to-normal (NN) interval (SDNN). The frequency-based HRV parameters are low-frequency HRV (LF), high-frequency HRV (HF), and ratio of low-frequency and high-frequency HRV (LF/HF ratio). The nonlinear HRV parameters include detrended fluctuations analyses (DFA), entropy-based measures (EBM) and a summarized effect size for nonlinear parameters that do not belong to DFA or EBM [54].

In part III, the effect sizes of changes in different HRV parameters such as time- and frequency-based or nonlinear parameters as well as resting heart rate due to training were reported and analyzed. The time-based parameters include RMSSD, SDNN, mean of the NN interval (mean NN), proportion of NN50 (intervals larger than 50 ms) divided by the total number of NN intervals (pNN50), expiratory–inspiratory ratio (E:I ratio) of respiratory dependence of HRV at rest, and ratio of the sympathetic and parasympathetic nervous systems during orthostatic adaptation (30:15 ratio). The frequency-based parameters include LF, HF, and LF/HF ratio. Nonlinear HRV parameters include standard deviation of the instantaneous beat-to-beat variability (SD1).

In parts II and III, several reviews also provide scores that indicate overall effects for total HRV, total time-based HRV parameters, total frequency-based HRV parameters, or total nonlinear HRV parameters. In these reviews, the effect sizes were calculated and summarized from different parameters.

## Results

### Literature Search Results

Out of an initial 14,526 search hits, 6460 were excluded as duplicates. A total of 8066 titles and abstracts were screened, resulting in 653 full texts. Of these, 90 reviews were included in our analyses (Fig. 2) [11, 21, 30, 35, 54–139]. Both reviewers had a proportionate moderate agreement for the title and abstract screening of 87% (Cohen`s kappa *κ* = 0.42) and a proportionate strong agreement for full-text screening of 95% (Cohen`s kappa *κ* = 0.80).

### Quality Rating Results

The AMSTAR 2 quality rating for each review is listed in Supplement 4 in the ESM. In part I, four reviews were of high, 14 of moderate, 25 of low, and 25 of critically low quality. In part II, two reviews were of moderate and four reviews were of critically low quality. In part III, one review was of moderate, three were of low, and ten reviews of critically low quality.

### Overview of the Research

#### Part I: Physical Activity and Major Depressive Disorder

Sixty-eight reviews were included on the influence of PA on depression severity in individuals with MDD (Supplement 5, ESM). Of these, 52 were MAs [11, 55, 58, 59, 61–64, 66, 67, 72–77, 80–83, 85, 86, 90, 93–95, 99–102, 105, 106, 109, 110, 115, 117, 119, 120, 122, 123, 125–128, 130, 131, 133–135, 138, 139] and 16 were SRs without quantitative analysis [57, 60, 65, 68–70, 84, 87–89, 91, 103, 104, 107, 118, 124]. The reviews were published over a time period of 26 years. The oldest review was published in 1998 [64], the most recent in 2024 [125, 127, 133–135]. Of the 68 reviews, six examined the effectiveness of aerobic exercise [55, 67, 86, 95, 107, 128], two of resistance exercise [59, 133], 15 of mind–body exercise [62, 65, 66, 72, 89, 91, 109, 110, 120, 122, 127, 130, 131, 138, 139], two of dance therapy [77, 118], one of mixed training [124], 40 of several PA interventions [11, 57, 58, 60, 61, 63, 64, 68–70, 73, 74, 76, 80–85, 87, 88, 90, 93, 94, 99–106, 115–117, 123, 125, 126, 134, 135] and two without a clear definition of PA [75, 119]. There was no age restriction in 55 reviews and 13 reviews focused on older adults (≥ 60 years) with MDD [57–62, 70, 74, 75, 94, 100, 103, 134].

Thirty (out of 52) MAs showed a reduction in depression severity [11, 55, 58, 61, 62, 64, 66, 72, 73, 77, 83, 86, 90, 93–95, 99, 101, 105, 110, 117, 120, 125, 126, 131, 133–135, 138, 139]. Twenty MAs reported heterogeneous results depending on the specific analysis [59, 63, 67, 74, 76, 80–82, 85, 102, 106, 109, 115, 116, 119, 122, 123, 127, 128, 130]. Two MAs did not show a reduction in depression severity in any analysis [75, 100]. Of the 16 SRs, 11 had positive conclusions in favor of the use of PA to reduce depression severity [60, 69, 70, 84, 87, 89, 103, 104, 107, 118, 124]. Five SRs ranged from positive to no effectiveness of the use of PA in reducing depression severity [57, 65, 68, 88, 91]. Since we found and included a large number of reviews, we decided to only present the moderate and high quality reviews in more detail (*n* = 18) in the following sections [11, 63, 66, 80, 85, 116, 117, 119, 120, 122, 123, 126, 128, 130, 131, 133, 134, 139].

**Fig. 3:**
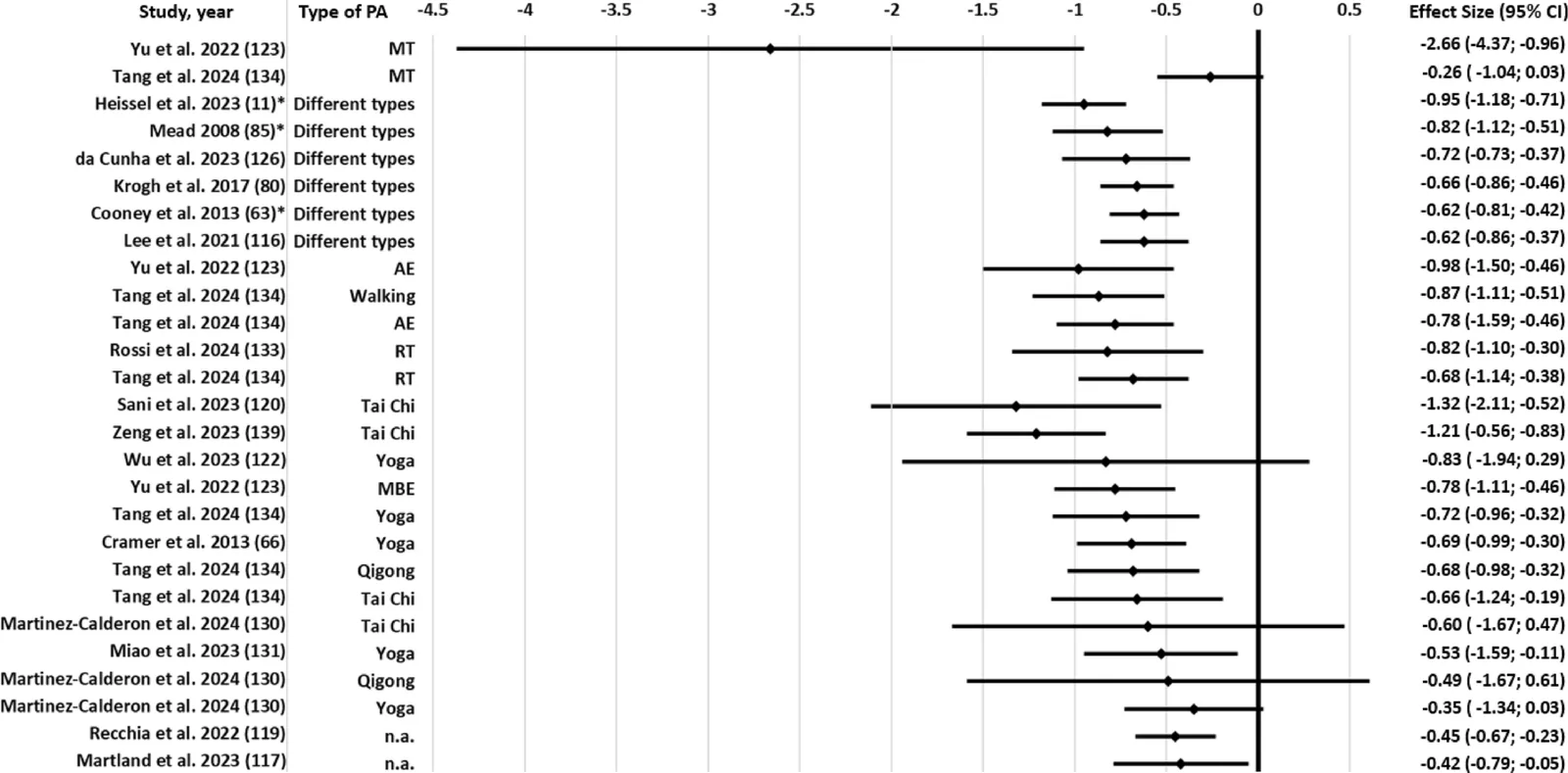
Analyses of moderate to high quality reviews of the effect sizes with 95% confidence intervals of physical activity interventions for major depressive disorder against control groups. High quality studies are superscript with *. *AE* aerobic exercise, *CI* confidence interval, *MBE* mind–body exercise, *MT* mixed training, *n.a.* not available, *PA* physical activity, *RT* resistance training

*Physical activity versus control* The effect sizes of the moderate and high-quality MAs are shown in Fig. 3. The effect sizes of the three high-quality reviews ranged from − 0.95 to − 0.62, indicating a reduction in depression severity through PA compared with control groups [11, 63, 85]. These three reviews included different types of PA and analyzed them together. The effect sizes are supported by the studies of moderate quality considering different types of PA [80, 116, 126].

When considering specific types of PA, effect sizes ranged from − 1.32 to − 0.35 [66, 120, 122, 123, 130, 131, 134, 139] for mind–body exercise including yoga, tai chi and qigong. Two reviews reported effect sizes of − 0.98 to − 0.78 for aerobic exercise [123, 134]. Two reviews analyzed resistance training with effect sizes from − 0.82 to − 0.68 [133, 134]. For mixed training, effect sizes ranging from − 2.66 to − 0.26 for reducing depression severity were reported [123, 134].

**Fig. 4:**
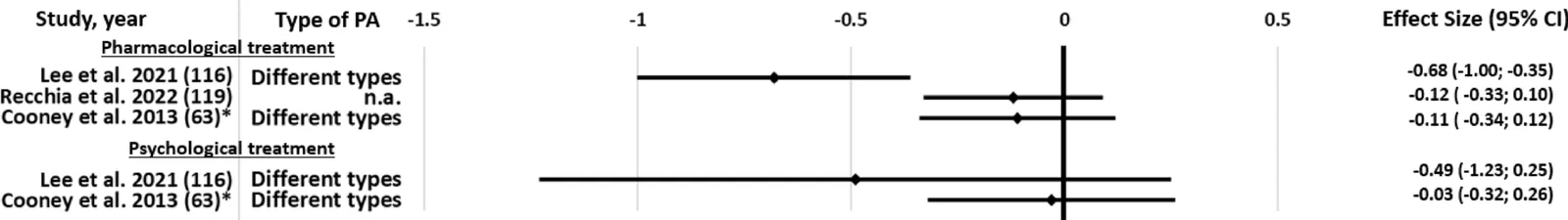
Analyses of moderate to high quality reviews of the effect sizes with 95% confidence intervals of physical activity interventions for major depressive disorder against pharmacological and psychological treatment. High quality studies are superscript with *. *CI* confidence interval, *n.a.* not available, *PA* physical activity

*Physical activity versus pharmacological treatment* The effect sizes of the moderate and high quality MAs are shown in Fig. 4. Based on three reviews, effect sizes ranged from − 0.68 to − 0.11, indicating a slightly larger reduction in depression severity with PA compared with pharmacological treatment [63, 116, 119]. Another review shows a higher remission rate with the combined use of aerobic exercise and second-generation antidepressants compared with second-generation antidepressants only. However, no difference could be demonstrated between only aerobic exercise and second-generation antidepressants [128].

*Physical activity versus psychological treatment* The effect sizes of the moderate and high-quality MAs are shown in Fig. 4. Two moderate to high quality MAs show effect sizes ranging from − 0.49 to − 0.03 for a reduction in depression severity with PA compared with psychological treatment [63, 116].

#### Part II: Heart Rate Variability and Major Depressive Disorder

**Fig. 5:**
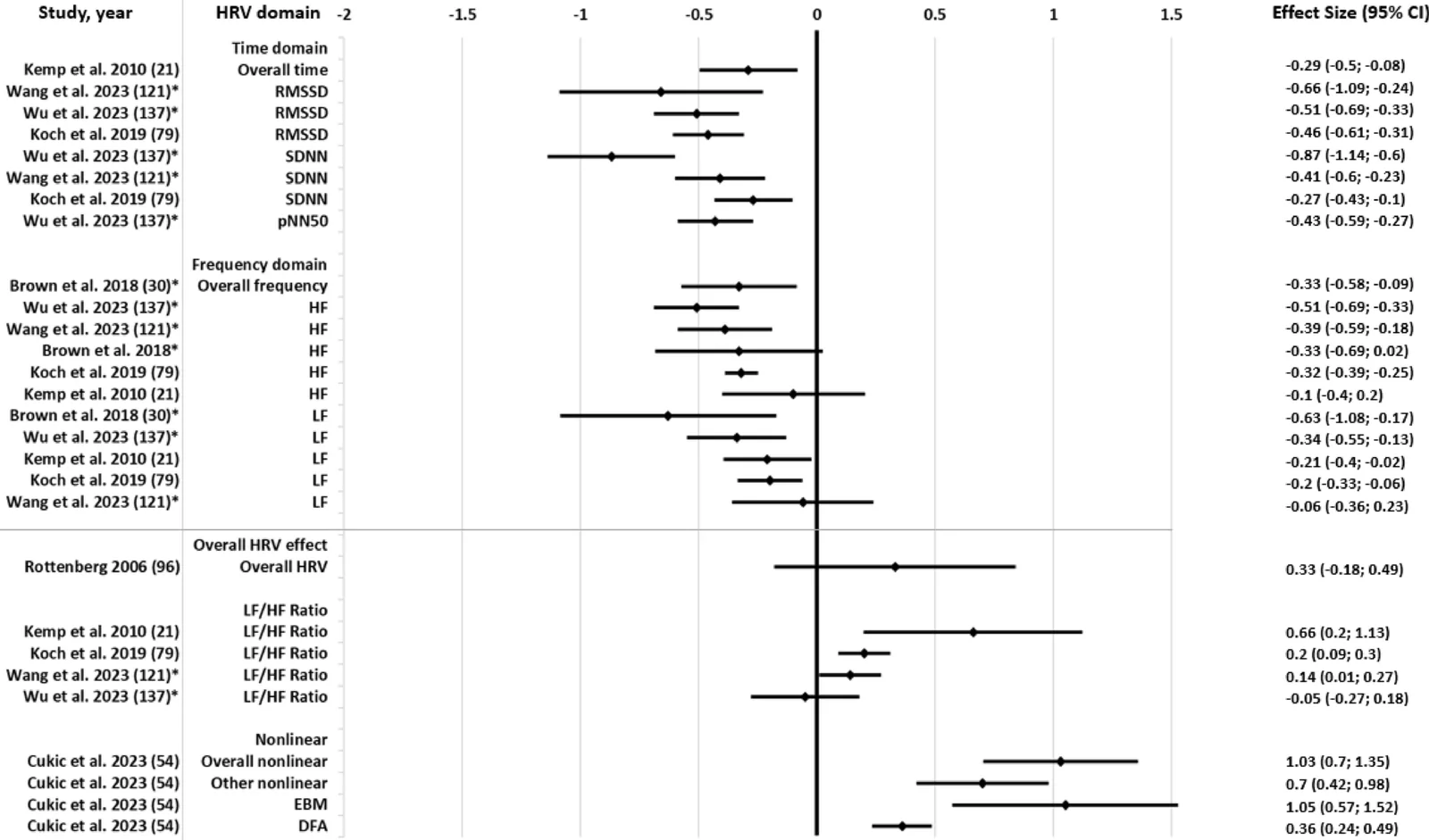
Analysis of the associations between heart rate variability and major depressive disorder with effect sizes and 95% confidence intervals, separately for different heart rate variability parameters. Moderate quality studies are superscript with *. *CI* confidence interval, *DFA* detrended fluctuation analysis, *EBM* entropy-based measures, *HF* high-frequency HRV, *HRV* heart rate variability, *LF* low-frequency HRV, *LF/HF Ratio* ratio of low-frequency to high-frequency HRV, *NN* normal-to-normal, *pNN50* proportion of NN50 divided by the total number of NN intervals, *RMSSD* root mean sum of squared distances, *SDNN* standard deviation of the NN interval

Eight reviews were included on the relationship between HRV and MDD (Supplement 6, ESM). Seven reviews were MAs [21, 30, 54, 79, 96, 121, 137] and one a SR [132]. The oldest review is from 2006 [96], the newest are from 2023 [54, 121, 132, 137]. One MA included only older adults (≥ 60 y) [30] and seven included adults with no age restrictions [21, 54, 79, 96, 121, 132, 137]. Four MAs showed a negative association between MDD and HRV [21, 30, 54, 79]. Two MAs showed a negative or negligible association with MDD depending on the respective HRV parameter [121, 137]. One MA pointed towards a negligible difference in overall HRV between healthy individuals and individuals with MDD [96]. Figure 5 shows the effect sizes of the seven MAs divided into different domains and parameters.

*Time-based parameters* In the reviews with moderate quality, the time-based parameters were lower in individuals with MDD compared with healthy individuals [121, 137]. This was supported by the other reviews with critically low quality [21, 79]. Effect sizes for lower HRV values ranged from − 0.66 to − 0.46 for RMSSD [79, 121, 137] and from − 0.87 to − 0.27 for SDNN [79, 121, 137], and for pNN50 was − 0.43.

*Frequency-based parameters* The frequency-based parameters were lower in individuals with MDD compared with healthy individuals in the moderate quality reviews [30, 121]. This was supported by the other reviews of critically low quality [21, 79, 137]. The effect sizes of the reviews with moderate quality ranged from − 0.51 to − 0.33 for HF [30, 121, 137] and − 0.32 to − 0.1 for the reviews with critically low quality [21, 79]. For LF, effect sizes for the reviews with moderate quality ranged from − 0.63 to − 0.06 [30, 121, 137]. Effect sizes for reviews with critically low quality varied between − 0.21 and − 0.2 [21, 79]. In addition, the LF/HF ratio was increased in individuals with MDD compared with healthy individuals; effect sizes ranged from − 0.05 to 0.66 [21, 79, 121, 137].

*Nonlinear parameters* The nonlinear parameters were reported in one MA [54]. Four different analyses were performed. Individuals with MDD showed higher effect sizes for differences in overall nonlinear parameters as well as the parameters of detrended fluctuations analyses (DFA) and entropy-based measures (EBM).

#### Part III: Physical Activity and Heart Rate Variability

Fourteen reviews were included in the examination of the association between PA and HRV in healthy individuals (Supplement 7, ESM) [35, 56, 71, 78, 92, 97, 98, 108, 111–114, 129, 136]. Of these, seven were MAs [35, 56, 92, 97, 111, 129, 136] and seven were SRs [71, 78, 98, 108, 112–114]. The oldest review is from 2005 [97], the newest reviews are from 2024 [129, 136]. Two reviews examined the effects of aerobic exercise [35, 97], three of resistance exercise [56, 78, 112], three of mind–body exercise [92, 108, 129], and six included several PA interventions [71, 98, 111, 113, 114, 136]. Four reviews included only participants with older ages (≥ 60 y) [35, 56, 98, 114] and ten included adults with no age restriction [71, 78, 92, 97, 108, 111–113, 129, 136].

**Fig. 6:**
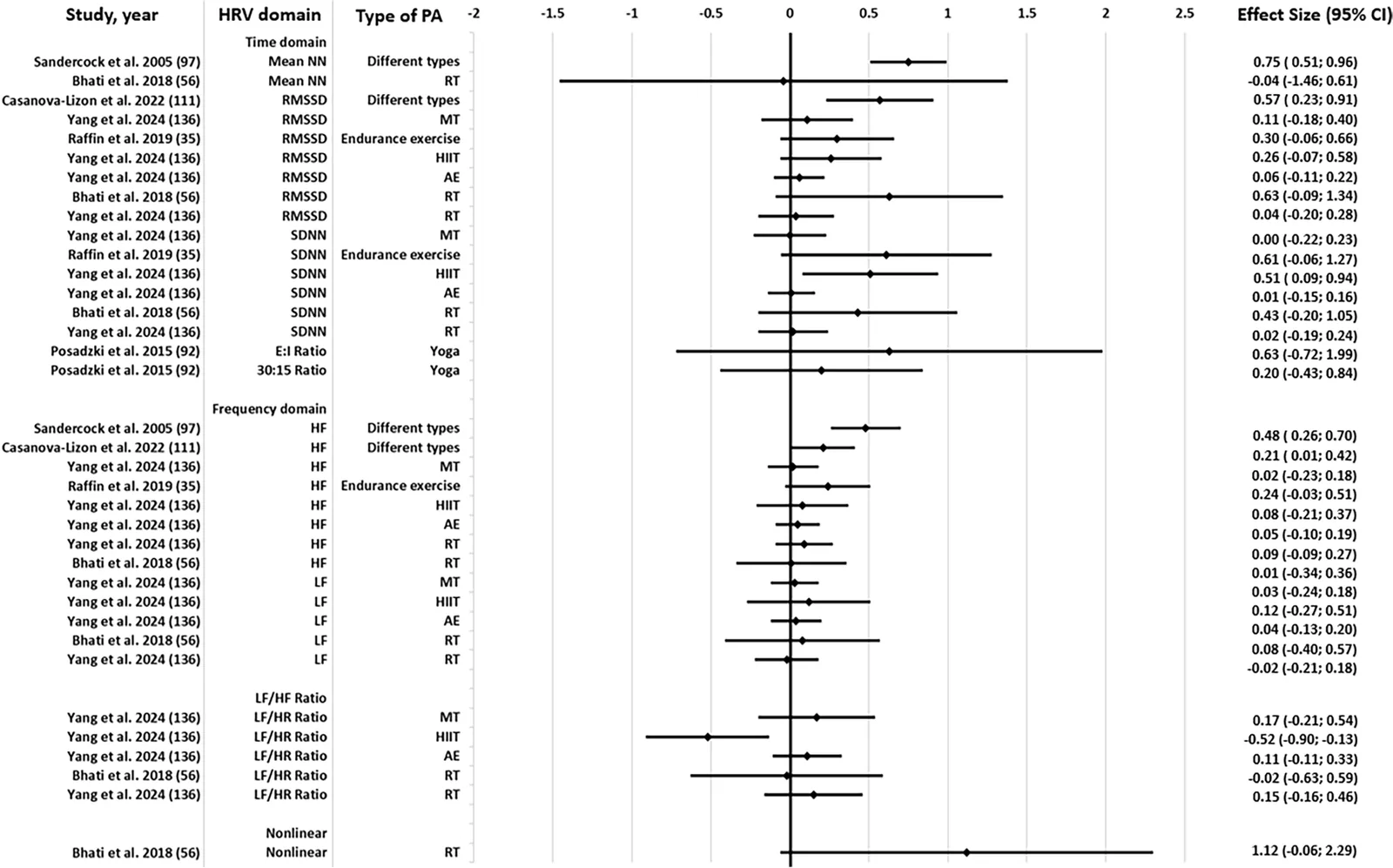
Analyses of the effect sizes with 95% confidence intervals of the effect of physical activity on heart rate variability, separately for different heart rate variability parameters. *AE* aerobic exercise, *CI* confidence interval, *E:I ratio* expiratory–inspiratory ratio, *HF* high-frequency HRV, *HIIT* high-intensity interval training, *LF* Low-frequency HRV, *LF/HF Ratio* ratio of low-frequency to high-frequency HRV, *mean NN* mean of the NN interval, *MT* mixed training, *NN* normal-to-normal, *PA* physical activity, *RMSSD* root mean sum of squared distances, *RT* resistance training, *SDNN* standard deviation of the NN interval, *30:15 ratio* ratio of the sympathetic and parasympathetic nervous systems during orthostatic adaptation

Overall, of the seven MAs, three showed positive effects for an improvement on HRV trough PA [97, 111, 129] and four showed positive or negligible effects in the analyses, depending on the parameter [35, 56, 92, 136]. Of the seven SRs, two SRs drew positive conclusions for an improvement [113, 114], three SRs mixed conclusions with positive and negligible effects of PA on HRV [71, 98, 108] and two SRs [78, 112] found negligible effects. Figure 6 shows the effect sizes of the seven MAs divided into different parameters.

*Time-based parameters* Different types of PA can improve time-based parameters. Mean NN can be increased by combining different types of PA in the analyses, and by endurance exercise in the form of interval training, but not by resistance exercise alone [56, 97, 98]. Several types of PA can increase RMSSD with effect sizes ranging from 0.04 to 0.63 [35, 56, 111, 136]. This range is supported qualitatively in the SRs. Endurance exercise, yoga and different types of PA can increase RMSSD, but not resistance exercise [71, 98, 108, 112, 114]. The effect sizes for SDNN ranged from 0 to 0.61 [35, 56, 136]. Qualitatively, the results are also heterogeneous. Yoga may increase SDNN [108], whereas endurance interval training may decrease it [98]. The E:I ratio and the 30:15 ratio can be increased and improved by yoga [92].

Qualitative evaluations on the combined time-based parameters are also presented. It is shown that time-based parameters can be improved by combining different types of PA in young, middle-aged and older adults [71, 113, 114]. Yoga may improve time-based parameters, but the evidence is inconsistent [108]. Resistance exercise seems to have no beneficial effect on combined time-based parameters in adults and older adults [78, 112], whereas yoga may decrease resting heart rate [129].

*Frequency-based parameters* Different types of PA and aerobic exercise can positively influence frequency-based parameters, whereas resistance exercise does not. Effect sizes for HF ranged from 0.01 to 0.48 [35, 56, 97, 111, 136]. Qualitative analyses indicate that resistance exercise does not have a positive effect on HF [112], and nor can other frequency-based parameters be improved by resistance exercise [56, 78, 112]. For various types of PA and yoga, the picture is different. Frequency-based parameters improved [71, 108, 114]. The LF/HF ratio can also be affected by PA, with effect sizes ranging from − 0.52 to 0.17 [56, 136].

*Nonlinear parameters* Nonlinear parameters can be positively influenced by resistance exercise and different types of PA, as shown in one MA and two SRs with combined nonlinear parameters [56, 71, 113] and in one SR with parameters SD1 and SD2 [114].

## Discussion

### Principal Findings

**Fig. 7:**
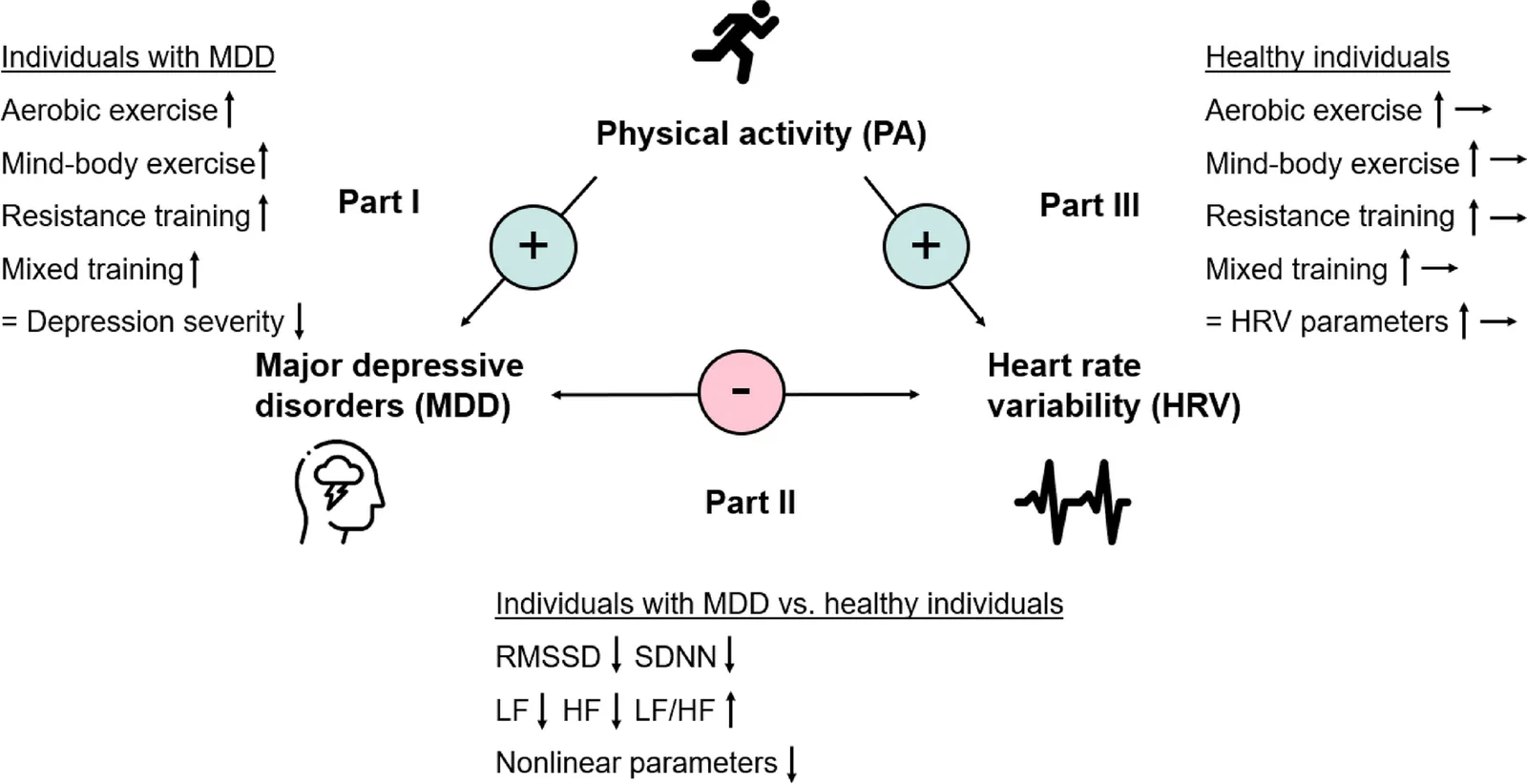
Interaction between physical activity, major depressive disorder and heart rate variability. *HF* high-frequency HRV, *HRV* heart rate variability, *LF* low-frequency HRV, *LF/HF Ratio* ratio of low-frequency to high-frequency HRV, *NN* normal-to-normal, *RMSSD* root mean sum of squared distances, *SDNN* standard deviation of the NN interval

This umbrella review aimed to investigate the complex interrelationship between MDD, PA, and HRV. It analyzed how PA affects depression severity and HRV, as well as the relationship between HRV and MDD. In general, it could be demonstrated that various forms of PA are effective in reducing depression severity in individuals with MDD. It seems that PA is potentially as beneficial as pharmacological or psychotherapeutic interventions in reducing severity of depression. Furthermore, it has been shown that HRV is negatively affected in individuals with MDD with reduced time-based, frequency-based, and nonlinear parameters. In healthy individuals, PA can positively influence HRV parameters, but there are differences depending on the parameter and the form of PA. Figure 7 summarizes the findings of this umbrella review.

### Physical Activity and Major Depressive Disorder

PA is an effective method for reducing depression severity in individuals with MDD. Our findings are supported by the comprehensive umbrella review by Singh et al. [43]. They showed that PA reduced severity of depression in healthy individuals and in individuals with various health conditions, with the largest effects being observed in individuals with MDD [43].

Our review demonstrates that PA could be as effective as pharmacological and psychological interventions. PA potentially affects the remission rate in the same way or better than, for example, second-generation antidepressants. In combination with second-generation antidepressants, PA even appears to be superior to second-generation antidepressants therapy alone [128]. Therefore, the use of PA in the treatment of MDD is strongly recommended [1]. In addition to psychological effects, further positive health effects can be achieved through PA, such as the improvement of cardiovascular health [140], musculo-skeletal performance [141], or brain functions [142]. All these effects have a positive impact on the quality of life of individuals suffering from MDD [141].

Many different types of PA are effective in reducing depression severity. Our umbrella review shows that mixed training, mind–body exercise, aerobic exercise and resistance exercise, among others, are beneficial. This is consistent with other literature [18, 43, 143, 144]. A recent SR with network meta-analysis showed that dance therapy, walking/jogging and yoga are particularly effective in reducing depression severity in individuals with MDD and different health conditions [18].

Our data show that PA is especially superior to control conditions, such as no treatment or treatment as usual. The review by Lawlor and Hopker [82] already showed in 2001 that PA can reduce the Beck Depression Inventory, second edition (BDI-II) score by an average of − 7.3 (95% CI − 10.0 to − 4.6) points. This is confirmed by the current review by Heissel et al. [11], which reports an average reduction in BDI-II of − 6.5 (95% CI − 8.6 to − 4.4) points. Depending on the baseline severity of the depression, this is clinically relevant since, for example, a severe depression severity begins at 29 points [154]. Depending on the classification, a remission can be classified as below 13 points in somatic groups or 19 points in the case of comorbid psychiatric illness [155]. As shown in our review, there is a substantial number of SRs and MAs that demonstrate the efficacy of PA. Although there is a large amount of evidence on the efficacy of PA in individuals with MDD, research on the feasibility of PA promotion or sports programs, on the best combination with other therapies and potential mechanisms of action is particularly needed. In conclusion, it can be stated that various forms of PA are effective in reducing depression severity and it is important to integrate PA in therapy.

### Heart Rate Variability and Major Depressive Disorder

The summarized reviews clearly show that there are differences in ANS, as measured by HRV, between healthy individuals and individuals with MDD. The time- and frequency-based parameters as well as the nonlinear parameters were lower in individuals with MDD. These findings are also present in other affective disorders, such as bipolar depression [156].

The reduced time-based parameters in individuals with MDD are reflected in lower RMSSD and SDNN values. Lower RMSSD values are supposed to indicate lower parasympathetic activity. This can be explained by a reduced vagal tone [29]. Lower vagal activity is associated with dysregulated recovery of cardiovascular, endocrine, and immune functions [157]. In general, the parameter SDNN represents the function of the sympathetic and parasympathetic systems. In particular, short-term recordings of RMSSD, such as those included in this review, are supposed to primarily indicate parasympathetic activity [29].

The frequency-based parameters are characterized by lower HF and LF values. The lower HF values indicate lower parasympathetic with reduced efferent vagal activity [29]. Lower HF values correlate with panic, stress and anxiety [33]. A lower LF and the increased LF/HF ratios are more difficult to interpret. So far, it was assumed that the LF values represent sympathetic activity. However, studies show that the LF parameter is more complex and that parasympathetic and sympathetic activity both affect it [158, 159]. Low LF values suggest an impairment of parasympathetic and sympathetic activity of the ANS. Therefore, the LF/HF ratio cannot be considered primarily as a ratio of sympathetic to parasympathetic nervous system. The exact meaning of this parameter is still being discussed [160].

The nonlinear parameters also show a difference between individuals with MDD and healthy individuals. In the included review, higher effect sizes represent higher abnormalities, which are characterized by lower HRV values [54]. For detrended fluctuations analyses (DFA) and entropy-based measures (EBM), the higher effect sizes indicate a negative profile in baroreceptor reflex, regulatory mechanisms and less variability in NN intervals [29]. These nonlinear parameters are used much less frequently than time-based or frequency-based parameters. Thus, a comparison with other studies is difficult.

In summary, individuals with MDD have reduced vagal activity and exhibit lower parasympathetic activity. However, the exact associations between impaired ANS and MDD are still unclear. Three longitudinal cohort studies [24, 161, 162] and the two reviews by Gullett et al. [31] and Galin and Keren [163] come closer to possible explanations. First, it was shown that a higher resting heart rate and lower HRV values at baseline are predictors of higher depression severity in men and potentially in women. Secondly, it was shown that the association between autonomic dysregulation and depression is bidirectional, but stronger for the assumption that impaired autonomic function precedes MDD than vice versa. It was shown that lower HRV values at baseline were associated with increased depression severity at follow-up, regardless of health condition or use of medication [161]. Thirdly, it was demonstrated that in young healthy adults, negatively influenced vagal activity as a marker for HRV at baseline could predict higher depressive symptoms at follow-up [162]. The review by Gullett et al. [31] has shown that in healthy individuals, negative affect (i.e. the experience of negative emotions or a negative state of mind such as feeling anxious) has a negative impact on HRV. They were able to show that an elevated negative affect level leads to a reduction in parasympathetic activity [31]. A chronic reduction in parasympathetic activity can lead to inflammatory processes [164], which represent a risk for MDD [165, 166]. Further prospective cohort studies are therefore needed to investigate the link between HRV and MDD more clearly.

### Physical Activity and Heart Rate Variability

Overall, various forms of PA improve time-based HRV parameters in healthy individuals. Endurance exercise and the combination of different types of exercise are especially effective. They increase vagal activity and also increase the parasympathetic and sympathetic activity of the ANS [29]. Furthermore, endurance training improves global cardiac autonomic function [35] and potentially reduces resting heart rate [167]. Resistance training has different effects. Bhati et al. [56] and Raffin et al. [35] show positive effects on RMSSD and SDNN, but the review by Yang et al. [136] cannot confirm this. The review by Yang et al. [136] shows negligible effects for several types of PA, which is partly in contradiction to other reviews. The review differs from the others in that it also included Chinese-language literature.

Endurance exercise and various types of PA can positively influence the frequency-based parameter HF. This indicates increased vagal activity which represents a higher activity of the vagus nerve and thus a better regulation of vital functions [29]. In contrast, resistance training shows negligible changes in frequency-based parameters in the reviews [56, 136]. As with the time-based parameters, the review by Yang et al. [136] shows substantially smaller effect sizes for the frequency-based parameters, especially for HF values. All reviews show that PA interventions do not increase LF values. These findings in this umbrella review apply to healthy adults. However, it may be different in individuals with chronic diseases, such as cardiovascular diseases. For instance, it has been shown that LF values can be improved by PA in such patients [168].

In summary, it can be stated that PA may positively influence parasympathetic and thus vagal activity in healthy individuals as reflected in the time-based parameter RMSSD and the frequency-based parameter HF. Improved parasympathetic modulation can lead to a reduction in mortality risk [97, 111]. In addition, it should be noted that there is an increased potential for improvement of these HRV parameters in vulnerable groups [56, 97, 111]. Furthermore, the intensity and frequency of PA sessions should also be considered. Casanova-Lizon et al. [111] describe how moderate PA provides a sufficient stimulus for improving HRV, but that high-intensity PA can be more effective and can help to maintain an improvement. Raffin et al. [35] show that training volume and training frequency are crucial in older individuals, as it may not be possible to further increase intensity, which is why increasing frequency leads to the desired increase in volume. In conclusion, parasympathetic activity can be positively influenced by various forms and intensities of PA.

### Mechanisms of Physical Activity on Heart Rate Variability and Major Depressive Disorder

**Fig. 8:**
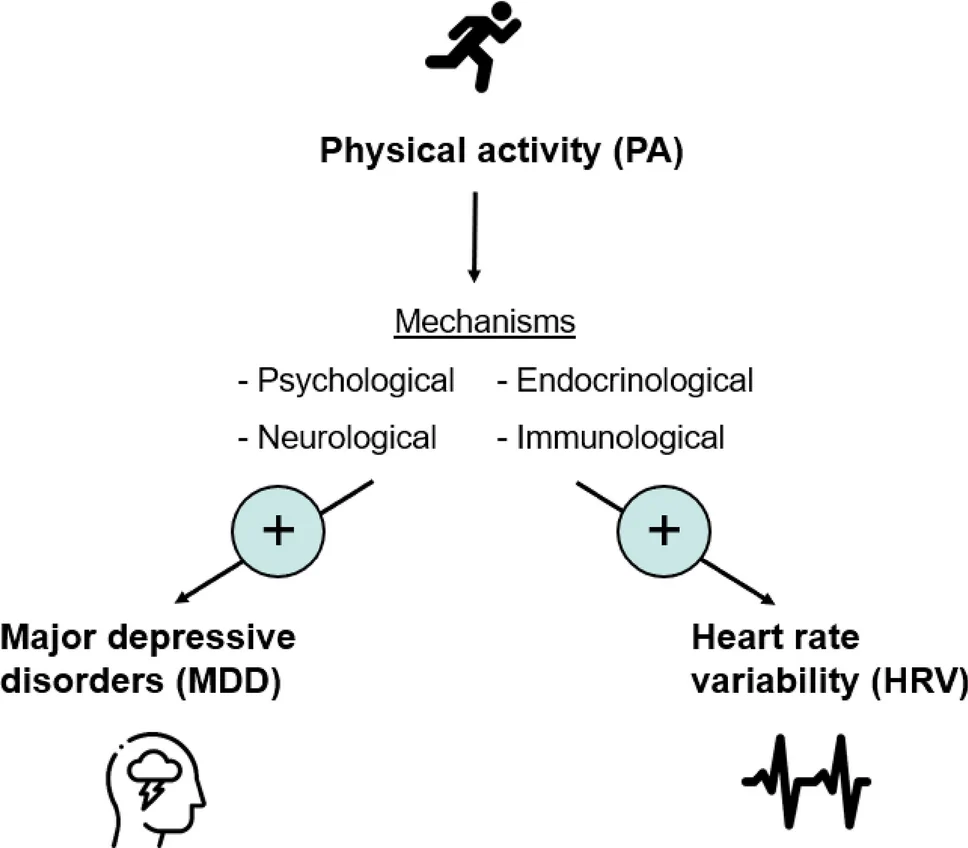
Mechanisms of the effects of physical activity on major depressive disorder and heart rate variability

There are various explanations as to why PA can alleviate symptoms of depression. One explanation is based on social mechanisms [145]. In general, PA can improve self-esteem, self-respect, self-efficacy, and self-confidence when positive feedback is received from PA [146–148]. Further explanations, such as psychological and neurophysiological mechanisms, apply not only to the reduction of depression symptoms [145], but also to the increase in HRV through PA. Psychological mechanisms include affect regulation and cognitive control via the prefrontal cortex [169]. These are reduced in MDD [170]. PA can increase these, which is associated with an improvement in HRV and leads to better emotion regulation [170–173]. Neurophysiological mechanisms include improved modulation of the HPA axis, which can be dysregulated in MDD and is closely linked to the ANS [174, 175]. PA can improve cortisol regulation and HRV [176, 177]. Improved neuroplasticity and neurogenesis are also among the benefits. PA can increase brain-derived neurotrophic factor (BDNF) levels, which has a positive effect on neuroplasticity and can also indirectly influence HRV [178–180]. PA can also have an anti-inflammatory effect. Low HRV is associated with elevated inflammation levels, and PA can reduce these, which correlates with an improvement in the severity of depression [149, 181–183]. In summary, PA can have a positive effect on psychological, endocrinological, neurological, and immunological mechanisms, which in turn can have a positive impact on improving HRV and depression severity and is shown in Fig. 8. Aerobic exercise has beneficial effects on all four categories of mechanisms [114, 184–186]. Resistance training primarily results in positive inflammatory and neurological mechanisms [184, 187]. Mind–body exercise particularly has positive effects on psychological, endocrine, and neurological mechanisms [184, 187]. In the future, research should be conducted to determine which types of PA have a positive effect on both MDD and HRV. In addition, further parameters should be evaluated to gain a better understanding of the mechanisms involved.

### Primary Studies of the Effects of Physical Activity on Heart Rate Variability in Major Depressive Disorder

So far, there have only been a few studies that have examined how PA can affect HRV in individuals with MDD. The SR with MA by Chen et al. [188] included only one randomized controlled trial from Toni et al. [23] for PA as a therapy. This shows that in older patients with MDD, pharmacological therapy as well as a combination of pharmacological therapy and PA can improve several HRV parameters including RMSSD, SDNN, HF, and LF over a 24-week period, whereby the combination is more effective [23]. Two other randomized controlled trials from Chu et al. [41] and Liu et al. [42] suggest that mind–body exercise may be effective in improving HRV in individuals with MDD. Tai chi improved HRV as indicated by resting heart rate, RMSSD, and HF in older individuals with MDD compared with the control group after a 24-week intervention [42]. Yoga improved the HRV parameter HF in women with MDD compared with controls in a 12-week intervention [41]. These three studies show that there is potential to improve HRV in individuals with MDD, but further studies of high quality are needed to show which types and intensities of PA are effective in this population. It should be noted that the two studies of mind–body exercise used small sample sizes [41, 42], and that the three studies did not have a follow-up for a period of time after the intervention [23, 41, 42]. Furthermore, the results of the three interventions cannot be generalized to men and women with MDD of different ages, as either older individuals [23, 42] or only women [41] were included. In addition, the interventions were carried out in group settings, so other factors such as social interaction between participants or a lack of blinding or increased attention by instructors may have biased the results.

### Future Studies of the Effects of Physical Activity on Heart Rate Variability in Major Depressive Disorder

Randomized controlled trials are needed in the future to investigate the effects of PA on HRV and MDD. These should include a clear intervention description of PA so that the intervention is reproducible. Established guidelines such as the Consensus on Exercise Reporting Template (CERT) should be followed [189]. This will allow comparison with active control groups or treatment as usual. In addition, other therapies should be recorded alongside the intervention and, if necessary, controlled for. It is known that psychological interventions can also have a positive effect on HRV [188] and that antidepressants can have both positive and negative effects on HRV [21, 190, 191]. Stratified randomization should therefore ideally be based on age, sex, severity of depression, and medication taken [192]. In addition, adequate statistical power with sufficiently large samples is required, which can be generated, for example, by multicenter studies [193]. HRV should be measured in a standardized manner. The review by Laborde et al. [194], which is based on the Task Force Guidelines on measuring HRV [195], is a useful guide for this. This allows short-term measurements of 5 min to be implemented. It is important to control confounding factors such as body position, recovery period before measurement, time of day, caffeine, medication, and, depending on the measurement parameter, respiratory rate [194]. Since MDD is often associated with a reduction in parasympathetic activity, at least RMSSD and HF should be measured. Furthermore, the severity of depression should be assessed using validated and widely implemented instruments such as the PHQ-9 [196, 197], BDI-II [154, 155] or the HAMD-17 [198, 199]. This allows the assessment of affective and somatic symptoms and comparison with other studies. Furthermore, short-term effects at the end of the intervention and long-term effects at follow-up should be measured. To better understand the possible mechanisms of action, potential mediators should be measured, such as inflammation markers like CRP [200], markers for neuroplasticity such as BDNF [201], or cortisol for HPA axis activity [175]. In this context, it is also worthwhile collecting moderators such as HRV, MDD severity, fitness, or current level of PA at baseline to control for these markers.

### Implementation of Physical Activity to Improve Heart Rate Variability and Major Depressive Disorder

The evidence clearly supports that PA can reduce depression severity and improve several HRV parameters. Despite these obvious findings, there are challenges in implementing PA. For example, dropout rates for PA interventions in individuals with MDD are higher than for pharmacological therapy (risk ratio 1.31; 95% CI 1.09 to 1.57) [119]. In addition, there are other factors that make adherence to PA interventions more difficult. For example, tobacco use or alcohol abuse, which have an increased prevalence in individuals with MDD [202], reduce adherence in this population [203]. Therefore, a PA intervention leads to a positive outcome “only if the pill is taken” [204].

To increase the ‘intake’, the implementation of PA should be adapted to individual needs. This concerns, for example, intensity, since moderate and high intensity are effective in improving MDD severity and HRV values [11, 111]. However, intensities are perceived and accepted differently [205]. A single session can lead to positive affect, but the likelihood of this is higher with moderate intensity compared with high intensity [206, 207]. Furthermore, it depends on the duration. It is beneficial to aim for at least 150 min of moderate activity per week [125], but progression must be individualized. In addition, the setting may be important. For example, a group setting can be favored because social interaction may increase adherence [125, 208]. In addition, the type of PA plays a key role. As our review shows, different types are effective in reducing depressive severity and improving HRV. Being able to choose a suitable activity autonomously increases the chance of maintaining it [209]. By paying attention to these points, the likelihood can be improved that individuals with MDD will become more physically active, and that depression severity and HRV can be positively affected in individuals with MDD [188].

Our review shows that aerobic exercise and a mix of different types of exercise are particularly effective in positively influencing HRV. The primary studies also show that mind–body exercises such as yoga could be effective in improving HRV and depressive symptoms in individuals with MDD [41, 42]. In addition, resistance exercises may also have a positive effect on HRV in MDD, even though the effects are mostly negligible in healthy controls. Therefore, future research should focus more on implementation and efficiency rather than the effectiveness of exercise interventions in the field of MDD.

### Quality of Reviews

In general, there were large differences between the three analyses in the number and quality of included reviews. The analysis of PA and MDD included substantially more SRs and MAs and had the largest number of high-quality reviews. The analyses of PA and HRV as well as MDD and HRV were comparable in number and quality of reviews. Nevertheless, the quality of the studies has increased over the years. This is particularly evident in the analysis of PA and MDD, where 15 of the 18 studies of high to moderate quality were published in 2021 or later. This may be related to the fact that journals have made certain quality criteria mandatory only in recent years, such as the use of the PRISMA guidelines [48]. Another reason for the increase in quality may be the implementation of the AMSTAR-2 criteria in 2017 [53].

### Methodological Considerations

The main strength of this umbrella review is the comprehensive literature search and analysis of the three parts with the parameters PA, MDD and HRV. The analysis shows that there is an inflation of SRs and MAs, especially in part I with MDD and PA, which is why other authors are also arguing for a shift towards less ‘conversation’ and more ‘action’ [210]. In addition, part I is only based on reviews with individuals with MDD, thus improving external validity. Moreover, we defined our search criteria and processes in advance. We worked according to the PRISMA guidelines and classified the included reviews according to the AMSTAR-2 criteria.

The limitations of this review should also be mentioned. In part I, we summarized placebo control conditions and treatment as usual in the analysis of PA for depression severity in individuals with MDD as controls. Since treatment as usual is also interpreted differently in the included reviews, treatment as usual may have a different influence on the results in the reviews [211]. In order to provide a broad overview, we included studies with a wide range of HRV parameters in parts II and III. This makes it difficult to compare the results. In part III, we included only healthy individuals and no individuals with MDD to demonstrate the effects of PA on HRV. We made this decision because we wanted to take a broad overview of how PA can affect HRV. In addition, no method was used to minimize overlap between the primary studies of the included SRs and MAs. This was omitted because no overview MA of the included data with pooled data was performed, and a broad overview of the current study evidence was to be presented. Moreover, we were only able to include literature in English and German. It is therefore possible that additional knowledge in other languages was not included in this umbrella review. Finally, we could only report on what has already been reported in other reviews. It is therefore possible that new scientific evidence may not have been included in our review due to a time lag.

## Conclusions and Outlook

This comprehensive umbrella review demonstrates that different types of PA are effective in reducing severity of depression in individuals with MDD and improving autonomic function in healthy individuals. In addition, HRV as a parameter of the ANS is impaired in individuals with MDD. The umbrella review shows that endurance exercise and the combination of different types of exercise are highly effective in improving HRV. This suggests that PA may also improve HRV in individuals with MDD, which few studies have even begun to show. Randomized controlled trials should be conducted in the future to examine which types and forms of PA are effective in positively affecting HRV in individuals with MDD. In addition, the function of a change in HRV for the course of therapy should be demonstrated, as well as the extent to which HRV can be used as a predictor for a successful course of therapy. Therefore, standardized parameters in the area of HRV and depression severity should be used so that comparability is even greater.

## Supplementary Information

Below is the link to the electronic supplementary material.

Supplementary file1 (PDF 1832 kb)

## Declarations

### Data Availability Statement

The data and materials will be made available by the authors upon request.
