# Supplementary material for: Association Between Physical Activity, Heart Rate Variability and Major Depressive Disorders: An Umbrella Review
Source: Sports Med. 2026 Feb 4;56(3):703–23. doi: 10.1007/s40279-025-02365-5 (PMC13710458; doi:10.1007/s40279-025-02365-5)
Supplement: Supplementary file 1 [file 40279_2025_2365_MOESM1_ESM.pdf]

Supplementary material

An umbrella review on the association between physical activity, heart rate variability and major depressive disorders

Jan-Niklas Kreppke[1]*, **Gavin Brupbacher[2,3], Kerstin Rupf[1], Vivien Hohberg[1], Robyn Cody[1,4], Markus Gerber[1], Oliver Faude[1]**

[1] Department for Sport, Exercise and Health, University of Basel, Basel, Switzerland

[2] Clinic Oberwaid AG, St. Gallen, Switzerland

[3] Department of Consultation-Liaison Psychiatry and Psychosomatic Medicine, University Hospital Zurich, Switzerland

[4] Institute for Implementation Science in Health Care, University of Zurich, Zurich, Switzerland

* **Corresponding author**:

Dr. Jan-Niklas Kreppke

Grosse Allee 6

4052 Basel

Switzerland

jan-niklas.kreppke@unibas.ch

S1 – Supplement 1 PRISMA checklist

Section and Topic	Item #	Checklist item	Reported on page #
**TITLE**			
Title	1	Identify the report as a systematic review.	1
**ABSTRACT**			
Abstract	2	See the PRISMA 2020 for Abstracts checklist.	2
**INTRODUCTION**			
Rationale	3	Describe the rationale for the review in the context of existing knowledge.	4,5
Objectives	4	Provide an explicit statement of the objective(s) or question(s) the review addresses.	6
**METHODS**			
Eligibility criteria	5	Specify the inclusion and exclusion criteria for the review and how studies were grouped for the syntheses.	8 and Table 1
Information sources	6	Specify all databases, registers, websites, organisations, reference lists and other sources searched or consulted to identify studies. Specify the date when each source was last searched or consulted.	7
Search strategy	7	Present the full search strategies for all databases, registers and websites, including any filters and limits used.	7 and Supplement 2
Selection process	8	Specify the methods used to decide whether a study met the inclusion criteria of the review, including how many reviewers screened each record and each report retrieved, whether they worked independently, and if applicable, details of automation tools used in the process.	7
Data collection process	9	Specify the methods used to collect data from reports, including how many reviewers collected data from each report, whether they worked independently, any processes for obtaining or confirming data from study investigators, and if applicable, details of automation tools used in the process.	8,9
Data items	10a	List and define all outcomes for which data were sought. Specify whether all results that were compatible with each outcome domain in each study were sought (e.g. for all measures, time points, analyses), and if not, the methods used to decide which results to collect.	8,9,10
	10b	List and define all other variables for which data were sought (e.g. participant and intervention characteristics, funding sources). Describe any assumptions made about any missing or unclear information.	8,9,10
Study risk of bias assessment	11	Specify the methods used to assess risk of bias in the included studies, including details of the tool(s) used, how many reviewers assessed each study and whether they worked independently, and if applicable, details of automation tools used in the process.	8
Effect measures	12	Specify for each outcome the effect measure(s) (e.g. risk ratio, mean difference) used in the synthesis or presentation of results.	9
Synthesis methods	13a	Describe the processes used to decide which studies were eligible for each synthesis (e.g. tabulating the study intervention characteristics and comparing against the planned groups for each synthesis (item #5)).	8

Section and Topic	Item #	Checklist item	Reported on page #
	13b	Describe any methods required to prepare the data for presentation or synthesis, such as handling of missing summary statistics, or data conversions.	8,9
	13c	Describe any methods used to tabulate or visually display results of individual studies and syntheses.	9,10
	13d	Describe any methods used to synthesize results and provide a rationale for the choice(s). If meta-analysis was performed, describe the model(s), method(s) to identify the presence and extent of statistical heterogeneity, and software package(s) used.	n/a
	13e	Describe any methods used to explore possible causes of heterogeneity among study results (e.g. subgroup analysis, meta-regression).	8,9,10
	13f	Describe any sensitivity analyses conducted to assess robustness of the synthesized results.	n/a
Reporting bias assessment	14	Describe any methods used to assess risk of bias due to missing results in a synthesis (arising from reporting biases).	n/a
Certainty assessment	15	Describe any methods used to assess certainty (or confidence) in the body of evidence for an outcome.	8
**RESULTS**			
Study selection	16a	Describe the results of the search and selection process, from the number of records identified in the search to the number of studies included in the review, ideally using a flow diagram.	10 and Figure 2
	16b	Cite studies that might appear to meet the inclusion criteria, but which were excluded, and explain why they were excluded.	Supplement 3
Study characteristics	17	Cite each included study and present its characteristics.	10 and Supplements 5,6,7
Risk of bias in studies	18	Present assessments of risk of bias for each included study.	10 and Supplement 4
Results of individual studies	19	For all outcomes, present, for each study: (a) summary statistics for each group (where appropriate) and (b) an effect estimate and its precision (e.g. confidence/credible interval), ideally using structured tables or plots.	10-15, Figures 3,4,5,6 and Supplements 5,6,7
Results of syntheses	20a	For each synthesis, briefly summarise the characteristics and risk of bias among contributing studies.	10-15, Figures 3,4,5,6 and Supplements 5,6,7
	20b	Present results of all statistical syntheses conducted. If meta-analysis was done, present for each the summary estimate and its precision (e.g. confidence/credible interval) and measures of statistical heterogeneity. If comparing groups, describe the direction of the effect.	10-15, Figures 3,4,5,6 and

Section and Topic	Item #	Checklist item	Reported on page #
			Supplements 5,6,7
	20c	Present results of all investigations of possible causes of heterogeneity among study results.	n/a
	20d	Present results of all sensitivity analyses conducted to assess the robustness of the synthesized results.	n/a
Reporting biases	21	Present assessments of risk of bias due to missing results (arising from reporting biases) for each synthesis assessed.	n/a
Certainty of evidence	22	Present assessments of certainty (or confidence) in the body of evidence for each outcome assessed.	10 and Supplement 4
**DISCUSSION**			
Discussion	23a	Provide a general interpretation of the results in the context of other evidence.	15-19
	23b	Discuss any limitations of the evidence included in the review.	21
	23c	Discuss any limitations of the review processes used.	21
	23d	Discuss implications of the results for practice, policy, and future research.	19,20
**OTHER INFORMATION**			
Registration and protocol	24a	Provide registration information for the review, including register name and registration number, or state that the review was not registered.	3,7
	24b	Indicate where the review protocol can be accessed, or state that a protocol was not prepared.	7
	24c	Describe and explain any amendments to information provided at registration or in the protocol.	n/a
Support	25	Describe sources of financial or non-financial support for the review, and the role of the funders or sponsors in the review.	23
Competing interests	26	Declare any competing interests of review authors.	23
Availability of data, code and other materials	27	Report which of the following are publicly available and where they can be found: template data collection forms; data extracted from included studies; data used for all analyses; analytic code; any other materials used in the review.	n/a

S2 – Supplement 2 Search terms

Search component	Search string: MEDLINE via Ovid	Search string: Embase via Ovid	Search string: PsycINFO via Ovid	Search string: SPORTDiscus via EBSCO	Search string: Epistemonikos
**Search component 1: depression**	exp depressive disorder, major/ OR exp dysthymia/ OR exp melancholia/ OR dysphoria.ti,ab. OR depress*.ti,ab. OR Paraphrenia.ti,ab. OR Involutional Psychos*.ti,ab. OR Melancholia.ti,ab. OR unipolar disorder*.ti,ab. OR Dysphoria.ti,ab. OR Dysthymic Disorder*.ti,ab.	exp depressive disorder, major/ or exp dysphoria/ or exp dysthymia/ or exp melancholia/ or depress*.ti,ab. or Paraphrenia.ti,ab. or Involutional Psychos*.ti,ab. or Melancholia.ti,ab. or unipolar disorder*.ti,ab. or Dysphoria.ti,ab. or Dysthymic Disorder*.ti,ab.	exp Major Depression/ OR depress*.ti,ab. or Paraphrenia.ti,ab. or Involutional Psychos*.ti,ab. or Melancholia.ti,ab. or unipolar disorder*.ti,ab. or Dysphoria.ti,ab. or Dysthymic Disorder*.ti,ab.	DE "MENTAL depression" OR DE "DEPRESSION in college students" OR TI "depress*" OR AB "depress*" OR TI "mdd" OR AB "mdd" OR TI "paraphrenia" or AB "paraphrenia" or TI "Involutional psychos*" or AB "Involutional psychos*" or TI "melancholia" or AB "melancholia" or TI "unipolar disorder*" or AB "unipolar disorder*" or TI "dysphoria" or AB "dysphoria" or TI "Dysthymic disorder*" or AB "Dysthymic disorder*"	title:(depress*) OR abstract:(depress*) OR title:(Paraphrenia) OR abstract:(Paraphrenia) OR title:(Involutional Psychos*) OR abstract:(Involutional Psychos*) OR title:(Melancholia) OR abstract:(Melancholia) OR title:(unipolar disorder*) OR abstract:(unipolar disorder*) OR title:(Dysphoria) OR abstract:(Dysphoria) OR title:(Dysthymic Disorder*) OR abstract:(Dysthymic Disorder*)

**Search component 2: exercise**	exp Exercise/ or exp physical activity/ or kinesiotherapy.ti,ab. or exercise.ti,ab. or physical activity.ti,ab. or yoga.ti,ab. or tai chi.ti,ab. or pilates.ti,ab. or walk*.ti,ab. or jogging.ti,ab. or run.ti,ab. or running.ti,ab. or swim*.ti,ab. or danc*.ti,ab. or cycl*.ti,ab. or weight bearing strengthening program*.ti,ab. or exertion.ti,ab. or fitness workout*.ti,ab. or physical conditioning.ti,ab. or physical effort.ti,ab. or physical work out*.ti,ab. or physical workout*.ti,ab. or kinesiotherap*.ti,ab. or rowing.ti,ab. or plyometric drill*.ti,ab. or stretch shortening drill*.ti,ab. or gymnastic*.ti,ab. or squatting.ti,ab. or weight-lifting strengthening program*.ti,ab. or ((exercise or resistance OR strength OR aerobic OR anaerobic OR power OR fitness OR endurance OR plyometric OR continuous OR circuit OR cross OR interval or strength-type) ADJ (fitness* or training* or therap* or exercise* or work-out*)).ti,ab.	exp Exercise/ or exp physical activity/ or exp kinesiotherapy/ or kinesiotherapy.ti,ab. or exercise.ti,ab. or physical activity.ti,ab. or yoga.ti,ab. or tai chi.ti,ab. or pilates.ti,ab. or walk*.ti,ab. or jogging.ti,ab. or run.ti,ab. or running.ti,ab. or swim*.ti,ab. or danc*.ti,ab. or cycl*.ti,ab. or weight bearing strengthening program*.ti,ab. or exertion.ti,ab. or fitness workout*.ti,ab. or physical conditioning.ti,ab. or physical effort.ti,ab. or<br><br>physical work out*.ti,ab. or physical workout*.ti,ab. or kinesiotherap*.ti,ab. or rowing.ti,ab. or plyometric drill*.ti,ab. or stretch shortening drill*.ti,ab. or gymnastic*.ti,ab. or squatting.ti,ab. or weight-lifting strengthening program*.ti,ab.<br><br>or ((exercise or resistance OR strength OR aerobic OR anaerobic OR power OR fitness OR endurance OR plyometric OR continuous OR circuit OR cross OR interval or strength-type) ADJ (fitness* or training* or therap* or exercise* or work-out*)).ti,ab.	exp Exercise/ OR exp Aerobic Exercise/ or kinesiotherapy.ti,ab. or exercise.ti,ab. or physical activity.ti,ab. or yoga.ti,ab. or tai chi.ti,ab. or pilates.ti,ab. or walk*.ti,ab. or jogging.ti,ab. or run.ti,ab. or running.ti,ab. or swim*.ti,ab. or danc*.ti,ab. or cycl*.ti,ab. or weight bearing strengthening program*.ti,ab. or exertion.ti,ab. or fitness workout*.ti,ab. or physical conditioning.ti,ab. or physical effort.ti,ab. or<br><br>physical work out*.ti,ab. or physical workout*.ti,ab. or kinesiotherap*.ti,ab. or rowing.ti,ab. or plyometric drill*.ti,ab. or stretch shortening drill*.ti,ab. or gymnastic*.ti,ab. or squatting.ti,ab. or weight-lifting strengthening program*.ti,ab.<br><br>or ((exercise or resistance OR strength OR aerobic OR anaerobic OR power OR fitness OR endurance OR plyometric OR continuous OR circuit OR cross OR interval or strength-type) ADJ (fitness* or training* or therap* or exercise* or work-out*)).ti,ab.	DE "EXERCISE" OR DE "EXERCISE therapy" OR DE "PHYSICAL activity" OR DE "AEROBIC exercises" OR DE "ANAEROBIC exercises" OR DE "PILATES method" OR DE "RUNNING" OR DE "STRENGTH training*" OR DE "TAI chi" OR DE "PHYSICAL training* & conditioning" OR DE "ANAEROBIC training*" OR DE "BICYCLE racing training*" OR DE "CONTINUOUS training* (Exercise)" OR DE "CYCLING training*" OR DE "ENDURANCE sports training*" OR DE "INTERVAL training*" OR DE "RESISTANCE training*" OR DE "ROWING training*" OR DE "RUNNING training*" OR DE "SWIMMING training*" , DE "WEIGHT training*" OR (TI "exercise" OR AB "exercise") OR (TI "physical activity" OR AB "physical activity") OR (TI "yoga" OR AB "yoga") OR (TI "tai chi" OR AB "tai chi") OR (TI "pilates" OR AB "pilates") OR (TI "walk*" OR AB "walk*") OR (TI "jogging" OR AB "jogging") OR (TI "run" OR AB "run") OR (TI "running" OR AB "running") OR (TI "swim*" OR AB "swim*") OR (TI "danc*" OR AB "danc*") OR (TI "cycl*" OR AB "cycl*") OR (TI "weight bearing strengthening program*" OR AB "weight bearing strengthening program*") OR (TI "exertion"	(title:(exercise) OR abstract:(exercise)) OR (title:(physical activity) OR abstract:(physical activity)) OR (title:(resistance training*) OR abstract:(resistance training*)) OR (title:(strength training*) OR abstract:(strength training*)) OR (title:(aerobic training*) OR abstract:(aerobic training*)) OR (title:(anaerobic training*) OR abstract:(anaerobic training*)) OR (title:(yoga) OR abstract:(yoga)) OR (title:(tai chi) OR abstract:(tai chi)) OR (title:(pilates) OR abstract:(pilates)) OR (title:(jogging) OR abstract:(jogging)) OR (title:(run) OR abstract:(run)) OR (title:(running) OR abstract:(running)) OR (title:(swim*) OR abstract:(swim*)) OR (title:(danc*) OR abstract:(danc*)) OR (title:(cycl*) OR abstract:(cycl*)) OR (title:(weight bearing strengthening program*) OR abstract:(weight bearing strengthening program*)) OR (title:(power training*) OR abstract:(power training*)) OR (title:(exertion) OR abstract:(exertion)) OR (title:(fitness training*) OR abstract:(fitness training*)) OR (title:(fitness workout*) OR abstract:(fitness workout*)) OR (title:(physical conditioning) OR

				OR AB "exertion") OR (TI "fitness workout*" OR AB "fitness workout*") OR (TI "physical conditioning" OR AB "physical conditioning") OR (TI "physical effort" OR AB "physical effort") OR (TI "physical work out*" OR AB "physical work out*") OR (TI "physical workout*" OR AB "physical workout*") OR (TI "kinesiotherap*" OR AB "kinesiotherap*") OR (TI "rowing" OR AB "rowing") OR (TI "plyometric drill*" OR AB "plyometric drill*") OR (TI "stretch shortening drill*" OR AB "stretch shortening drill*") OR (TI "gymnastic*" OR AB "gymnastic*") OR (TI "squatting" OR AB "squatting") OR (TI "weight-lifting strengthening program*" OR AB "weight-lifting strengthening program*") OR (((TI "exercise" OR AB "exercise") OR (TI "resistance" OR AB "resistance") OR (TI "strength" OR AB "strength") OR (TI "aerobic" OR AB "aerobic") OR (TI "anaerobic" OR AB "anaerobic") OR (TI "power" OR AB "power") OR (TI "fitness" OR AB "fitness") OR (TI "endurance" OR AB "endurance") OR (TI "plyometric" OR AB "plyometric") OR (TI "continuous" OR AB "continuous") OR (TI "circuit"	abstract:(physical conditioning)) OR (title:(physical effort) OR abstract:(physical effort)) OR (title:(physical work out*) OR abstract:(physical work out*)) OR (title:(physical workout*) OR abstract:(physical workout*)) OR (title:(kinesiotherap*) OR abstract:(kinesiotherap*)) OR (title:(endurance training*) OR abstract:(endurance training*)) OR (title:(plyometric drill*) OR abstract:(plyometric drill*)) OR (title:(plyometric training*) OR abstract:(plyometric training*)) OR (title:(stretch shortening drill*) OR abstract:(stretch shortening drill*)) OR (title:(continuous training*) OR abstract:(continuous training*)) OR (title:(circuit training*) OR abstract:(circuit training*)) OR (title:(cross training*) OR abstract:(cross training*)) OR (title:(gymnastic*) OR abstract:(gymnastic*)) OR (title:(interval training*) OR abstract:(interval training*)) OR (title:(squatting) OR abstract:(squatting)) OR (title:(weight-lifting strengthening program*) OR abstract:(weight-lifting strengthening program*))

				OR AB "circuit") OR (TI "cross" OR AB "cross") OR (TI "interval" OR AB "interval") OR (TI "strength-type" OR AB "strength-type")) N ((TI "fitness*" OR AB "fitness*") OR (TI "training*" OR AB "training*") OR (TI "therap*" OR AB "therap*") OR (TI "exercise*" OR AB "exercise*") OR (TI "work-out*" OR AB "work-out*")))	

Search component	Search string:<br>MEDLINE via Ovid	Search string:<br>Embase via Ovid	Search string:<br>PsycINFO via Ovid	Search string:<br>SPORTDiscus via EBSCO	Search string:<br>Epistemonikos
**Search component 3: heart rate variability (HRV)**	((heart rate OR heart rhythm OR heart period OR heart beat OR heartbeat OR rr OR cycle length OR cardiac rate OR cardiac rhythm OR cardiac period OR cardiac beat) ADJ3 (variability OR variation OR pattern* OR profile* OR alteration* OR dynamic change* OR control)).ti,ab. or cardiac autonomic nervous system activity.ti,ab. or HRV.ti,ab.	exp heart rate variability/ or ((heart rate OR heart rhythm OR heart period OR heart beat OR heartbeat OR rr OR cycle length OR cardiac rate OR cardiac rhythm OR cardiac period OR cardiac beat) ADJ3 (variability OR variation OR pattern* OR profile* OR alteration* OR dynamic change* OR control)).ti,ab. or cardiac autonomic nervous system activity.ti,ab. or HRV.ti,ab.	exp heart rate variability/ or ((heart rate OR heart rhythm OR heart period OR heart beat OR heartbeat OR rr OR cycle length OR cardiac rate OR cardiac rhythm OR cardiac period OR cardiac beat) ADJ3 (variability OR variation OR pattern* OR profile* OR alteration* OR dynamic change* OR control)).ti,ab. or cardiac autonomic nervous system activity.ti,ab. or HRV.ti,ab.	(((TI "heart rate" OR AB "heart rate") OR (TI "heart rhythm" OR AB "heart rhythm") OR (TI "heart period" OR AB "heart period") OR (TI "heart beat" OR AB "heart beat") OR (TI "heartbeat" OR AB "heartbeat") OR (TI "rr" OR AB "rr") OR (TI "cycle length" OR AB "cycle length") OR (TI "cardiac rate" OR AB "cardiac rate") OR (TI "cardiac rhythm" OR AB "cardiac rhythm") OR (TI "cardiac period" OR AB "cardiac period") OR (TI "cardiac beat" OR AB "cardiac beat")) N3 ((TI "variability" OR AB "variability") OR (TI "variation" OR AB "variation") OR (TI "pattern*" OR AB "pattern*") OR (TI "profile*" OR AB "profile*") OR (TI "alteration*" OR AB "alteration*") OR (TI "dynamic change*" OR AB "dynamic change*") OR (TI "control" OR AB "control"))) OR (TI "cardiac autonomic nervous system activity" OR AB "cardiac autonomic nervous system activity") OR (TI "HRV" OR AB "HRV")	(title:(heart rate variability) OR abstract:(heart rate variability)) OR (title:(HRV) OR abstract:(HRV)) OR (title:(rr variability) OR abstract:(rr variability)) OR (title:(cycle length variability) OR abstract:(cycle length variability)) OR (title:(heart period variability) OR abstract:(heart period variability)) OR (title:(heart rate alteration) OR abstract:(heart rate alteration)) OR (title:(cardiac autonomic nervous system activity) OR abstract:(cardiac autonomic nervous system activity)) OR (title:(heart rate pattern*) OR abstract:(heart rate pattern*)) OR (title:(heart rate profile*) OR abstract:(heart rate profile*)) OR (title:(heartbeat control) OR abstract:(heartbeat control)) OR (title:(heart rhythm control) OR abstract:(heart rhythm control)) OR (title:(cardiac beat variation) OR abstract:(cardiac beat variation)) OR (title:(cardiac beat variability) OR abstract:(cardiac beat variability)) OR (title:(cardiac beat alteration) OR abstract:(cardiac beat alteration)) OR (title:(cardiac rhythm variability) OR abstract:(cardiac rhythm variability))

Search component	Search string:<br>MEDLINE via Ovid	Search string:<br>Embase via Ovid	Search string:<br>PsycINFO via Ovid	Search string:<br>SPORTDiscus via EBSCO	Search string:<br>Epistemonikos
**Combination all three search components (SC)**	(SC 3 HRV AND SC 2 Exercise)<br>OR<br>(SC 3 HRV AND SC 1 DEPRESSION)<br>OR<br>(SC 2 EXERCISE AND SC 1 DEPRESSION)	(SC 3 HRV AND SC 2 Exercise)<br>OR<br>(SC 3 HRV AND SC 1 DEPRESSION)<br>OR<br>(SC 2 EXERCISE AND SC 1 DEPRESSION)	(SC 3 HRV AND SC 2 Exercise)<br>OR<br>(SC 3 HRV AND SC 1 DEPRESSION)<br>OR<br>(SC 2 EXERCISE AND SC 1 DEPRESSION)	(SC 3 HRV AND SC 2 Exercise)<br>OR<br>(SC 3 HRV AND SC 1 DEPRESSION)<br>OR<br>(SC 2 EXERCISE AND SC 1 DEPRESSION)	(SC 3 HRV AND SC 2 Exercise)<br>OR<br>(SC 3 HRV AND SC 1 DEPRESSION)<br>OR<br>(SC 2 EXERCISE AND SC 1 DEPRESSION)
	AND	AND	AND	AND	AND
**Filter systematic review & meta-analysis**	(((comprehensive* or systematic*) adj3 (bibliographic* or review* or literature)) or (meta-analy* or metaanaly* or "research synthesis" or ((information or data) adj3 synthesis) or (data adj2 extract*))).ti,ab. or (cinahl or (cochrane adj3 trial*) or embase or medline or psyclit or (psycinfo not "psycinfo database") or pubmed or scopus or "sociological abstracts" or "web of science").ab. or "cochrane database of systematic reviews".jn. or ((review adj5 (rationale or evidence)).ti,ab. and review.pt.) or meta-analysis as topic/ or Meta-Analysis.pt.	(((comprehensive* or integrative or systematic*) adj3 (bibliographic* or review* or literature)) or (meta-analy* or metaanaly* or "research synthesis" or ((information or data) adj3 synthesis) or (data adj2 extract*))).ti,ab. or (cinahl or (cochrane adj3 trial*) or embase or medline or psyclit or (psycinfo not "psycinfo database") or pubmed or scopus or "sociological abstracts" or "web of science").ab. or ("cochrane database of systematic reviews" or evidence report technology assessment or evidence report technology assessment summary).jn. or Evidence Report: Technology Assessment*.jn. OR systematic review/ OR "systematic review (topic)"/ OR meta analysis/ OR "meta analysis (topic)"/	(((comprehensive* or integrative or systematic*) adj3 (bibliographic* or review* or literature)) or (meta-analy* or metaanaly* or "research synthesis" or ((information or data) adj3 synthesis) or (data adj2 extract*))).ti,ab,id. or ((review adj5 (rationale or evidence)).ti,ab,id. and "Literature Review".md.) or (cinahl or (cochrane adj3 trial*) or embase or medline or psyclit or pubmed or scopus or "sociological abstracts" or "web of science").ab. or ("systematic review" or "meta analysis").md.	(TI (systematic* n3 review*)) or (AB (systematic* n3 review*)) or (TI (systematic* n3 bibliographic*)) or (AB (systematic* n3 bibliographic*)) or (TI (systematic* n3 literature)) or (AB (systematic* n3 literature)) or (TI (comprehensive* n3 literature)) or (AB (comprehensive* n3 literature)) or (TI (comprehensive* n3 bibliographic*)) or (AB (comprehensive* n3 bibliographic*)) or (TI (integrative n3 review)) or (AB (integrative n3 review)) or (JN “Cochrane Database of Systematic Reviews”) or (TI (information n2 synthesis)) or (TI (data n2 synthesis)) or (AB (information n2 synthesis)) or (AB (data n2 synthesis)) or (TI (data n2 extract*)) or (AB (data n2 extract*)) or (AB (medline or pubmed or psyclit or cinahl or (psycinfo not “psycinfo database”) or “web of science”	Filter from Epistemonikos.org Publication type: systematic review

Search component	Search string: MEDLINE via Ovid	Search string: Embase via Ovid	Search string: PsycINFO via Ovid	Search string: SPORTDiscus via EBSCO	Search string: Epistemonikos
				or scopus or embase) or (DE "SYSTEMATIC reviews (Medical research)") or (DE "META-analysis") or (TI (meta-analy* or metaanaly*)) or (AB (meta-analy* or metaanaly*))	
	AND	AND	AND	AND	AND
**Filter adults**	not ((exp infant/ or exp child/ or adolescent/) not (exp adult/))	*NOT (exp juvenile/ NOT exp adult/)*			

S3 – Supplement 3 Excluded Studies

Reference	Titel	Exclusion reason
Abdullah et al. 2021	Physical activity and depression during pregnancy: A scoping review	Population
Abera et al. 2022	Effect of relaxation interventions in pregnant women on maternal and neonatal outcomes: A systematic review and meta-analysis	Population
Abreu et al. 2018	Can high-intensity interval training change cardiac autonomic control? A systematic review	Population
Ahmed et al. 2022	Physiological monitoring of stress and major depression: A review of the current monitoring techniques and considerations for the future	Population
Ahn et al. 2023	Effects of exercise therapy on global cognitive function and, depression in older adults with mild cognitive impairment: A systematic review and meta-analysis	Population
Ai et al. 2023	Research trends of exercise therapy of college students in depression from 2002 to 2022: a bibliometric analysis	Outcome
Al-Harbi et al. 2012	Treatment-resistant depression: Therapeutic trends, challenges, and future directions	Study design
Almagro Valverde et al. 2014	Physical activity and depression: a systematic review	Language
Almeida et al. 2022	Isometric handgrip exercise training reduces resting systolic blood pressure but does not interfere with diastolic blood pressure and heart rate variability in hypertensive subjects: a systematic review and meta-analysis of randomized clinical trials	Population
Al-Qahtani et al. 2018	Exercise as a treatment modality for depression: A narrative review	Study design
Alves et al. 2023	Water-based exercise effects on cardiac autonomic response: A systematic review	Population
Amekran et al. 2023	Effects of aerobic training on heart rate variability in healthy adults: a systematic review	Missing Full text
Amro et al. 2021	Effects of increasing aerobic capacity on improving psychological problems seen in patients with COVID-19: A review	Population
Andersson et al. 2015	Physical activity is just as good as CBT or drugs for depression	Language
Andersson et al. 2022	Physical activity has an acute effect on depression and anxiety syndromes	Study design
Anthony et al. 2008	Relaxation for depression	Intervention
António et al. 2015	Cardiac autonomic modulation adjustments in isometric exercise	Population
Archer et al. 2014	Effects of physical exercise on depressive symptoms and biomarkers in depression	Study design

Arent et al. 2001	Mental health and physical activity. The effects of physical activity on selected mental health variables: determining causation	Study design
Argyropoulos et al. 2021	Combined exercise programs as protective factor against depression later in life: A systematic review	Population
Ashdown-Franks et al. 2018	Is it possible for people with severe mental illness to sit less and move more? A systematic review of interventions to increase physical activity or reduce sedentary behaviour	Outcome
Ashdown-Franks et al. 2019	The evidence for physical activity in the management of major mental illnesses: a concise overview to inform busy clinicians' practice and guide policy	Study design
Ashdown-Franks et al. 2020	Exercise as Medicine for Mental and Substance Use Disorders: A Meta-review of the Benefits for Neuropsychiatric and Cognitive Outcomes	Study design
Askar et al. 2021	Effective Intervention in Reducing Depression in General Population during Pandemic of Covid-19	Study design
Augustin et al. 2023	Resistance Training in Depression	Study design
Aujla-Sidhu 2022	Treatments for depression in south Asian adults: A systematic review	Study design
Azar et al. 2008	The association between physical activity and depressive symptoms in young women: A review	Population
Barbour et al. 2005	Exercise training and depression in older adults	Study design
Barbui et al. 2009	Depression in adults: drug and physical treatments	Study design
Barnish et al. 2023	Group-based active artistic interventions for adults with primary anxiety and depression: a systematic review	Intervention
Barrenetxea-Garcia et al. 2022	Recovery in water polo: how much do we have to know? A systematic review	Intervention
Baskar et al. 2022	Interventions For Postpartum Depression: Systematic Review	Outcome
Bassett et al. 2016	A literature review of heart rate variability in depressive and bipolar disorders	Study design
Batacan et al. 2017	Effects of high-intensity interval training on cardiometabolic health: a systematic review and meta-analysis of intervention studies	Population
Bates et al. 2021	Effects of Acute Prolonged Sitting and Interrupting Prolonged Sitting on Heart Rate Variability and Heart Rate in Adults: A Meta-Analysis	Intervention
Bauer et al. 2008	Sport therapy for depressive disorders: systematic review	Study design
Beland et al. 2019	Aerobic exercise alleviates depressive symptoms in patients with a major non-communicable chronic disease: a systematic review and meta-analysis	Population
Bell et al. 2013	Psycho-physiological markers of overreaching and overtraining in endurance sports: A review of the evidence	Study design
Bellenger et al. 2016	Monitoring Athletic Training Status Through Autonomic Heart Rate Regulation: A Systematic Review and Meta-Analysis	Outcome
Bellon et al. 2021	Effectiveness of exercise-based interventions in reducing depressive symptoms in people without clinical depression: systematic review and meta-analysis of randomised controlled trials	Population

Belvederi Murri et al. 2020	Physical Activity Promotes Health and Reduces Cardiovascular Mortality in Depressed Populations: A Literature Overview	Study design
Bernard et al. 2015	A commentary on the importance of controlling for medication use within trials on the effects of exercise on depression and anxiety	Study design
Bhargav et al. 2021	Yoga and psychiatric disorders: A review of biomarker evidence	Study design
Bhatia et al. 2022	Outdoor Community Ambulation Interventions to Improve Physical and Mental Health in Older Adults: A Systematic Review and Meta-Analysis	Population
Biddle et al. 1995	Exercise and psychosocial health	Study design
Bigarella et al. 2021	Exercise for depression and depressive symptoms in older adults: An umbrella review of systematic reviews and meta-analyses	Study design
Blanck et al. 2018	Effects of mindfulness exercises as stand-alone intervention on symptoms of anxiety and depression: Systematic review and meta-analysis	Intervention
Blase et al. 2021	Neurophysiological approach by self-control of your stress-related autonomic nervous system with depression, stress and anxiety patients	Intervention
Blase et al. 2021	Neurophysiological Approach by Self-Control of Your Stress-Related Autonomic Nervous System with Depression, Stress and Anxiety Patients	Outcome
Blumenthal et al. 2023	Exercise as a therapeutic modality for the prevention and treatment of depression	Study design
Blumenthal et al. 2023	Exercise as a therapeutic modality for the prevention and treatment of depression	Study design
Boix-Vilella et al. 2017	Evidence of Pilates practice on the mental health of healthy people	Language
Bond et al. 2020	Do exercise trials for adults with depression account for comorbid anxiety? A systematic review	Outcome
Bond et al. 2021	Erratum "Do exercise trials for adults with depression account for comorbid anxiety? A systematic review"	Study design
Bortveit et al. 2022	Guided Internet-Delivered Treatment for Depression: Scoping Review	Intervention
Bouchard et al. 2001	Individual differences in response to regular physical activity	Study design
Bourbeau et al. 2020	The Combined Effect of Exercise and Behavioral Therapy for Depression and Anxiety: Systematic Review and Meta-Analysis	Intervention
Bourke et al. 2022	The acute affective response to physical activity in people with depression: A meta-analysis	Outcome
Bradley et al. 2022	Systematic review of lifestyle interventions to improve weight, physical activity and diet among people with a mental health condition	Intervention
Breedvelt et al. 2019	The effects of meditation, yoga, and mindfulness on depression, anxiety, and stress in tertiary education students: A meta-analysis	Intervention
Bressington et al. 2018	The effects of group-based Laughter Yoga interventions on mental health in adults: A systematic review	Population

Bricca et al. 2020	Benefits and harms of exercise therapy in people with multimorbidity: A systematic review and meta-analysis of randomised controlled trials	Population
Bridges et al. 2017	The Efficacy of Yoga as a Form of Treatment for Depression	Population
Brinsley et al. 2020	Effects of yoga on depressive symptoms in people with mental disorders: a systematic review and meta-analysis	Population
Brinsley et al. 2021	Effects of yoga on depressive symptoms in people with mental disorders: a systematic review and meta-analysis	Population
Brito et al. 2011	Resistance exercise: a review about your hemodynamics and autonomics aspects	Language
Brondino et al. 2017	A systematic review of cognitive effects of exercise in depression	Outcome
Brown et al. 2017	Does aerobic exercise reduce postpartum depressive symptoms? A systematic review and meta-analysis	Population
Brupbacher et al. 2019	The effects of aerobic, resistance, and meditative movement exercise on sleep in individuals with depression: protocol for a systematic review and network meta-analysis	Study design
Brupbacher et al. 2021	The effects of exercise on sleep in unipolar depression: A systematic review and network meta-analysis	Outcome
Brupbacher et al. 2021	Reply to Hertenstein et al.'s commentary on Brupbacher et al.: The effects of exercise on sleep in unipolar depression: A systematic review and network meta-analysis	Study design
Buechner et al. 2023	Community interventions for anxiety and depression in adults and young people: A systematic review	Population
Burlacu et al. 2021	Influencing cardiovascular outcomes through heart rate variability modulation: A systematic review	Intervention
Bustamante-Sanchez et al. 2020	Effect of Stress on Autonomic and Cardiovascular Systems in Military Population: A Systematic Review	Population
Bustamante-Sánchez et al. 2020	Physical activity and depression in young women	Population
Butterfield et al. 2017	Yoga and mindfulness for anxiety and depression and the role of mental health professionals: a literature review	Study design
Cabral et al. 2011	Effectiveness of yoga therapy as a complementary treatment for major psychiatric disorders: a meta-analysis	Outcome
Cai et al. 2022	Physical activity before and during pregnancy and maternal mental health: A systematic review and meta-analysis of observational studies	Population
Callaghan et al. 2004	Exercise: A neglected intervention in mental health care	Study design
Calleja-Romero et al. 2021	Acute effects of long-distance races on heart rate variability and arterial stiffness: A systematic review and meta-analysis	Outcome
Cardoso et al. 2022	Heart rate pro ile and heart rate variability in volleyball athletes: a systematic review with meta-analyses	Comparator
Carneiro et al. 2018	Portuguese and Brazilian guidelines for the treatment of depression: Exercise as medicine	Study design

Carneiro et al. 2022	Web-based exercise interventions for patients with depressive and anxiety disorders: a systematic review of randomized controlled trials	Population
Carney et al. 2002	Depression as a risk factor for cardiac mortality and morbidity: a review of potential mechanisms	Study design
Carney et al. 2005	Depression, the Autonomic Nervous System, and Coronary Heart Disease	Study design
Carraca et al. 2021	Effect of exercise training on psychological outcomes in adults with overweight or obesity: A systematic review and meta-analysis	Population
Carraca et al. 2021	Effect of exercise training on psychological outcomes in adults with overweight or obesity: A systematic review and meta-analysis	Population
Carroll et al. 2022	An Evidence-Based Theory of Psychological Adjustment to Long-Term Physical Health Conditions: Applications in Clinical Practice	Intervention
Castaldo et al. 2015	Acute mental stress assessment via short term HRV analysis in healthy adults: A systematic review with meta-analysis	Population
Catalan-Matamoros et al. 2016	Exercise improves depressive symptoms in older adults: An umbrella review of systematic reviews and meta-analyses	Study design
Cerezuela et al. 2023	Pickleball and mental health in adults: A systematic review	Population
Cezaretto et al. 2015	Interdisciplinary approach in response to lifestyle interventions for reducing cardiometabolic risk and the influence of depression	Population
Chang et al. 2019	Physical and Psychological Health Outcomes of Qigong Exercise in Older Adults: A Systematic Review and Meta-Analysis	Population
Chapman et al. 2012	Characteristics of physical activity interventions for adults with mental illness: A review	Outcome
Charron et al. 2021	Unclear outcomes of heart rate variability following a concussion: A systematic review	Population
Chauntry et al. 2022	Sedentary behaviour, physical activity and psychobiological stress reactivity: A systematic review	Intervention
Chekroud et al. 2018	Efficacy of resistance exercise training with depressive symptoms	Study design
Chen et al. 2019	Pharmacological and non-pharmacological treatments for major depressive disorder in adults: A systematic review and network meta-analysis	Outcome
Chen et al. 2020	Training variables and autonomic nervous system adaption	Language
Chen et al. 2020	Exercise Intervention in Treatment of Neuropsychological Diseases: A Review	Study design
Chen et al. 2021	Non-pharmacological interventions for older adults with depressive symptoms: A network meta-analysis of 35 randomized controlled trials	Duplicate
Chen et al. 2021	Effects of Tai Chi Chuan on the Physical and Mental Health of the Elderly: A Systematic Review	Population
Chen et al. 2021	Effects of Tai Chi Chuan on the Physical and Mental Health of the Elderly: A Systematic Review	Population

Chen et al. 2022	Intervention methods for improving reduced heart rate variability in patients with major depressive disorder: A systematic review and meta-analysis	Comparator
Chen et al. 2022	Types of exercise and training duration on depressive symptoms among older adults in long-term care facilities	Population
Chen et al. 2024	Effects of internet-based exercise intervention on depression and anxiety: A systematic review and meta-analysis	Population
Cherubal et al. 2019	Physical activity and mental health in India: A narrative review	Population
Chesnut et al. 2021	Stress Markers for Mental States and Biotypes of Depression and Anxiety: A Scoping Review and Preliminary Illustrative Analysis	Study design
Chi et al., year unknown	Tai Chi for depression	Duplicate
Chobe et al. 2020	Impact of Yoga on cognition and mental health among elderly: A systematic review	Duplicate
Choi et al. 2019	Assessment of bidirectional relationships between physical activity and depression among adults: A 2-sample mendelian randomization study	Study design
Choi et al. 2022	A Systemic Review of depressive intervention for the older adults	Intervention
Chow et al. 2021	Appraisal of systematic reviews on interventions for postpartum depression: systematic review	Study design
Christian 2022	Meta-analysis of the effectiveness of biological and non-biological treatments for postpartum depression	Intervention
Chu et al. 2014	Do workplace physical activity interventions improve mental health outcomes?	Population
Ciezar-Andersen et al. 2021	A systematic review of yoga interventions for helping health professionals and students	Population
Cimpianu et al. 2017	Vagus nerve stimulation in psychiatry: A systematic review of the available evidence	Intervention
Cipriani et al. 2011	Depression in adults: drug and physical treatments	Study design
Cipriano et al. 2019	Current insights of inspiratory muscle training on the cardiovascular system: a systematic review with meta-analysis	Population
Cole et al. 2016	Effects of Tai Chi exercise on heart rate variability	Study design
Colquhoun et al. 2013	Exercise now on a par with drug therapy for treatment of patients with coronary heart disease and depression?	Population
Conn et al. 2010	Depressive symptom outcomes of physical activity interventions: meta-analysis findings	Population
Correia et al. 2023	Associations between sleep-related heart rate variability and both sleep and symptoms of depression and anxiety: A systematic review	Outcome
Costa et al. 2018	Effects of weight changes in the autonomic nervous system: A systematic review and meta-analysis	Intervention
Coventry et al. 2021	Nature-based outdoor activities for mental and physical health: Systematic review and meta-analysis	Population

Cox et al. 2007	Is exercise an evidence-based intervention for clinical depression in older adults: A meta-analysis of randomized studies 2000-2006	Study design
Craft et al. 2004	The Benefits of Exercise for the Clinically Depressed	Study design
Crawford-Faucher et al. 2013	Exercise reduces depressive symptoms in patients with chronic illness	Population
Crumbie et al. 2015	The Impact of Dance Interventions on Mood and Depression in Older Adults	Study design
Cugusi et al. 2017	Nordic walking and cardiovascular disease prevention: A systematic review of randomized controlled trials	Population
Cuijpers et al. 2014	The effects of psychological treatments for adult depression on physical activity: A systematic review	Intervention
Cutler et al. 2023	Treatment of Mood and Depressive Disorders With Complementary and Alternative Medicine: Efficacy Review	Study design
da Cunha et al. 2023	Effects of exercise training on inflammatory, neurotrophic and immunological markers and neurotransmitters in people with depression: A systematic review and meta-analysis	Outcome
da Silva et al. 2015	Heart rate variability indexes as a marker of chronic adaptation in athletes: a systematic review	Outcome
Da Silva et al. 2022	Effects of Chronic Physical Exercise or Multicomponent Exercise Programs on the Mental Health and Cognition of Older Adults Living in a Nursing Home: A Systematic Review of Studies From the Past 10 Years	Population
Daglas-Georgiou et al. 2021	Treatments for objective and subjective cognitive functioning in young people with depression: Systematic review of current evidence	Outcome
Daley 2008	Exercise and depression: a review of reviews	Study design
Daley et al. 2007	The Role of Exercise in Treating Postpartum Depression: A Review of the Literature	Study design
Daley et al. 2009	The effectiveness of exercise in the management of post-natal depression: systematic review and meta-analysis	Population
Daley et al. 2012	Exercise to treat depression	Study design
Daley et al. 2015	The effectiveness of exercise for the prevention and treatment of antenatal depression: systematic review with meta-analysis	Population
Davis et al. 2012	Mindfulness-Based Treatment for People With Severe Mental Illness: A Literature Review	Study design
de Abreu et al. 2017	Effects of inspiratory muscle training on cardiovascular autonomic control: A systematic review	Population
De Abreu et al. 2018	Effects of high-intensity interval training on cardiac autonomic control: A systematic review of randomized clinical trials	Duplicate
De et al. 2021	Yoga can Alter the Autonomic Neural Activity: A Critical Analysis	Study design
De Manincor 2018	Evidence for yoga as a treatment intervention for depression, anxiety and PTSD - Navigating heterogeneity	Study design
De Rosa et al. 2017	Improving physical health of patients with severe mental disorders: a critical review of lifestyle psychosocial interventions	Outcome

De Sousa et al. 2021	Molecular mechanisms of physical exercise on depression in the elderly: a systematic review	Population
Declercq et al. 2024	A bayesian network meta-analysis to explore modifying factors in randomized controlled trials: what works for whom to reduce depression in nursing home residents?	Population
Dergisi'nden, 2015	Aerobic exercise in depression and anxiety disorders	Study design
Dietz et al. 2016	The influence of physical activity during pregnancy on maternal, fetal or infant heart rate variability: a systematic review	Intervention
Dinas et al. 2010	Effects of exercise and physical activity on depression	Study design
Dingle et al. 2020	The effects of social group interventions for depression: Systematic review	Population
Dingle et al. 2021	The effects of social group interventions for depression: Systematic review	Population
Dirmaier et al. 2009	Evidence-based treatments in the rehabilitation of patients with depression-a literature review	Study design
Dirmaier et al. 2012	Non-pharmacological treatment of depressive disorders: a review of evidence-based treatment options	Study design
Donaghy et al. 2007	Exercise can seriously improve your mental health: Fact or fiction	Study design
Dong et al. 2008	Exercise for older depressed people	Study design
Dong et al. 2024	The effects of mind-body exercise on anxiety and depression in older adults: a systematic review and network meta-analysis	Population
Donnelly et al. 2023	A meta-analysis investigating the outcomes and correlation between heart rate variability biofeedback training on depressive symptoms and heart rate variability outcomes versus standard treatment in comorbid adult populations	Intervention
Drazich et al. 2020	Exergames and Depressive Symptoms in Older Adults: A Systematic Review	Population
Drew et al. 2019	Mental health outcomes of male-only lifestyle behaviour change interventions: A systematic review and meta-analysis	Population
Duan-Porter et al. 2015	Mapping the Evidence: Sex Effects in High-Impact Conditions for Women Veterans - Depression, Diabetes, and Chronic Pain	Study design
Duan-Porter et al. 2016	Evidence Map of Yoga for Depression, Anxiety, and Posttraumatic Stress Disorder	Study design
Duking et al. 2021	Predefined vs data-guided training prescription based on autonomic nervous system variation: A systematic review	Outcome
Dunn et al. 2001	Physical activity dose-response effects on outcomes of depression and anxiety	Duplicate
Dunn et al. 2001	Physical activity dose-response effects on outcomes of depression and anxiety	Duplicate
Dunn et al. 2001	Physical activity dose-response effects on outcomes of depression and anxiety	Study design
Easwaran et al. 2020	Effectiveness of Tai Chi for health promotion for adults with health conditions: a scoping review of Meta-analyses	Study design

Edwards et al. 2022	Mechanisms for blood pressure reduction following isometric exercise training: a systematic review and meta-analysis	Population
Efendi et al. 2023	Effects of chair-based resistance band exercise on physical functioning, sleep quality, and depression of older adults in long-term care facilities: Systematic review and meta-analysis	Population
Eisenberg et al. 2007	Comparative Effectiveness Review Summary Guides for Clinicians	Study design
Ekholm et al. 1996	Autonomic cardiovascular control in pregnancy	Study design
El-Malahi et al. 2024	Beneficial impacts of physical activity on heart rate variability: A systematic review and meta-analysis	Population
Ernst et al. 1998	Complementary therapies for depression: an overview	Duplicate
Ernst et al. 1998	Complementary therapies for depression: An overview	Study design
Espinoza et al. 2022	Community-based interventions for mental health in older adults: A systematic review and meta-analysis of randomized clinical trials	Population
Ettore 2023	Digital Phenotyping for Differential Diagnosis of Major Depressive Episode: Narrative Review	Study design
Eustis et al. 2019	Innovations in the Treatment of Perinatal Depression: the Role of Yoga and Physical Activity Interventions During Pregnancy and Postpartum	Study design
Eyre et al. 2011	Neurobiological effects of exercise on stress-induced depression	Outcome
Eyre et al. 2013	Treating depression and depression-like behavior with physical activity: An immune perspective	Study design
Farah et al. 2016	Non-pharmacological treatment of depression: a systematic review and evidence map	Study design
Farah et al. 2020	Effects of isometric handgrip training on cardiac autonomic profile: A systematic review and meta-analysis study	Population
Farioli-Vecchioli et al. 2017	The role of physical exercise and omega-3 fatty acids in depressive illness in the elderly	Study design
Farris et al. 2020	Mental health benefits from lifestyle physical activity interventions: A systematic review	Population
Farzad et al. 2021	A Scoping Review of the Evidence regarding Assessment and Management of Psychological Features of Shoulder Pain	Population
Fasching et al. 2021	Pooled analysis of patient (pt)-reported outcomes (PROs) in the MONALEESA (ML)-2, -3, and -7 trials: Additional results and key subgroup findings	Population
Feldman et al. 2016	Physical characteristics in mental illness population - literature review	Study design
Felez-Nobrega et al. 2021	Light-intensity physical activity and mental ill health: a systematic review of observational studies in the general population	Population
Felix et al. 2018	The effect of exercise on symptoms of depression in adults diagnosed with clinical depression: an integrative literature review	Study design

Fenton et al. 2018	The impacts of recreation programs on the mental health of postsecondary students in north america: An integrative review	Population
Fernandes et al. 2022	Effects of antidepressant drug therapy with or without physical exercise on inflammatory biomarkers in major depressive disorder: a systematic review and meta-analysis of randomized controlled trials	Outcome
Fernández-Alvarez et al. 2021	Efficacy of bio- and neurofeedback for depression: a meta-analysis	Intervention
Fiani et al. 2023	A Meta-Analysis of the Differential Effects of Antidepressants on the Autonomic Nervous System	Intervention
Field 2016	Yoga research review	Study design
Firth et al. 2020	A meta-review of "lifestyle psychiatry": the role of exercise, smoking, diet and sleep in the prevention and treatment of mental disorders	Study design
Fleming et al. 2018	The effects of pilates on mental health outcomes: A meta-analysis of controlled trials	Population
Flores et al. 2023	Heart rate variability behavior in athletes after a sports concussion: A systematic review	Intervention
Fournie et al. 2021	Heart rate variability biofeedback in chronic disease management: A systematic review	Duplicate
Fournie et al. 2021	Heart rate variability biofeedback in chronic disease management: A systematic review	Intervention
Fournie et al. 2021	Heart rate variability biofeedback in chronic disease management: A systematic review	Population
Fox et al. 1999	The influence of physical activity on mental well-being	Study design
Fox et al. 2000	Physical activity and mental health promotion: The natural partnership	Study design
Franchini et al. 2014	Physiological responses during taekwondo training and competition	Study design
Freeman et al. 2009	Complementary and alternative medicine for perinatal depression	Study design
Freeman et al. 2009	Complementary and Alternative Medicine (CAM): considerations for the treatment of major depressive disorder	Study design
Freeman et al. 2009	Complementary and alternative medicine in major depressive disorder: the American Psychiatric Association Task Force report	Study design
Fruhauf et al. 2018	Current state of the scientific literature on effects of therapeutic climbing on mental health - conclusion: a lot to do	Duplicate
Fruhauf et al. 2018	Current state of the scientific literature on effects of therapeutic climbing on mental health - conclusion: a lot to do.	Population
Furlan et al. 2011	Systematic Review of Intervention Practices for Depression in the Workplace	Outcome
Galante et al. 2021	Mindfulness-based programmes for mental health promotion in adults in nonclinical settings: A systematic review and meta-analysis of randomised controlled trials	Population
Galante et al. 2021	Mindfulness-based programmes for mental health promotion in adults in nonclinical settings: A systematic review and meta-analysis of randomised controlled trials	Population

Gangadhar 2023	Evidence-based integration of yoga in psychiatric practice	Study design
Gartlehner et al. 2015	Nonpharmacological Versus Pharmacological Treatments for Adult Patients With Major Depressive Disorder	Outcome
Gartlehner et al. 2016	Comparative Benefits and Harms of Antidepressant, Psychological, Complementary, and Exercise Treatments for Major Depression: An Evidence Report for a Clinical Practice Guideline From the American College of Physicians	Comparator
Gartlehner et al. 2016	Comparative Benefits and Harms of Antidepressant, Psychological, Complementary, and Exercise Treatments for Major Depression: An Evidence Report for a Clinical Practice Guideline From the American College of Physicians	Duplicate
Georgiou et al. 2018	Can Wearable Devices Accurately Measure Heart Rate Variability? A Systematic Review	Outcome
Getu et al. 2020	Home Based Physical Exercise for Prevention and Management of COVID-19 Pandemic and for Its Associated Consequences	Population
Gianfredi et al. 2020	Depression and Objectively Measured Physical Activity: A Systematic Review and Meta-Analysis	Population
Gill et al. 2010	Clinical Inquiries: Does exercise alleviate symptoms of depression?	Study design
Gill et al. 2020	Non-pharmacological depression therapies for older Chinese adults: A systematic review & meta-analysis	Intervention
Glowacki et al. 2017	Barriers and facilitators to physical activity and exercise among adults with depression: A scoping review	Outcome
Gogulla et al. 2012	Effects of physical activity and physical training on the psychological status of older persons with and without cognitive impairment	Study design
Gómez-Gómez et al. 2020	Effectiveness of universal multiple-risk lifestyle interventions in reducing depressive symptoms: Systematic review and meta-analysis	Population
Goncalves et al. 2020	Problems of scientific methodology related to placebo control in Qigong studies: A systematic review	Population
Gong et al. 2015	Yoga for prenatal depression: a systematic review and meta-analysis	Population
Gordon et al. 2018	The Effects of Resistance Exercise Training on Depressive Symptoms: A Meta-Analysis of Randomized Controlled Trials	Duplicate
Gordon et al. 2018	Association of Efficacy of Resistance Exercise Training With Depressive Symptoms: Meta-analysis and Meta-regression Analysis of Randomized Clinical Trials	Population
Goyal et al. 2014	Meditation programs for psychological stress and well-being: a systematic review and meta-analysis	Population
Granero-Gallegos et al. 2020	Hrv-based training for improving vo2max in endurance athletes. A systematic review with meta-analysis	Outcome
Grassini 2022	A Systematic Review and Meta-Analysis of Nature Walk as an Intervention for Anxiety and Depression	Population
Grassler et al. 2021	Effects of Different Training Interventions on Heart Rate Variability and Cardiovascular Health and Risk Factors in Young and Middle-Aged Adults: A Systematic Review	Duplicate

Grassler et al. 2021	Effects of different exercise interventions on cardiac autonomic control and secondary health factors in middle-aged adults: A systematic review	Duplicate
Griffin et al. 2014	The effectiveness of exercise for the prevention and treatment of antenatal depression: systematic review with meta-analysis	Duplicate
Gronwald et al. 2019	Correlation properties of heart rate variability during endurance exercise: A systematic review	Outcome
Gujral et al. 2017	Exercise effects on depression: Possible neural mechanisms	Outcome
Gultyaeva et al. 2019	Exercise for depression treatment. Physiological mechanisms	Language
Gumarães et al. 2006	The influence of exercise on depressive disorders of the elderly: a systematic review	Language
Guszkowska et al. 2004	Effects of exercise on anxiety, depression and mood	Language
Haaranen et al. 2014	Digital and video games in health promotion: Systematic review of games and health behavior	Population
Haas 2016	Yoga for Prenatal Depression	Study design
Haller et al. 2019	Complementary therapies for depression: A systematic review of reviews	Study design
Hallgren et al. 2016	Exercise, physical activity, and sedentary behavior in the treatment of depression: Broadening the scientific perspectives and clinical opportunities	Study design
Hamilton et al. 2016	Atypical reactivity of heart rate variability to stress and depression across development: Systematic review of the literature and directions for future research	Population
Harris et al. 2024	Meta-Analysis: Exercise as Effective as Therapy for Treating Depression	Study design
Hausenblas et al. 2001	Psychological effects of exercise with general and diseased populations	Study design
Hawes et al. 2020	Ideal cardiovascular health in racially and ethnically diverse people with serious mental illness	Population
He et al. 2023	The impact of physical activity intervention on perinatal depression: A systematic review and meta-analysis	Population
Heinzel et al. 2020	Antidepressive effects of physical exercise interventions. Review of potential mechanisms of action	Study design
Heisel et al. 2020	Sports therapy as adjuvant or alternative treatment for depression	Study design
Heiss et al. 2020	Heart rate variability as a biobehavioral marker of diverse psychopathologies: A review and argument for an "ideal range"	Population
Heiss et al. 2021	Heart rate variability as a biobehavioral marker of diverse psychopathologies: A review and argument for an "ideal range"	Study design
Heissel et al. 2023	Exercise as medicine for depressive symptoms? A systematic review and meta-analysis with meta-regression	Duplicate
Hendriks 2018	The effects of Sahaja Yoga meditation on mental health: a systematic review	Population
Hess et al. 2019	Evidence-based practice: A comparison of International Clinical Practice Guidelines and current research on physical activity for mild to moderate depression	Outcome

Honkalampi et al. 2021	Heart rate variability and chronotype - a systematic review	Intervention
Hooper et al. 2022	Comparative Efficacy of Exercise Training and Conventional Psychotherapies for Adult Depression: A Network Meta-Analysis	Population
Hu et al. 2019	The association of depression and anxiety with cardiac autonomic activity: The role of confounding effects of antidepressants	Study design
Hu et al., year unknown	A Meta-Meta-Analysis of the Effect of Physical Exercise as a Treatment on Depression	Language
Huang et al. 2005	Resting heart rate changes after endurance training in older adults: A meta-analysis	Outcome
Huang et al. 2018	Interventions for common mental health problems among university and college students: A systematic review and meta-analysis of randomized controlled trials	Intervention
Huang et al. 2022	Exergame-based exercise training for depressive symptoms in adults: A systematic review and meta-analysis	Population
Huston et al. 2016	Health benefits of tai chi: What is the evidence?	Study design
Imboden et al. 2020	Exercise as a Treatment for Postnatal Depression	Study design
Iseger et al. 2019	A frontal-vagal network theory for Major Depressive Disorder: Implications for optimizing neuromodulation techniques	Study design
Jarbou et al. 2022	Exercise and yoga during pregnancy and their impact on depression: a systematic literature review	Population
Jarczok et al. 2015	Circadian variation of cardiac autonomic modulation in a rigorously healthy working population: Results from the MICS-cohort	Study design
Jazayeri et al. 2022	Comparison Study of the Effects of Resistance and Endurance Exercise Interventions for the Treatment of Depression: A Meta-Analytical Review	Population
Jiang et al. 2015	Effects of yoga intervention during pregnancy: a review for current status	Population
Jiang et al. 2021	Comparative Efficacy of Multiple Therapies for the Treatment of Patients With Subthreshold Depression: A Systematic Review and Network Meta-Analysis	Intervention
Jiang et al. 2022	Efficacy of nondrug interventions in perinatal depression: A meta-analysis	Population
Jimenez et al. 2017	Effect of Heart Rate Variability Biofeedback on Sport Performance, a Systematic Review	Intervention
Jimenez et al. 2018	Dance movement therapy with older people with a psychiatric condition: A systematic review	Population
John et al. 2015	Nonpharmacological Versus Pharmacological Treatment for Patients With Major Depressive Disorder: Current State of the Evidence	Study design
Jonasson 2019	Using Intensive Longitudinal Data to Study Treatment Effects in Patients with Major Depression: A Systematic Review	Intervention
Jose et al. 2023	Predictors of Heart Rate Variability: A Systematic Review	Population
Joyce et al. 2016	Workplace interventions for common mental disorders: A systematic meta-review	Study design

Ju et al. 2023	The impact of Pilates exercise for depression symptoms in female patients: A systematic review and meta-analysis	Population
Kandola et al. 2019	Physical activity and depression: Towards understanding the antidepressant mechanisms of physical activity	Study design
Kang et al. 2022	Effects of Forest-Based Interventions on Mental Health: A Meta-Analysis of Randomized Controlled Trials	Population
Kemp et al. 2011	Is heart rate variability reduced in depression without cardiovascular disease?	Study design
KhodadadKashi et al. 2023	A Systematic Review and Meta-Analysis of Resistance Training on Quality of Life, Depression, Muscle Strength, and Functional Exercise Capacity in Older Adults Aged 60 Years or More	Population
Kiepe et al. 2010	Systematic review of the effects of dance therapy on mental and physical illnesses	Duplicate
Kiepe et al. 2012	Effects of dance therapy and ballroom dances on physical and mental illnesses: A systematic review	Population
Kim et al. 2018	Stress and Heart Rate Variability: A Meta-Analysis and Review of the Literature	Population
Kiyomi et al. 2013	Behavioural therapies versus other psychological therapies for depression	Intervention
Kjellman 2005	Indications that physical activity has a therapeutic effect in depression. Exercise a valuable complement to current antidepressive therapy	Language
Klainin-Yobas et al. 2015	Effects of relaxation interventions on depression and anxiety among older adults: a systematic review	Population
Klil-Drori et al. 2020	Exercise Intervention for Late-Life Depression: A Meta-Analysis	Population
Knapen et al. 2015	Exercise therapy improves both mental and physical health in patients with major depression	Study design
Koch et al. 2014	Effects of dance movement therapy and dance on health-related psychological outcomes. A meta-analysis update	Population
Koenig et al. 2014	Heart Rate Variability and Swimming	Population
Kolomanska et al. 2019	Physical Activity and Depressive Disorders in Pregnant Women-A Systematic Review	Population
Kolomanska-Bogucka et al. 2019	Physical Activity and the Occurrence of Postnatal Depression-A Systematic Review	Population
Koppelmans et al. 2019	Mechanisms Underlying Exercise as a Treatment for Depression	Study design
Korman et al. 2019	High Intensity Interval training (HIIT) for people with severe mental illness: A systematic review & meta-analysis of intervention studies- considering diverse approaches for mental and physical recovery	Population
Krogh et al. 2017	Exercise for patients with major depression: a systematic review with meta-analysis and trial sequential analysis	Duplicate
Krogh et al. 2017	The effect of exercise in clinically depressed adults: systematic review and meta-analysis of randomized controlled trials	Duplicate
Kuang et al. 2024	The effects of different types of Tai Chi exercise on anxiety and depression in older adults: a systematic review and network meta-analysis	Population

Lagerveld et al. 2010	Factors Associated with Work Participation and Work Functioning in Depressed Workers: A Systematic Review	Intervention
Ledochowski et al. 2016	Physical activity as therapeutic intervention for depression	Population
Ledwidge et al. 1980	Run for your mind - aerobic exercise as a means of alleviating anxiety and depression	Study design
Lee et al. 2022	The Effectiveness of Physical Activity Interventions on Depression in Korea: A Systematic Review and Meta-Analysis	Population
Legnani et al. 2024	Is Pilates effective in improving depressive disorders? A comprehensive overview	Population
Lehrer et al. 2020	Heart Rate Variability Biofeedback Improves Emotional and Physical Health and Performance: A Systematic Review and Meta Analysis	Intervention
Leung et al. 2022	Effectiveness of Tai Chi on older adults: A systematic review of systematic reviews with re-meta-analysis	Population
Li et al. 2016	Effect of Exergames on Depression: A Systematic Review and Meta-Analysis	Population
Li et al. 2021	Effects of exercise on depressive symptoms among nursing home residents: A meta-analysis	Duplicate
Li et al. 2021	A systematic review on effects of green exercise on heart rate variability and blood pressure	Language
Li et al. 2021	Effects of exercise on depressive symptoms among nursing home residents: a meta-analysis	Population
Li et al. 2023	Effects of Music, Massage, Exercise, or Acupuncture in the Treatment of Depression Among College Students: A Network Meta-Analysis	Population
Li et al. 2023	Comparing the efficacy of different types of exercise for the treatment and prevention of depression in youths: a systematic review and network meta-analysis	Population
Lin et al. 2022	Effects of qigong exercise on the physical and mental health of college students: a systematic review and Meta-analysis	Population
Lin et al. 2022	Efficacy of Prenatal Yoga in the Treatment of Depression and Anxiety during Pregnancy: A Systematic Review and Meta-Analysis	Population
Liu et al. 2015	A systematic review and meta-analysis of the effects of Qigong and Tai Chi for depressive symptoms	Population
Liu et al. 2022	Physical exercise interventions for perinatal depression symptoms in women: A systematic review and meta-analysis	Population
Liu et al. 2023	Summary of the effect of an exercise intervention on antenatal depression and the optimal program: a systematic review and Meta-analysis	Population
Luberto et al. 2013	Integrative medicine for treating depression: an update on the latest evidence	Study design
Luo et al. 2022	Intervention of Physical Activity for University Students with Anxiety and Depression during the COVID-19 Pandemic Prevention and Control Period: A Systematic Review and Meta-Analysis	Population
Makone et al. 2023	Effect of Bhramari Pranayam on cardiovascular, autonomic and central nervous system - A systematic review	Population

Mala et al. 2012	Dance/Movement Therapy (D/MT) for depression: A scoping review	Study design
Maleki et al. 2020	Community-Based Preventive Interventions for Depression and Anxiety in Women	Population
Mammenga et al. 2021	Complementary and Alternative Treatments for Postpartum Depression	Study design
Manber et al. 2002	Alternative treatments for depression: empirical support and relevance to women	Study design
Manser et al. 2021	Can Reactivity of Heart Rate Variability Be a Potential Biomarker and Monitoring Tool to Promote Healthy Aging? A Systematic Review With Meta-Analyses	Comparator
Marasingha-Arachchige et al. 2020	Factors that affect heart rate variability following acute resistance exercise: A systematic review and meta-analysis	Intervention
Marconcin et al.2021	Effects of Exercise during Pregnancy on Postpartum Depression: A Systematic Review of Meta-Analyses	Population
Marinelli et al. 2024	Resistance training and combined resistance and aerobic training as a treatment of depression and anxiety symptoms in young people: A systematic review and meta-analysis,"Marinelli, R.	Population
Marques et al. 2020	The effect of muscular strength on depression symptoms in adults: A systematic review and meta-analysis	Population
Marquez et al. 2020	A systematic review of physical activity and quality of life and well-being	Population
Martins-Pinge et al. 2011	Cardiovascular and autonomic modulation by the central nervous system after aerobic exercise training	Study design
Martland et al. 2019	Can high intensity interval training (HIIT) improve physical and mental health outcomes? A metareview of the global benefits of hiit and focused systematic review of the effects of hiit in mental disorders	Duplicate
Martland et al. 2020	Can high-intensity interval training improve physical and mental health outcomes? A meta-review of 33 systematic reviews across the lifespan	Study design
Martland et al. 2023	The efficacy of exercise interventions for all types of inpatients across mental health settings: A systematic review and meta-analysis of 47 studies,"Martland, R.	Duplicate
McCall et al. 2013	Overview of systematic reviews: yoga as a therapeutic intervention for adults with acute and chronic health conditions	Study design
McCloskey et al. 2019	Complementary health approaches for postpartum depression: A systematic review	Population
McCurdy et al. 2017	Effects of Exercise on Mild-to-Moderate Depressive Symptoms in the Postpartum Period: A Meta-analysis	Population
Mead et al. 2008	Exercise for depression	Duplicate
Meda et al. 2023	Yoga: A novel modality for cardiovascular disease prevention	Study design
Meekums et al. 2005	Dance movement therapy for depression	Population
Mehta et al.	Yoga as a complementary therapy for clinical depression	Population
Meister et al. 2017	A Systematic Review of Mechanisms of Change in Body-Oriented Yoga in Major Depressive Disorders	Outcome
Meyer et al. 2018	Exercise for the prevention and treatment of depression	Study design

Milanović et al. 2019	Broad-spectrum physical fitness benefits of recreational football: a systematic review and meta-analysis	Population
Miller et al. 2019	Comparative effectiveness of three exercise types to treat clinical depression in older adults: A systematic review and network meta-analysis of randomised controlled trials	Population
Miller et al. 2021	Aerobic, resistance, and mind-body exercise are equivalent to mitigate symptoms of depression in older adults: A systematic review and network meta-analysis of randomised controlled trials	Population
Miller et al. 2021	Aerobic, resistance, and mind-body exercise are equivalent to mitigate symptoms of depression in older adults: A systematic review and network meta-analysis of randomised controlled trials	Population
Mojtabavi et al. 2020	Can music influence cardiac autonomic system? A systematic review and narrative synthesis to evaluate its impact on heart rate variability	Intervention
Moraes et al. 2007	Physical exercise in the treatment of depression in the elderly: A systematic review	Language
Moraes et al. 2007	Physical exercise in the treatment of depression in the elderly: A systematic review	Language
Moreira et al. 2020	Exercise and cardiac health: physiological and molecular insights	Study design
Morgan et al. 2015	Effects of physical exercise on central nervous system functions: a review of brain region specific adaptations	Population
Morlin et al. 2020	Bradycardia in athletes: does the type of sport make any difference? - a systematic review	Outcome
Morres et al. 2022	Exercise for perinatal depressive symptoms: A systematic review and meta-analysis of randomized controlled trials in perinatal health services	Population
Mosley et al. 2022	A scoping review of heart rate variability in sport and exercise psychology	Study design
Mura et al. 2013	Physical activity in depressed elderly. A systematic review	Population
Mygind et al. 2021	Effects of public green space on acute psychophysiological stress response: A systematic review and meta-analysis of the experimental and quasi-experimental evidence	Intervention
Nahas et al. 2011	Complementary and alternative medicine for the treatment of major depressive disorder	Study design
Nakamura et al. 2018	Physical activity during pregnancy and postpartum depression: Systematic review and meta-analysis	Population
Ng et al. 2018	A meta-analysis of the effectiveness of yoga-based interventions for maternal depression during pregnancy	Population
Noetel et al. 2024	Effect of exercise for depression: systematic review and network meta-analysis of randomised controlled trials	Population
Noetel et al. 2024	Effect of exercise for depression: systematic review and network meta-analysis of randomised controlled trials - correction	Study design
Norhamazi et al. 2022	Exercise intervention up to 6 months postpartum improves the outcome of Edinburgh postnatal depression scale	Population
North et al. 1988	The effect of exercise on depression: a meta-analysis	Study design
North et al. 1990	Effect of exercise on depression	Duplicate

North et al. 1990	Effect of exercise on depression	Study design
Nystrom et al. 2015	Treating Major Depression with Physical Activity: A Systematic Overview with Recommendations	Outcome
Oh et al. 2013	Effects of qigong on depression: a systemic review	Population
Park et al. 2014	Effects of exercise programs on depressive symptoms, quality of life, and self-esteem in older people: a systematic review of randomized controlled trials	Population
Pascoe et al. 2020	Exercise interventions for mental disorders in young people: a scoping review	Population
Patel et al. 2012	The effects of yoga on physical functioning and health related quality of life in older adults: a systematic review and meta-analysis	Population
Patiño Villada et al. 2013	Physical Exercise and Depression in the Elderly : A Systematic Review	Language
Pavey et al. 2011	Republished research: Effect of exercise referral schemes in primary care on physical activity and improving health outcomes: systematic review and meta-analysis	Population
Payne et al. 2013	Meditative movement for depression and anxiety	Study design
Paz Fernandez et al. 2021	Effects of exercise in pregnant and postpartum depression. A systematic review	Language
Pearsall et al. 2014	Exercise therapy in adults with serious mental illness: a systematic review and meta-analysis	Population
Pentland et al. 2022	Does Walking Reduce Postpartum Depressive Symptoms? A Systematic Review and Meta-Analysis of Randomized Controlled Trials	Population
Pérez Bedoya et al. 2023	Physical exercise and major depressive disorder in adults: systematic review and meta-analysis	Population
Perez-Lopez et al. 2017	Effects of programmed exercise on depressive symptoms in midlife and older women: A meta-analysis of randomized controlled trials	Population
Perez-Quilis et al. 2017	Modulation of Heart Rate by Acute or Chronic Aerobic Exercise. Potential Effects on Blood Pressure Control	Study design
Perraton et al. 2009	Exercise parameters in the treatment of clinical depression: a systematic review of randomized controlled trials	Outcome
Person et al. 2022	(PO-075) A Review of Alternative Health Interventions in the Treatment of Perinatal Depression	Study design
Pessoa et al. 2019	Dance therapy in aging: A systematic review	Population
Pettrey et al 2024	Physical Exercise as an Intervention for Depression: Evidence for Efficacy and Mu-Opioid Receptors as a Mechanism of Action	Study design
Pilkington et al. 2006	Complementary medicine for depression	Study design
Pinquart et al. 2007	Effects of psychotherapy and other behavioral interventions on clinically depressed older adults: A meta-analysis	Population
Piva et al. 2022	Effects of forest walking on physical and mental health in elderly populations: a systematic review	Study design

Piva et al. 2023	Exercise program for the management of anxiety and depression in adults and elderly subjects: Is it applicable to patients with post-covid-19 condition? A systematic review and meta-analysis	Population
Pizzoli et al. 2021	A meta-analysis on heart rate variability biofeedback and depressive symptoms	Intervention
Pizzoli et al. 2021	A meta-analysis on heart rate variability biofeedback and depressive symptoms	Intervention
Poquet et al. 2015	Exercise for the management of depression (PEDro synthesis)	Study design
Portugal et al. 2013	Neuroscience of exercise: From neurobiology mechanisms to mental health	Study design
Potes et al. 2017	Tai Chi interventions in mental illness: Results from a pilot study in adults and a systematic review in older adults	Population
Poyatos et al. 2017	Effects of exercise-based interventions on postpartum depression: A meta-analysis of randomized controlled trials	Outcome
Price et al. 2020	Acute cardiovascular responses to interval exercise: A systematic review and meta-analysis	Intervention
Prinsloo et al. 2014	A Brief Review and Clinical Application of Heart Rate Variability Biofeedback in Sports, Exercise, and Rehabilitation Medicine	Study design
Pritchett et al. 2018	Does aerobic exercise reduce postpartum depressive symptoms? A systematic review and meta-analysis	Population
Prudente et al. 2024	Effect of Dancing Interventions on Depression and Anxiety Symptoms in Older Adults: A Systematic Review and Meta-Analysis	Population
Pulcinelli et al. 2010	Antidepressant effect of exercise on individuals with mental disorders to estimate the performance kinematics parameters in speed races	Language
Qaseem et al. 2016	Nonpharmacologic Versus Pharmacologic Treatment of Adult Patients With Major Depressive Disorder: A Clinical Practice Guideline From the American College of Physicians	Study design
Qian et al. 2020	The Effectiveness of Virtual Reality Exercise on Individual's Physiological, Psychological and Rehabilitative Outcomes: A Systematic Review	Population
Quirk et al. 2017	Understanding the experience of initiating community-based physical activity and social support by people with serious mental illness: a systematic review using a meta-ethnographic approach	Population
Quirk et al. 2017	Understanding the experience of initiating community-based group physical activity by people with serious mental illness: A systematic review using a meta-ethnographic approach	Study design
Radmark et al. 2020	Mind and body exercises: Associations with mental health, antidepressant medication, autonomic functioning and inflammatory biomarkers	Population
Raffin et al. 2019	Exercise prevents age heart rate variability decline in the elderly: A systematic review and meta-analysis	Duplicate
Raffin et al. 2019	Exercise increases heart rate variability in older people: A systematic review and meta-analysis	Duplicate
Rahman et al. 2017	Current evidence and use of physical activity in the treatment of mental illness: A literature review	Study design

Ramos et al. 2015	The impact of high-intensity interval training versus moderate-intensity continuous training on vascular function: a systematic review and meta-analysis	Population
Ranjbar et al. 2015	Depression and Exercise: A Clinical Review and Management Guideline	Study design
Ravindran et al. 2009	Complementary and alternative treatments for depression	Study design
Rebar et al. 2015	A meta-meta-analysis of the effect of physical activity on depression and anxiety in non-clinical adult populations	Study design
Regan et al. 2015	Yoga for prenatal depression: a systematic review and meta-analysis	Duplicate
Rethorst et al. 2013	Evidence-based recommendations for the prescription of exercise for major depressive disorder	Study design
Richardson et al. 2005	Integrating physical activity into mental health services for persons with serious mental illness	Study design
Rittiwong et al. 2023	The Effects of Dance Interventions on Depression in Older Adults: A Meta-Analysis	Population
Rittiwong et al. 2023	The Effects of Dance Interventions on Depression in Older Adults: A Meta-Analysis	Population
Roete et al. 2021	A Systematic Review on Markers of Functional Overreaching in Endurance Athletes	Population
Rogers et al. 2009	A review of clinical trials of tai chi and qigong in older adults	Population
Rosenbaum et al. 2014	Physical activity interventions for people with mental illness: a systematic review and meta-analysis	Population
Rosenbaum et al. 2015	Online physical activity interventions for mental disorders: A systematic review	Population
Rosenbaum et al. 2015	Implementing evidence-based physical activity interventions for people with mental illness: an Australian perspective	Study design
Rosenwinkel et al. 2001	Exercise and autonomic function in health and cardiovascular disease	Study design
Ruiz et al. 2022	Winning The Game Against Depression: A Systematic Review of Video Games for the Treatment of Depressive Disorders	Population
Rupp et al. 2024	Walking the Black Dog: A systematic review and meta-analysis on the effect of walking interventions on depressive symptom severity	Population
Saeed et al. 2019	Depression and Anxiety Disorders: Benefits of Exercise, Yoga, and Meditation	Study design
Saligheh et al. 2017	Can exercise or physical activity help improve postnatal depression and weight loss? A systematic review	Duplicate
Saligheh et al. 2017	Does exercise or physical activity intervention improve postnatal depression and weight loss? A systematic review	Population
Salihu et al. 2021	The effect of dancing interventions on depression symptoms, anxiety, and stress in adults without musculoskeletal disorders: An integrative review and meta-analysis	Population
Samadi 2016	Exercise for Depression During Pregnancy	Study design
Sanchez-Delgado et al. 2023	Physical Exercise Effects on Cardiovascular Autonomic Modulation in Postmenopausal Women-A Systematic Review and Meta-Analysis	Population

Sanchez-Polan et al. 2021	Exercise During Pregnancy and Prenatal Depression: A Systematic Review and Meta-Analysis	Outcome
Sanchez-Polan et al. 2021	Exercise During Pregnancy and Prenatal Depression: A Systematic Review and Meta-Analysis	Population
Sani et al. 2023	Tai Chi Exercise for Mental and Physical Well-Being in Patients with Depressive Symptoms: A Systematic Review and Meta-Analysis	Duplicate
Sant'Ana et al. 2020	Effects of Cardiovascular Interval Training in Healthy Elderly Subjects: A Systematic Review	Duplicate
Santos et al. 2020	Effectiveness of senior dance in the health of adults and elderly people: An integrative literature review,	Population
Sardeli et al. 2017	Resistance training and cardiovascular autonomic modulation in humans: a systematic review and meta-analysis	Population
Schiweck et al. 2018	Heart rate and high frequency heart rate variability during stress as biomarker for clinical depression. A systematic review	Intervention
Schuch et al. 2011	The impact of exercise on quality of life within exercise and depression trials: A systematic review	Outcome
Schuch et al. 2011	"The impact of exercise on quality of life within exercise and depression trials: A systematic review": Corrigendum	Study design
Schuch et al. 2016	Moderators of response in exercise treatment for depression: A systematic review	Intervention
Schuch et al. 2016	Physical activity and sedentary behavior in people with major depressive disorder: A systematic review and meta-analysis	Outcome
Schuch et al. 2016	Exercise improves physical and psychological quality of life in people with depression: A meta-analysis including the evaluation of control group response	Outcome
Schuch et al. 2017	Physical activity and sedentary behavior in people with major depressive disorder: A systematic review and meta-analysis: Corrigendum	Study design
Schuch et al. 2018	Correction: Physical activity and incident depression: A meta-analysis of prospective cohort studies	Study design
Scully et al. 1998	Physical exercise and psychological well being: a critical review	Study design
Seo et al. 2018	Effects of Exercise Interventions on Depressive Symptoms Among Community-Dwelling Older Adults in the United States: A Systematic Review	Population
Seshadri et al. 2021	Exercise, Yoga, and Tai Chi for Treatment of Major Depressive Disorder in Outpatient Settings: A Systematic Review and Meta-Analysis	Duplicate
Setayesh et al. 2023	The impact of resistance training on brain-derived neurotrophic factor and depression among older adults aged 60 years or older: A systematic review and meta-analysis of randomized controlled trials	Population
Shaffer et al. 2014	A healthy heart is not a metronome: An integrative review of the heart's anatomy and heart rate variability	Study design
Shao et al. 2015	Autonomic nervous system: Its response and adaptation to exercises	Language
Sharma et al. 2013	Tai Chi as an alternative or complementary therapy for patients with depression: A systematic review	Population

Shengyu et al. 2020	Exercise Intervention Options for Depression During the Coronavirus Disease (COVID-19) Epidemic	Population
Shi et al. 2016	Effect of exercise on depression in university students: a meta-analysis of randomized controlled trials	Population
Shivakumar et al. 2011	Antenatal depression: a rationale for studying exercise	Study design
Silva et al. 2012	Mind-body medicine therapies for a range of depression severity: a systematic review	Duplicate
Silva et al. 2012	Mind-body medicine therapies for the depression spectrum: A systematic review	Study design
Silvia et al. 2014	Does Baseline Heart Rate Variability Reflect Stable Positive Emotionality?	Study design
Singh et al. 2003	Circadian heart rate and blood pressure variability considered for research and patient care	Study design
Singh et al. 2022	An overview of benefit of Yoga on Selected Physiological parameters	Study design
Sivaramakrishnan et al. 2019	The effects of yoga compared to active and inactive controls on physical function and health related quality of life in older adults- systematic review and meta-analysis of randomised controlled trials	Population
Smith et al. 2019	The effect of complementary medicines and therapies on maternal anxiety and depression in pregnancy: A systematic review and meta-analysis	Population
Smith, et al. 2021	The Role of Exercise in Management of Mental Health Disorders: An Integrative Review	Study design
Solloway et al. 2016	An evidence map of the effects of Tai CHI	Population
Solloway et al. 2016	An evidence map of the effect of Tai Chi on health outcomes	Population
Song et al. 2021	Effects of aerobic exercise, traditional Chinese exercises, and meditation on depressive symptoms of college student: A meta-analysis of randomized controlled trials	Population
Song et al. 2021	Effects of aerobic exercise, traditional Chinese exercises, and meditation on depressive symptoms of college student: A meta-analysis of randomized controlled trials	Population
Soucy Chartier et al. 2013	Behavioral activation and depression: a contextual treatment approach	Language
Sowden et al. 2009	The impact of mental illness on cardiac outcomes: A review for the cardiologist	Study design
Stammes et al. 2009	Physical training to treat depression	Duplicate
Stammes et al. 2009	Fysieke training bij depressie	Language
Stanton et al. 2013	Is cardiovascular or resistance exercise better to treat patients with depression? A narrative review	Study design
Stanton et al. 2014	Exercise and the treatment of depression: a review of the exercise program variables	Outcome
Steenhuis et al. 2015	Treating Depressive Symptoms in Psychosis: A Network Meta-Analysis on the Effects of Non-Verbal Therapies	Population
Steere et al. 2020	Autonomic Nervous System Changes in Individuals with Anxiety and Depression Across the Lifespan: A Systematic Review	Study design
Steffens et al. 2013	Exercise for late-life depression? It depends	Study design
Steinman et al. 2007	Recommendations for treating depression in community-based older adults	Study design

Stich et al. 1998	A meta-analysis of physical exercise as a treatment for symptoms of anxiety and depression	Study design
Stubbs et al. 2016	Exercise improves cardiorespiratory fitness in people with depression: A meta-analysis of randomized control trials	Outcome
Stubbs et al. 2016	Dropout from exercise randomized controlled trials among people with depression: A meta-analysis and meta regression	Outcome
Stubbs et al. 2016	Challenges Establishing the Efficacy of Exercise as an Antidepressant Treatment: A Systematic Review and Meta-Analysis of Control Group Responses in Exercise Randomised Controlled Trials	Population
Stubbs et al. 2018	EPA guidance on physical activity as a treatment for severe mental illness: a meta-review of the evidence and Position Statement from the European Psychiatric Association (EPA), supported by the International Organization of Physical Therapists in Mental Health (IOPTMH)	Study design
Stubbs et al. 2019	Exercise-based interventions for mental illness: Physical activity as part of clinical treatment	Study design
Sud et al. 2021	Mind-body interventions for depressive symptoms in chronic pain: A systematic review of meta-analyses	Population
Suetani et al. 2019	Physical activity of people with mental disorders compared to the general population: a systematic review of longitudinal cohort studies	Population
Swinson et al. 2020	Green walking groups: A mixed-methods review of the mental health outcomes for adults with mental health problems	Population
Takacs et al. 2014	Regular physical activity and mental health. The role of exercise in the prevention of, and intervention in depressive disorders	Language
Tao et al. 2024	Effects of high-intensity interval training on depressive symptoms: A systematic review and meta-analysis	Population
Tavares et al. 2023	Effects of exercise on cognitive functioning in adults with serious mental illness: A meta analytic review	Outcome
Teles Morlin et al. 2020	Bradycardia in athletes: does the type of sport make any difference? - A systematic Review	Outcome
Teychenne et al. 2008	Physical activity and likelihood of depression in adults: A review	Population
Teychenne et al. 2013	Physical activity, sedentary behavior, and postnatal depressive symptoms: a review	Population
Thachil et al. 2007	The evidence base of complementary and alternative therapies in depression	Population
Thomas et al. 2021	Cardiovascular Responses to Muscle Stretching: A Systematic Review and Meta-analysis	Outcome
Tiwari et al. 2021	Analysis of Heart Rate Variability and Implication of Different Factors on Heart Rate Variability	Outcome
Togo et al. 2009	Heart rate variability in occupational health - a systematic review	Intervention
Tomes et al. 2020	Relationships Between Heart Rate Variability, Occupational Performance, and Fitness for Tactical Personnel: A Systematic Review	Intervention
Tonello et al. 2014	The role of physical activity and heart rate variability for the control of work related stress	Population

Tong et al. 2024	The effectiveness of self-guided interventions in adults with depressive symptoms: a systematic review and meta-analysis	Intervention
Torres et al. 2012	Physical Activity Effects on Depressive Symptoms in Black Adults	Population
Tse et al. 2015	Effect of Low-intensity Exercise on Physical and Cognitive Health in Older Adults: a Systematic Review	Population
Uebelacker et al. 2010	Hatha yoga for depression: critical review of the evidence for efficacy, plausible mechanisms of action, and directions for future research	Population
Valente et al. 2017	The impact of euthmy therapy in heart rate variability: A systematic review	Outcome
Van Der Woude et al. 2015	Health status and quality of life in postpartum women: A systematic review of associated factors	Population
van Zyl et al. 2008	Effects of antidepressant treatment on heart rate variability in major depression: a quantitative review	Population
Vancampfort et al. 2017	Sedentary behavior and physical activity levels in people with schizophrenia, bipolar disorder and major depressive disorder: a global systematic review and meta-analysis	Outcome
Vancampfort et al. 2017	Cardiorespiratory Fitness in Severe Mental Illness: A Systematic Review and Meta-analysis	Outcome
Vancampfort et al. 2020	The efficacy of physical activity on psychiatric symptoms and physical health in people with psychiatric disorders: a systematic review of recent meta-analyses	Language
Vancampfort et al. 2021	The efficacy of meditation-based mind-body interventions for mental disorders: A meta-review of 17 meta-analyses of randomized controlled trials	Study design
Vasudev et al. 2015	Autonomic dysfunction and depression: A biomarker of MDD across the life span	Intervention
Vilella et al. 2014	Are there psychosocial benefits for Pilates practice? An analysis of the scientific literature	Language
Volaklis et al. 2019	Association between muscular strength and depressive symptoms : A narrative review	Intervention
Vollbehr et al. 2018	Hatha yoga for acute, chronic and/or treatment-resistant mood and anxiety disorders: A systematic review and meta-analysis: Correction	Study design
Vreijling et al. 2021	Reduced Heart Rate Variability in Patients with Medically Unexplained Physical Symptoms: A Meta-Analysis of HF-HRV and RMSSD	Population
Walter et al. 2017	A systematic review of mind and body complementary health practices for informal caregivers	Population
Wang 2005	Association between exercise therapy and depression	Study design
Wang et al. 2009	The effect of Tai Chi on psychosocial well-being: a systematic review of randomized controlled trials	Population
Wang et al. 2010	Tai Chi on psychological well-being: systematic review and meta-analysis	Population
Wang et al. 2013	The effect of qigong on depressive and anxiety symptoms: a systematic review and meta-analysis of randomized controlled trials	Population
Wang et al. 2014	Systematic review of yoga for depression and quality of sleep in the elderly	Language

Wang et al. 2014	The Effects of Tai Chi on Depression, Anxiety, and Psychological Well-Being: A Systematic Review and Meta-Analysis	Population
Wang et al. 2015	Effect of traditional Chinese exercise on the quality of life and depression for chronic diseases: a meta-analysis of randomised trials	Population
Wang et al. 2022	Yoga's Therapeutic Effect on Perinatal Depression: A Systematic Review and Meta-Analysis	Population
Wang et al. 2022	The Effects of Exercise Interventions on Mental Health in Chinese Older Adults	Population
Wang et al. 2024	Heart rate variability in generalized anxiety disorder, major depressive disorder and panic disorder: A network meta-analysis and systematic review	Duplicate
Wang, year unkown	Meta Analysis on Effects of Physical Exercise on the Depressive Symptoms of College Students in China	Population
Wanjau et al. 2023	Physical Activity and Depression and Anxiety Disorders: A Systematic Review of Reviews and Assessment of Causality	Study design
Weber et al. 2020	Effects of Mind-Body Interventions Involving Meditative Movements on Quality of Life, Depressive Symptoms, Fear of Falling and Sleep Quality in Older Adults: A Systematic Review with Meta-Analysis	Population
Wegner et al. 2014	Effects of exercise on anxiety and depression disorders: review of meta- analyses and neurobiological mechanisms	Study design
Wen, year unkown	Depression and exercise therapy	Language
Wieland et al. 2021	Evidence on yoga for health: A bibliometric analysis of systematic reviews	Population
Windle et al. 2010	Is exercise effective in promoting mental well-being in older age? A systematic review	Population
Wolf et al. 2020	Can physical activity protect against depression and anxiety during the COVID-19 pandemic? A rapid systematic review	Population
Wolf et al. 2020	Sports Activity and Mental Diseases	Study design
Wolf et al. 2021	Is Physical Activity Associated with Less Depression and Anxiety During the COVID-19 Pandemic? A Rapid Systematic Review	Duplicate
Wong et al. 2014	Lifestyle medicine for depression: A meta-analysis of randomized controlled trials	Population
Wong et al. 2014	Lifestyle medicine for depression: A meta-analysis of randomized controlled trials: Corrigendum	Study design
Wong et al. 2021	Effects of acute stretching exercise and training on heart rate variability: a review	Intervention
Wood 2014	Cardiac autonomic imbalance by social stress in rodents: understanding putative biomarkers	Study design
Wu et al. 2023	Effectiveness of yoga for major depressive disorder: A systematic review and meta-analysis	Duplicate
Xia et al. 2021	A Meta-analysis of Effect of Exercise on Postpartum Depression Symptoms	Duplicate
Xie et al. 2021	The Effects and Mechanisms of Exercise on the Treatment of Depression	Study design
Yan et al. 2021	Multidimensional analyses of the effect of exercise on women with depression: A meta-analysis	Population

Yan et al. 2021	Multidimensional analyses of the effect of exercise on women with depression	Population
Yen et al.2021	Virtual Reality Exergames for Improving Older Adults' Cognition and Depression: A Systematic Review and Meta-analysis of Randomized Control Trials	Population
Yin et al. 2014	The effect of Tai Chi and Qigong practice on depression and anxiety symptoms: A systematic review and meta-regression analysis of randomized controlled trials	Population
Yin et al. 2023	The comparative effects of Tai chi versus non-mindful exercise on measures of anxiety, depression and general mental health: A systematic review and meta-analysis	Population
Yoo et al. 2020	The psychological effects of water-based exercise in older adults: An integrative review	Population
Young et al. 2013	Effectiveness of physical activity interventions with healthy older women: A systematic review protocol	Study design
Young et al. 2018	Efficacy of online lifestyle interventions targeting lifestyle behaviour change in depressed populations: A systematic review	Intervention
Zeng et al. 2018	Virtual reality exercise for anxiety and depression: A preliminary review of current research in an emerging field	Population
Zhang et al. 2021	Physical activity and depression in older adults: the knowns and unknowns	Comparator
Zhang et al. 2021	Physical activity and depression in older adults: The knowns and unknowns	Study design
Zhang et al. 2023	Research progress of exercise therapy for depressive disorder	Language
Zhao et al. 2020	Exercise, brain plasticity, and depression	Study design
Zhou et al. 2024	Tai Chi Effects on Heart Rate Variability: A Systematic Review and Meta-Analysis	Population
Zhu et al. 2021	The effect of music, massage, yoga and exercise on antenatal depression: A meta-analysis	Population
Zhu et al. 2021	The effect of music, massage, yoga and exercise on antenatal depression: A meta-analysis	Population
Zichao et al. 2022	Effect of exercise interventions on the depression of older adults. A meta-analysis	Population
Zope et al. 2013	Sudarshan kriya yoga: Breathing for health	Study design
Zou et al. 2013	Effects of Mind-Body Exercises (Tai Chi/Yoga) on Heart Rate Variability Parameters and Perceived Stress: A Systematic Review with Meta-Analysis of Randomized Controlled Trials	Population
Zou et al. 2018	Mindfulness-Based Baduanjin Exercise for Depression and Anxiety in People with Physical or Mental Illnesses: A Systematic Review and Meta-Analysis	Duplicate
Zou et al. 2018	Mindfulness-Based Baduanjin Exercise for Depression and Anxiety in People with Physical or Mental Illnesses: A Systematic Review and Meta-Analysis	Population

The table is sorted alphabetically by reference.

S4 – Supplement 4 AMSTAR 2 quality of appraisal of reviews

Review	AMSTAR 2 Items																Overall quality rating
	1	2	3	4	5	6	7	8	9	10	11	12	13	14	15	16	
Physical activity and major depressive disorder																	
Asher et al. 2017	Y	Y	Y	PY	Y	Y	PY	Y	Y	N	Y	N	N	N	Y	Y	Low
Blake et al. 2009	Y	N	Y	PY	N	Y	PY	Y	N	N	N/A	N/A	Y	Y	N/A	Y	Critically Low
Bridle et al. 2012	Y	N	Y	PY	Y	Y	PY	Y	Y	N	Y	Y	Y	Y	Y	N	Low
Carneiro et al. 2020	Y	Y	Y	PY	Y	Y	PY	Y	Y	N	Y	Y	Y	Y	N	Y	Low
Chen et al. 2018	Y	N	Y	PY	Y	Y	N	Y	N	N	N/A	N/A	N	N	N/A	N	Critically Low
Chen et al. 2019	Y	Y	Y	Y	Y	Y	PY	Y	Y	N	Y	Y	Y	Y	N	Y	Low
Chi et al. 2013	Y	N	Y	PY	Y	Y	PY	PY	Y	N	Y	Y	Y	Y	Y	N	Low
Cooney et al. 2013	Y	Y	Y	Y	Y	Y	Y	Y	Y	N	Y	Y	Y	Y	Y	Y	High
Correia et al. 2024	Y	Y	Y	PY	Y	Y	PY	PY	Y	N	Y	Y	N	N	N	Y	Critically Low
Craft et al. 1998	Y	N	N	PY	N	N	N	N	N	N	Y	N	N	N	N	N	Critically Low
Cramer et al. 2013	Y	PY	Y	Y	Y	Y	PY	Y	Y	N	Y	Y	Y	Y	Y	Y	Moderate
Cramer et al. 2017	Y	N	Y	PY	Y	Y	PY	Y	Y	N	N/A	N/A	Y	Y	N/A	Y	Low
da Cunha et al. 2023	Y	Y	Y	Y	Y	Y	PY	Y	Y	N	Y	Y	Y	Y	Y	Y	Moderate
Danielsson et al. 2013	Y	N	Y	PY	N	Y	PY	Y	Y	N	Y	Y	Y	Y	Y	Y	Low
de Souza Moura et al. 2015	Y	N	Y	PY	Y	Y	PY	PY	PY	N	N/A	N/A	N	N	N/A	N	Critically Low
Eriksson et al. 2011	Y	N	Y	PY	N	N	N	Y	N	N	N/A	N/A	N	N	N/A	N	Critically Low
Feng et al. 2024	Y	Y	Y	PY	Y	Y	PY	PY	Y	N	Y	Y	N	Y	Y	Y	Low
Frazer et al. 2011	Y	N	Y	PY	N	N	N	N	N	N	N/A	N/A	N	N	N/A	Y	Critically Low
Gartlehner et al. 2023	Y	Y	Y	Y	Y	Y	Y	Y	Y	Y	Y	Y	Y	Y	Y	Y	High
Guo et al. 2019	Y	N	Y	PY	N	Y	PY	Y	PY	N	Y	N	N	Y	Y	Y	Critically Low
Guo et al. 2020	Y	N	Y	PY	N	Y	N	PY	Y	N	Y	Y	Y	N	Y	Y	Critically Low
Heinzel et al. 2015	Y	N	Y	PY	Y	Y	PY	Y	Y	N	Y	Y	Y	Y	N	Y	Critically Low
Heissel et al. 2023	Y	Y	Y	Y	Y	Y	Y	Y	Y	N	Y	Y	Y	Y	Y	Y	High
Holvast et al. 2017	Y	Y	Y	Y	Y	Y	PY	Y	Y	N	Y	Y	Y	Y	N	Y	Low

Hooper et al. 2021	Y	Y	Y	PY	Y	Y	PY	PY	Y	N	Y	Y	Y	Y	N	Y	Critically Low
Josefsson et al. 2012	Y	N	Y	Y	N	N	PY	Y	PY	N	Y	Y	Y	N	N	N	Critically Low
Karkou et al. 2019	Y	Y	Y	PY	Y	Y	PY	Y	Y	N	Y	Y	Y	Y	N	N	Low
Krogh et al. 2017	Y	Y	Y	Y	Y	Y	PY	Y	Y	N	Y	Y	Y	Y	Y	Y	Moderate
Kvam et al. 2016	Y	N	Y	Y	N	Y	Y	Y	Y	N	Y	Y	Y	Y	Y	Y	Low
Lawlor et al. 2001	Y	N	Y	Y	Y	Y	Y	Y	Y	N	Y	Y	Y	N	N	Y	Critically Low
Lee et al. 2021	Y	Y	Y	PY	Y	Y	PY	Y	Y	N	Y	Y	Y	Y	Y	Y	Moderate
Martinez-Calderon et al. 2024	Y	Y	Y	Y	N	N	Y	Y	Y	N	Y	Y	Y	Y	Y	Y	Moderate
Martland et al. 2020	Y	Y	Y	PY	Y	Y	Y	Y	Y	N	Y	Y	Y	N	N	Y	Low
Martland et al. 2023	Y	Y	Y	PY	Y	Y	PY	Y	Y	N	Y	Y	Y	Y	Y	Y	Moderate
Md Zemberi et al. 2020	Y	N	Y	PY	Y	N	N	Y	PY	N	N/A	N/A	N	Y	N/A	Y	Critically Low
Mead 2008	Y	Y	Y	Y	Y	Y	Y	Y	Y	N	Y	Y	Y	Y	Y	Y	High
Miao et al. 2023	Y	Y	Y	Y	Y	Y	PY	Y	Y	N	Y	Y	Y	Y	Y	Y	Moderate
Millman et al. 2021	Y	N	Y	PY	N	N	PY	Y	N	N	N/A	N/A	N	Y	N/A	Y	Critically Low
Morres et al. 2018	Y	N	Y	Y	Y	Y	PY	Y	Y	N	Y	Y	Y	Y	Y	N	Low
Mura, Moro et al. 2014	Y	N	Y	PY	N	N	PY	PY	Y	N	N/A	N/A	Y	N	N/A	Y	Low
Mura, Sancassiani et al. 2014	Y	N	Y	PY	N	N	PY	PY	PY	N	N/A	N/A	Y	N	N/A	Y	Low
Nanthakumar 2020	N	N	N	PY	N	N	N	Y	N	N	N/A	N/A	N	N	N/A	N	Critically Low
Nebiker et al. 2018	Y	N	Y	PY	Y	Y	PY	Y	Y	N	Y	Y	Y	Y	Y	Y	Low
Pilkington et al. 2005	Y	N	N	PY	N	Y	Y	Y	Y	N	N/A	N/A	Y	Y	N/A	Y	Low
Recchia et al. 2022	Y	Y	Y	Y	Y	Y	PY	PY	Y	N	Y	Y	Y	Y	Y	Y	Moderate
Rethorst et al. 2009	N	N	Y	PY	N	Y	N	Y	N	N	Y	N	N	N	N	Y	Critically Low
Rhyner et al. 2015	Y	N	Y	PY	N	N	N	Y	N	N	Y	Y	Y	Y	Y	N	Critically Low
Robertson et al. 2012	Y	N	Y	Y	N	Y	PY	Y	PY	N	Y	Y	Y	Y	N	N	Critically Low
Rossi et al. 2024	Y	Y	Y	Y	Y	Y	PY	Y	Y	N	Y	Y	Y	N	Y	Y	Moderate
Sani et al. 2023	Y	Y	Y	PY	Y	Y	PY	PY	Y	N	Y	Y	Y	Y	Y	Y	Moderate
Schuch, Vancampfort, Richards et al. 2016	Y	N	Y	PY	Y	Y	PY	PY	Y	N	Y	Y	Y	Y	Y	Y	Low
Schuch, Vancampfort, Rosenbaum, et al. 2016	Y	N	Y	PY	Y	Y	PY	PY	Y	N	Y	Y	Y	Y	Y	Y	Low

Seshadri et al. 2021	Y	N	Y	PY	Y	Y	PY	Y	Y	N	Y	Y	Y	Y	Y	Y	Low
Silveira et al. 2013	N	N	Y	PY	Y	N	PY	PY	N	N	Y	N	N	Y	N	Y	Critically Low
Sjosten et al. 2006	Y	N	Y	PY	N	N	PY	Y	PY	N	N/A	N/A	Y	Y	N/A	N	Critically Low
Stanton et al. 2014	Y	N	Y	PY	N	N	PY	Y	PY	N	N/A	N/A	Y	Y	N/A	N	Critically Low
Stathopoulou et al. 2006	N	N	Y	PY	N	N	N	PY	N	N	Y	N	N	N	Y	N	Critically Low
Sukhato et al. 2016	Y	N	Y	PY	Y	Y	PY	PY	Y	N	Y	Y	Y	Y	Y	Y	Low
Tang et al. 2024	Y	Y	Y	Y	Y	Y	PY	Y	Y	N	Y	Y	Y	Y	Y	Y	Moderate
Tian et al. 2024	Y	Y	Y	Y	Y	Y	PY	Y	Y	N	Y	Y	N	Y	Y	Y	Low
Tsang et al. 2008	N	N	Y	PY	Y	N	Y	Y	Y	N	N/A	N/A	Y	N	N/A	N	Low
Vollbehr et al. 2018	Y	N	Y	PY	Y	Y	PY	Y	Y	N	Y	Y	Y	Y	Y	Y	Low
Wu et al. 2023	Y	Y	Y	PY	Y	Y	PY	Y	Y	N	Y	Y	Y	N	Y	Y	Moderate
Yang et al. 2023	Y	N	N	PY	N	Y	PY	PY	Y	N	Y	Y	N	Y	Y	Y	Critically Low
Yu et al. 2022	Y	PY	Y	Y	Y	Y	PY	Y	Y	N	Y	N	Y	Y	Y	Y	Moderate
Zeng et al. 2023	Y	PY	Y	PY	Y	Y	PY	Y	Y	N	Y	Y	Y	Y	Y	Y	Moderate
Zielinski et al. 2021	Y	N	N	PY	Y	Y	N	Y	Y	Y	N/A	N/A	Y	N	N/A	N	Critically Low
Zou et al. 2018	Y	N	Y	PY	Y	Y	PY	Y	Y	N	Y	Y	Y	Y	Y	Y	Low
Heart rate variability and physical activity																	
Bhati et al. 2018	Y	N	Y	PY	Y	Y	PY	Y	N	N	Y	Y	N	Y	N	Y	Critically Low
Casanova-Lizon et al. 2022	Y	Y	Y	Y	Y	Y	PY	Y	PY	N	Y	Y	N	Y	Y	Y	Low
Corso et al. 2022	N	N	Y	PY	N	N	PY	N	Y	N	N/A	N/A	Y	N	N/A	Y	Critically Low
Grassler et al. 2021a (young and middle age adults)	Y	N	Y	PY	Y	Y	N	Y	PY	N	N/A	N/A	N	Y	N/A	Y	Critically Low
Grassler et al. 2021b (middle age adults)	Y	Y	Y	PY	Y	Y	N	Y	Y	Y	N/A	N/A	N	Y	N/A	Y	Critically Low
Grässler et al. 2021c (older adults)	Y	Y	Y	PY	Y	Y	PY	Y	Y	N	N/A	N/A	Y	Y	N/A	Y	Moderate
Kingsley et al. 2016	Y	N	Y	PY	N	N	N	PY	N	N	N/A	N/A	N	N	N/A	Y	Critically Low
Maity et al. 2024	N	Y	Y	PY	Y	Y	PY	PY	N	N	Y	N	N	N	N	Y	Critically Low
Posadzki et al. 2015	Y	N	Y	PY	N	Y	PY	Y	Y	N	Y	Y	Y	Y	N	Y	Critically Low
Raffin et al. 2019	Y	N	Y	PY	Y	Y	PY	N	Y	N	Y	Y	Y	Y	Y	Y	Low
Sandercock et al. 2005	Y	N	N	PY	N	N	N	N	N	N	Y	N	N	Y	N	N	Critically Low

Sant'Ana et al. 2020	Y	N	Y	PY	Y	Y	N	Y	Y	N	N/A	N/A	N	Y	N/A	Y	Critically Low
Tyagi et al. 2016	Y	N	Y	Y	N	N	PY	PY	N	N	N/A	N/A	N	N	N/A	Y	Critically Low
Yang et al. 2024	Y	Y	Y	Y	Y	Y	PY	Y	Y	N	Y	Y	N	N	Y	Y	Low
Major depressive disorder and heart rate variability																	
Brown et al. 2018	Y	Y	N	PY	N	Y	PY	Y	Y	N	Y	Y	Y	Y	Y	Y	Moderate
Cukic et al. 2023	Y	N	Y	PY	N	N	PY	N	N	N	Y	N	N	N	N	Y	Critically Low
Kemp et al. 2010	Y	N	N	N	N	N	PY	PY	Y	N	Y	N	Y	Y	Y	Y	Critically Low
Koch et al. 2019	Y	N	Y	Y	Y	N	PY	Y	Y	N	Y	Y	N	Y	N	Y	Critically Low
Ramesh et al. 2023	Y	PY	N	PY	N	Y	PY	Y	Y	Y	N/A	N/A	Y	Y	N/A	N	Moderate
Rottenberg 2006	Y	N	N	PY	Y	N	N	PY	N	N	N	N	Y	N	N	Y	Critically Low
Wang et al. 2023	Y	Y	Y	Y	N	Y	PY	Y	Y	N	Y	Y	Y	Y	Y	Y	Moderate
Wu et al. 2023	Y	PY	Y	Y	Y	Y	PY	Y	Y	N	Y	Y	Y	Y	Y	Y	Moderate

AMSTAR 2 Items: 1) Inclusion criteria and the research question's Participant, Intervention, Comparator, and Outcome (PICO) components were aligned. 2) The review methods were established before the study's execution, and any significant deviations were adequately justified. 3) Explanation provided for the selection of study designs included in the review. 4) Employed a comprehensive search strategy. 5) Study selection was conducted in duplicate. 6) Data extraction was performed in duplicate. 7) A list of excluded studies was presented along with justifications. 8) Described the included studies in sufficient detail. 9) Utilized an appropriate technique to assess the risk of bias in the included studies. 10) Reported the sources of funding for the included studies. 11) Employed suitable methods for statistically combining results if a meta-analysis was conducted. 12) Evaluated the potential impact of individual study biases if a meta-analysis was conducted. 13) Took into account the risk of bias in individual studies when discussing the review results. 14) Explained and discussed any observed heterogeneity in the review results. 15) Investigated and discussed publication bias if a meta-analysis was performed. 16) Authors disclosed any potential conflicts of interest.

Abbreviations: N/A: No answer, N: No, PY: Partial yes, Y: Yes

49 **S5 - Supplement 5** Physical activity and major depressive disorder

Reference	Characteristics	Objective	Outcomes	Conclusions	ES	AMSTAR 2 Review Quality
Asher et al. 2017	Meta-Analysis, 3 studies included, AE, 357 individuals	Report the benefits and harms of exercise treatments with second-generation antidepressants (SGA) for MDD.	Treatment response rate	Comparing exercise with SGAs, no significant difference was found in treatment response rate. Exercise had lower response rates compared to St. John's worth. No significant differences were found between exercise and other interventions.	Treatment respond rate: Relative Risk MDD to SGA: 0.53 (0.22: 1.26), relative Risk MDD to St. John's worth: 0.42 (0.17: 1.02)	Low
Blake et al. 2009	No Meta-Analysis, 11 studies included, AE, RT, MBE, MT, 641 individuals with a age range of 65-82.4 years, Ratio male/female 1:4.4 to 1:1 in included studies	Assess the efficacy of physical exercise for the treatment of depressive symptoms in older adults.	Depression or mood scores on standardized scales	Post intervention: Out of 11 reviewed studies, seven provided findings on the immediate effect of exercise intervention.<br>Five of these studies showed significant positive results, indicating that exercise intervention improved depression or depressive symptoms.<br>In the medium term (3-12 months), there is limited or conflicting evidence of exercise benefits based on six studies.<br>In the long term (>12 months), two studies had mixed results.	N/A	Critically Low
Bridle et al. 2012	Meta-Analysis, 9 studies included, AE, RT, MBE, MT, 667 individuals (69% female) with mean age of 65-80 years	Estimate the effect of exercise on depressive symptoms among older people.	Depression determined by a clinically valid method of assessment, such as a clinical interview, clinician diagnosis or symptom checklist threshold	Findings suggest that prescribing structured exercise tailored to individual ability reduces depression severity in older people with clinically meaningful symptoms of depression.	Depression severity: -0.34 (-0.52: -0.17)	Low
Carneiro et al. 2020	Meta-Analysis, 4 studies included, RT, 259 individuals with a	Summarize the effects of supervised RT programs in people	Decrease of depression symptoms and depression severity	In two of the four combinations with different screening methods, the meta-analysis revealed significant benefits of RT in improving depressive	Reduction in depressive symptoms with different assessing methods: RT vs. Control (HAM-D, BDI, CES-D, GDS): 0.62	Low

	age range of 18-82 years	diagnosed with depression or depressive symptoms.	assessed by validated screening measure	symptoms. RT is a possible method to reduce symptoms of depression.	(0.03:1.20), RT vs. Control (HAMD-D, BDI, CES-D, HAM-D): 0.53 (0.02:1.03), RT vs. Control (HAM-D, CES-D): 0.39 (-0.05:0.84), RT vs. Control (HAM-D, CES-D, GDS): 0.48 (-0.06:1.02)	
Chen et al. 2018	No Meta-Analysis, 7 studies included, AE, RT, NT, MBE, MT, 473 individuals with a mean age of 74.3 ± 37.4 years	Elaborate the effects of exercise interventions on the two most common mental disorders depression and dementia facing older people.	Reduction of depression severity in older adults	The 7 trials consistently showed positive effects of exercise on depression severity in older adults.<br>These findings align with previous reviews, supporting the effectiveness of exercise as a no pharmacological treatment for reducing late-life depression severity.	N/A	Critically Low
Chen et al. 2019	Meta-Analysis, 10 studies included, AE, RT, MT, 3797 individuals with age range of 63.3-86.7 years	Assess the effectiveness of non-pharmacological interventions like exercise for seniors with depressive symptoms.	Efficacy of treatments to reduce depression symptoms	Physical activity and psychotherapy demonstrated statistically significant superiority over conventional treatment like pharmacological interventions.	Improvements depressive symptoms: physical activity vs. TAU: 2.25 (0.99: 3.56) , AE vs. TAU: 1.84 (0.39: 3.36), RT vs. TAU: 1.72 (0.06: 3.42)	Low
Chi et al. 2013	Meta-Analysis, 4 studies included, MBE, 404 individuals (65 % Women) with age range of 52-85 years	Evaluate the effectiveness of tai chi on reducing depressive symptoms among older adults.	Reduction of depressive symptoms in terms of rating scale scores	Tai Chi showed a significant impact on reducing depressive symptoms compared to waiting list control groups.<br>Interpretation of the findings should be cautious due to the limited number of studies included in the meta-analysis.	Reduction of depressive symptoms: Tai Chi vs. control-0.27 (-0.52: -0.02)	Low
Cooney et al. 2013	Meta-Analysis, 39 studies included, AE, RT, MT, 1356 individuals with a mean age range of 22-87.9 years	Determine the effectiveness of exercise in the treatment of depression in adults compared with no treatment or a comparator intervention.	Depression severity measured with Beck Depression Inventory (BDI) scores, Hamilton Rating Scale for Depression (HAMD) scores and other scales	Exercise may have a moderate-sized effect on depression, but the risks of bias in many trials suggest the effect may be small.<br>Uncertainty remains regarding the specific type, intensity, duration, and frequency of effective exercise.<br>Evidence suggests exercise may be as effective as psychological or pharmacological treatments, but the	Reduction depression severity: Exercise vs. No treatment or control: -0.62 (-0.81: -0.42), Exercise vs. No treatment or control (high quality studies): -0.18 (-0.47: 0.11), Exercise vs. No treatment or control (long-term follow-up data): -0.33 (-0.63: -0.03), Exercise vs. psychological therapy: -0.03 (-0.32: 0.26), Exercise vs.	High

				number of trials and participants involved in these comparisons is limited.	pharmacological treatment: -0.11 (-0.34: 0.12), Exercise vs. bright light therapy: -6.40 (-10.20: -2.60)	
Correia et al. 2024	Meta-Analysis, 10 studies included, AE, MT, 956 individuals with depression, mean age 40.04 years (range 18-69 years), 56% female	Synthesize and analyze the dose-effect of different physical exercise protocols in adult subjects in the treatment of depression, based on the analysis of randomized controlled trials.	Depression severity	Physical exercise (PE) can help reduce depression and be used as an adjunct to pharmacological or alternative treatments.<br>Most studies show beneficial effects of PE on depression symptoms.<br>Aerobic exercises showed more significant positive effects compared to combined exercises (resistance, aerobic, and flexibility).	Reduction of depression: Exercise vs. control: -0.76 (-1.18: -0.34), Interventions >150 minutes/week vs. Control: -1.28 (-1.83: -0.74), Mild-to-moderate intensity vs. Control: -1.04 (-1.59: -0.49), Moderate intensity vs. Control: -0.34 (-0.84: 0.17), Moderate-to-vigorous intensity vs. Control: -0.57 (-1.32: 0.18), Group sessions vs. Control: -1.23 (-1.81: -0.66), Individual sessions vs. Control: 0.04 (-0.18: 0.27), Aerobic training vs. Control: -1.41 (-2.32: -0.50), Combined training vs. Control: -0.36 (-0.74: 0.01)	Critically Low
Craft et al. 1998	Meta-Analysis, 37 studies included, AE, MT, 2158 individuals	Investigate the effect of exercise on clinical depression and depression resulting from mental illness.	Reduction of depression severity	Individuals with depression who exercised were less depressed than individuals with depression who did not exercise.	Reduction depression severity: -0.72 (SE=0.1)	Critically Low
Cramer et al. 2013	Meta-Analysis, 12 studies included, MBE, 619 individuals (452 individuals in meta-analysis) with a mean age range of 21.5-66.6 years (median 33.7 years), and 36.7-100% female in included studies (median 76.5%)	Assess the effectiveness and safety of different yoga forms in patients with depressive disorders and individuals with elevated levels of depression.	Severity of depression and remission rates	The systematic review found with 12 studies limited-to-moderate evidence for short-term improvements in the severity of depression and anxiety with yoga. Despite methodological drawbacks, yoga could be considered as an ancillary treatment option for patients with depressive disorders and elevated levels of depression.	Reduction depression severity: Yoga vs. TAU: -0.69 (-0.99: -0.3),yoga vs. relaxation: -0.6 (-1.0: -0.2), yoga vs. AE: -0.59 (-0.99, -0.18)	Moderate

Cramer et al. 2017	No Meta-Analysis, 8 studies included, MBE, 240 individuals with 28.3-100% female (median 72%) in included studies with a mean age range of 28.4-43.4 years (median 38.7 years)	Investigate the efficacy and safety of yoga interventions in treating patients with MDD.	Severity of depression using clinician-assessed instruments such as the Hamilton Rating Scale for Depression, or the Quick Inventory of Depressive Symptoms-Clinician Rating, or patient-reported measures such as the Beck Depression Inventory, the Edinburgh Postnatal Depression Scale, or the Patient Health Questionnaire or specific threshold or reduction depression severity more than 50%	Some evidence supports positive effects of yoga beyond placebo and similar effects to evidence-based interventions.<br>Methodological problems and unclear risk-benefit ratio prevent definitive recommendations for or against yoga as adjunct treatment for major depressive disorder.	N/A	Low
da Cunha et al. 2023	Meta-Analysis, 12 studies included, AE, MT, 757 individuals with depression, 508 intervention groups (68% female), 249 control groups (62% female), mean age IV 44 ± 9.9 years, mean age CG 41.6 ± 12.7 years	Systematically review the chronic effect of exercise on blood biomarkers and its association with changes in depressive symptoms in adults with major depressive disorder.	Depressive symptoms	Exercise has an antidepressant effect.<br>Exercise may trigger antidepressant<br>Exercise particularly enhances levels of depression-related biomarkers, such as BDNF and kynurenine.<br>The improvement in these biomarkers is linked to a reduction in depressive symptoms in adults with MDD.	Reduction depressive symptoms: Exercise vs. control: -0.72 (-1.08: -0.37)	Moderate

Danielsson et al. 2013	Meta-Analysis, 14 studies included, AE, 1139 individuals with a range of 23-202 individuals per study, range of age 18-93 years	Examine the quality of evidence for exercise in the treatment of MDD, comparing specific study types; aerobic exercise vs. antidepressants, aerobic exercise vs. any physical activity and aerobic exercise as augmentation therapy to treatment as usual vs. treatment as usual.	Reduction of depression severity with different assessments (Hamilton Rating Scale for Depression (HAM-D), Beck's Depression Inventory (BDI), Symptom Check List-90, Montgomery Asberg Depression Rating Scale)	In general, exercise appears to be beneficial in the treatment of depression when used in combination with medication.	Reduction depression severity: AE + TAU vs. TAU: −0.44 (−0.79: −0.09), AE vs. Antidepressants: −0.06 (−0.36: 0.23), Long-term outcome AE vs. any physical activity: −0.19 (−0.68: 0.31)	Low
de Souza Moura et al. 2015	No Meta-Analysis, 13 studies included, AE, RT, NT, MBE, MT, 1570 individuals (74% women) with a mean age range of 19-57 years	Elaborate the therapeutic effects of chronic aerobic exercise compared with other types of interventions to treat depression.	Depression symptoms and cognitive functions	69.3% of studies found significant antidepressant effects of exercise on depression.<br>The remaining 30.7% of studies showed improvements in physiological aspects but not in depression symptoms.	N/A	Critically Low
Eriksson et al. 2011	No Meta-Analysis, 8 studies included, AE, RT, MT, 1494 individuals with a range of age of 18-91 years	Summarize studies which used physical exercise as an intervention to treat major depression.	Depression measured by the perseverance of a clinical diagnosis assessed with a clinical interview, change in any of the recognized depression-scales such as Hamilton rating scale of depression (HRSD), Beck depression inventory (BDI)	Physical exercise can be an effective treatment against depression. A mood enhancing effect of exercise was identified in the interventions regardless of the mechanism of action.	N/A	Critically Low

Feng et al. 2024	Meta-Analysis, 25 studies included, MB, 1605 individuals with depression, age range of 21 - 82.7 years	Evaluate the therapeutic effect of traditional Chinese exercise on depression using network meta-analysis.		The network meta-analysis showed no significant difference between various exercise methods and conventional treatments or no intervention. Baduanjin had a better intervention effect than Tai Chi for depression. Baduanjin exercise may be recommended as a clinical intervention for depression.	Depression severity: HAMD-scores: Baduanjin + TAU vs. TAU: 0.1 (-0.47: 0.67), Wuqinxi + TAU vs. TAU: -0.0 (-1.33: 1.33), Thai Chi + TAU vs. TAU: -0.05 (-0.88: 0.79), BDI-scores: Baduanjin + TAU vs. TAU: 0.28 (-0.53: 1.08), Wuqinxi + TAU vs. TAU: -0.0 (-2.59: 2.59), Self-rating depression scale: Baduanjin + TAU vs. TAU: -0.0 (-0.76: 0.76), Wuqinxi + TAU vs. TAU: -0.0 (-1.56: 1.56), Thai Chi + TAU vs. TAU: 0.02 (-0.54: 0.58)	Low
Frazer et al. 2011	No Meta-Analysis, 4 studies included, AE, RT	Elaborate the effectiveness of a range of possible treatments like exercise for depression in older people.	Effectiveness of the treatment for late life depression	There is evidence that various types of physical exercise improve mood and reduce depression in older people.	N/A	Critically Low
Gartlehner et al. 2023	Meta-Analysis, 2 studies included, AE, 201 participants	Compare benefits and harms of no pharmacologic treatments with second-generation antidepressants as first-step interventions for acute MDD, and to compare second-step treatment strategies for patients who did not achieve remission after an initial attempt with antidepressants.	Remission rate depression	Network meta-analyses showed no difference in remission rates between treatment groups. Adding exercise to antidepressants did not provide more benefits than antidepressant monotherapy. Discontinuation due to adverse events was lower for participants on aerobic exercise compared to those on antidepressants.	Risk ratio remission: Supervised aerobic exercise vs. SGA: 0.97 (0.69: 1.35), Supervised aerobic exercise + SGA vs. SGA: 1.23 (0.89: 1.70)	High

Guo et al. 2019	Meta-Analysis, 7 studies included, MBE, 382 individuals, mean age range from 38 (±9.7) - 72.6 (±4.2) years	Evaluate the efficacy of Qigong-based therapy among patients who are clinically diagnosed with MDD.	Depression severity measured by different scales (HAM-D17, HAM-D24, BDI, Epidemiological Studies Depression Scale)	Qigong-based exercises may be effective for alleviating depression symptoms in individuals with major depressive disorder.	Reduction depression: Qigong vs. active control groups: -0.47 (-0.81: -0.12), Qigong vs. passive control groups: -0.80 (-1.23: -0.37)	Critically Low
Guo et al. 2020	Meta-Analysis, 14 studies included, AE, MBE, 2010 undergraduate students	Review and compare the efficacy of 7 exercise interventions (Badminton, Basketball, Dance, Volleyball, Run, Tai chi and Yoga) for decrease symptoms of depression in college students.	Mean change of depressive symptoms assessed by different validated scales	Tai chi was the most effective intervention among the comparisons in alleviating depressive symptoms. Yoga ranked second in effectiveness for alleviating depressive symptoms in depressed college students. Dance ranked third, followed by running, volleyball, basketball, and badminton, respectively.	Reduction mean change depression symptoms: Exercise vs. TAU: -1.13 (-1.48: -0.78)	Critically Low
Heinzel et al. 2015	Meta-Analysis, 18 studies included, AE, RT, MBE, MT, DT, 1019 individuals with a range of age from 61 (±7.9) - 82.4 (±7) years	Evaluate the effectiveness of exercise on depressive symptoms among older adults.	Depression score	Physical exercise may be a feasible additional intervention for depression in older adults. This is especially relevant as many older adults with depression do not receive adequate treatment in usual care. Different types of exercise showed significant effects in subsequent subanalyses.	Reduction depression score: Exercise vs. control: -0.68 (-0.93: -0.43), Exercise vs. control (studies low risk of bias): -0.63 (-0.91: -0.35), AE vs. control: –0.64 (–1.06: –0.22), RT vs. control: –0.76 (–1.43: –0.09), alternative exercise vs. control: –0.97 (–1.58: –0.36), AE + RT vs. control: –0.28 (–0.50: –0.05), supervised exercise vs. control: –0.77 (–1.03: –0.52), homebased training vs. control: –0.14 (–0.43: 0.15), exercise vs. waiting list control: –0.70 (–1.10: –0.31), exercise vs. active control: –0.66 (–0.98: –0.35)	Critically Low
Heissel et al. 2023	Meta-Analysis, 41 studies included, AE, 2544 individuals, 2264 individuals in meta-	Update the current evidence on the effects of exercise in reducing depressive symptoms in	Mean change in depressive symptoms	Exercise interventions had a significant large effect favoring exercise over control conditions. This positive effect remained regardless of factors like bias risk, depression classification, exercise type,	Exercise vs. control: All studies: −0.946 (−1.18: −0.71), without high risk of bias: −0.717 (−1.01: −0.43), studies with more	High

	analytical calculations, female range in studies from 26-100% and mean age in studies from 18.8-87.9 years	adults with clinically elevated levels of depression including MDD and dysthymia, comparing exercise with non-exercising control groups.		group setting, supervision, or sample size. Subgroup analyses with health education, light exercise interventions, or unsupervised training showed comparable effects, though not statistically significant due to limited study numbers. Mixed aerobic and resistance training had smaller effects compared to aerobic or resistance training alone.	than 6 weeks intervention: −0.959 (−1.21: −0.71)	
Holvast et al. 2017	Meta-Analysis, 5 studies included, no exercise description, 119 individuals with a range of age 71-76.5 years with a range of 52.6 - 62.5% female	Present an overview of the effectiveness of non-pharmacological treatments, like exercise, in primary care for late-life depression.	Depression severity	One of three studies demonstrate an effect of exercise on depressive symptoms. However, although two other RCTs did find that exercise had an effect compared with inactivity, no difference was observed between the exercise group and the active controls in each study.	N/A	Low
Hooper et al. 2021	Meta-Analysis, 36 studies included, AE, RT, MT 1665 individuals, 41.7 % older adults (56 years and older), 30.7 % middle-aged adults (36-55 years), 27.8 % younger adults (18-35 years), 73.6 % female	Conducted a network meta-analysis to compare the efficacy of exercise training (EX), behavioral activation therapy (BA), cognitive-behavioral therapy (CBT), and non-directive supportive therapy (NDST).	Depression severity with different assessment tools (HAMD, BDI, BDI-II, EPDS, PHQ-9, CES-D, DASS-21, GRID, MADRS)	Findings cautiously support exercise training for adult depression treatment. Exercise training could be a useful option with effects comparable to "gold standard" therapies. However, there's insufficient evidence to recommend it as a proven therapy for clinical depression in adults. Among the four interventions, behavioral activation therapy showed likely higher efficacy, followed by cognitive-behavioral therapy, exercise training, and non-directive supportive therapy.	Depression severity: Ex vs. TAU: −0.73 (−1.02: −0.45), Ex vs. WL: -1.04 (-1.35: -0.73), Ex vs. CBT: 0.12 (-0.17: 0.42), EX vs. BA: 0.22 (-0.24: 0.68), EX vs NDST: -0.24 (-0.73: 0.25)	Critically Low
Josefsson et al. 2012	Meta-Analysis, 12 studies included, AE, RT, 720 individuals with a age range of 18-69 years	Present the results of exercise interventions in clinical depression.	Depression severity with assessed with clinical interviews (e.g., DSM-IV or International Classification of Diseases, Tenth	The main result showed a significant large overall effect favoring exercise intervention. However, when considering only studies with high methodological quality, the effect size reduced to a moderate level. Exercise may be recommended for individuals with	Reduction depression severity: Exercise vs. control: -0.77 (-1.14: -0.41), Exercise vs. control (high quality studies): -0.43 (-1.06: 0.21)	Critically Low

			Revision) or by a cut-off point on a self-report depression scale (e.g., Beck Depression Inventory, HRDS).	mild and moderate depression who are willing, motivated, and physically healthy enough to participate in a program.		
Karkou et al. 2019	Meta-Analysis, 8 studies included, DT, 351 individuals	Explore evidence of effectiveness of dance movement therapy (DMT) with people with a diagnosis of depression.	Levels of depression with self-rated standardized measurements (e.g., the Beck Depression Inventory, the Symptom Check List-90-Revision) and observational tools (e.g., the Hamilton Rating Scale for Depression)	DMT is an effective intervention for adults with depression based on studies of moderate to high quality.<br>The compilation of diverse study designs with varying quality provided a comprehensive understanding of trends in using DMT for depression treatment.	Effect DMT of levels of depression: DMT Pre vs. DMT Post: 1.10 (0.40: 1.80), DMT Pre vs. 3 months follow up: 0.69 (0.37: 1.02), Reduction levels of depression: DMT + TAU vs. Control-TAU or no care: -0.64 (-1.10: -0.18)	Low
Krogh et al. 2017	Meta-Analysis, 35 studies included, AE, RT, MT, 2498 individuals, mean age range of 21.1 (±12) - 75 (±6) years and a range of 35-100% female	Investigate the beneficial and harmful effects of exercise, in terms of severity of depression.	Depressive symptoms measured on a continuous scale assessed at the end of the intervention	Overall, exercise showed an antidepressant effect.<br>Trials with low bias risk showed no antidepressant effects of exercise.<br>Exercise did not have significant effects on quality of life, depression severity, or lack of remission during follow-up.<br>Limited data on serious adverse events and adverse events prevented drawing conclusions for these outcomes.	Reduction depression symptoms: Exercise vs. control: overall: -0.66 (-0.86: -0.46), studies with less risk of bias -0.11 (-0.41: 0.18), studies with high risk of bias -0.75 (-0.98: -0.52)	Moderate
Kvam et al. 2016	Meta-Analysis, 23 studies included, AE, RT, MT, 977 individuals with mean age range of 18-71.3 years	Evaluate the reduction in depressive symptoms or remission by exercise.	Symptoms of depression assessed with a validated scale on depression severity or indicated by remission rate	Physical exercise is an effective intervention for depression. It also could be a viable adjunct treatment in combination with antidepressants.	Reduction depressive symptoms: Exercise vs. control: -0.68 (-0.92: -0.44), exercise vs. control (without blinded outcome): -0.91(-1.22: -0.61), exercise vs. control (blinded outcome): -0.40 (-0.70: -0.11), exercise vs. control (no intention-to-treat analysis): -0.80 (-1.15: -0.46), exercise vs.	Low

					control (intention-to-treat analysis): -0.56 (-0.87: -0.25), exercise vs. control follow-up: -0.22 (-0.53: 0.09), exercise vs. no intervention: -1.24 (-1.83: -0.65), exercise vs. usual care: -0.48 (-0.80: -0.16), exercise vs. psychological treatment: -0.22 (-0.65: 0.21), exercise vs. antidepressant: -0.08 (-0.33: 0.18), exercise + antidepressant vs. medication only: -0.50 (-1.10: 0.11), exercise vs. control (allocation concealment, intention-to-treat, blinded outcome): -0.26 (-0.61: 0.08)	
Lawlor et al. 2001	Meta-Analysis, 14 studies included, AE, RT, 851 individuals with mean age range of 28.5-72.5 years and a range of 53.4-100% female	Determine the effectiveness of exercise as an intervention in the management of depression.	Standardized mean difference in effect size and weighted mean difference in Beck depression inventory score between exercise and no treatment and between exercise and cognitive therapy.	Most studies of the effect of exercise on depression are of poor quality, have brief follow up, and are undertaken on nonclinical volunteers. Exercise may be efficacious in reducing symptoms of depression in the short term but its effectiveness in clinical populations is unknown. A well designed, randomized controlled trial with long term follow up is needed	Reduction depression symptoms: Exercise vs. control (no treatment): -1.1 (-1.5: -0.6), weighted mean difference in Beck depression inventory (BDI): -7.3 (-10.0: -4.6), exercise vs. cognitive therapy: -0.3 (-0.7:0.1)	Critically Low
Lee et al. 2021	Meta-Analysis, 22 studies included, AE, RT, MT, 1025 individuals, 538 TAU, 487 Exercise + TAU, mean age of 48.5 ± 12.6 years, 65% female	Update and extend the investigation about the antidepressant effects of exercise augmentation across standard treatment modalities.	Depressive symptomology as a continuous measure at post-intervention	Adding exercise to standard treatments significantly enhances antidepressant effects compared to standard treatment alone.<br>Combining exercise with 'treatment as usual' has significant medium antidepressant effects over 'treatment as usual' alone.<br>Exercise augmentation remains effective even with multiple standard treatments.<br>Exercise plus psychotherapy shows moderate	Reduction depressive symptoms Ex + TAU vs. TAU: Overall studies: -0.62 (-0.86: -0.37), Adults <60: -0.60 (-0.87: -0.32), Older adults (60+): -0.72 (-1.33: -0.11), Pharmacotherapy: -0.68 (-1.00: -0.35), Psychotherapy: -0.49 (-1.23: 0.25), Treatment as Usual: -0.60 (-0.97: -0.23), AT; -0.59 (-0.90: -0.28), RT or MT: -0.67 (-1.09: -0.25)	Moderate

				antidepressant effects over psychotherapy alone, but the difference isn't statistically significant.		
Martinez-Calderon et al. 2024	Meta-Analysis, 20 studies, 14 studies with yoga intervention, 2 studies Qigong intervention, 4 studies tai chi intervention, MB, 1317 participants (75% female), range of mean age 26.6 - 80.2 years	Assess the effectiveness of qigong, tai chi and yoga-based modulating anxiety and depression symptoms in adults diagnosed with anxiety or depressive disorders.	Depression severity	Yoga-based interventions may reduce depressive symptoms in depressive disorders.<br>No definitive clinical recommendations can be made due to: Very low certainty of evidence, methodological concerns, heterogeneity in Qigong, Tai Chi, and Yoga styles across studies<br>A specific yoga style cannot be recommended with current evidence.<br>Improved research quality in this field is needed.	Reduction depression severity: Yoga vs. control: -0.35 (-0.73: 0.03), Qigong vs. control: -0.49(-1.59: 0.61), Thai Chi vs. control: -0.6 (-1.67: 0.47)	Moderate
Martland et al. 2020	Meta-Analysis, 3 studies included, AE, ANE, 116 individuals with mean age range of 35-38.1 years and range of 70-83% female	Establish the benefits of HIIT interventions across all categories of mental illness included depression.	Depression severity	HIIT may improve physical and mental health outcomes in individuals with mental illnesses.<br>HIIT has shown to reduce depression severity specifically in patients with MDD.	Reduction depression symptoms: HIIT Pre vs. HIIT Post: −1.36 (-1.63: -1.09), HIIT vs. MICT: -0.45 (-0.85: -0.04)	Low
Martland et al. 2023	Meta-Analysis, 27 studies included, AE, ANE, 892 inpatients, 562 intervention, 330 controls, range of mean age: 29.1 ± 5.1 - 68.7 ± 3.1 years	Investigate the benefits, safety, adherence and description of exercise interventions for individuals receiving inpatient mental health treatment like depression.	Depression severity	In hospitalized MDD patients, most trials (6/7, 85.7%) saw reduced depressive symptoms with exercise vs. non-active control.<br>In 3/9 studies (33.3%) significant depressive symptom reduction were found with exercise vs. active control, with all trials (9/9, 100%) showing significant reduction in depressive symptoms. Exercise regimes are safe, viable, and effective in inpatient mental health settings.	Reduction depression exercise vs. non-exercise: −0.42 (−0.79: −0.05)	Moderate
Md Zemberi	No Meta-Analysis, 13 studies included, AE, ANE, RT, MT, 1049	Explore the efficacy of exercise interventions as the primary	Depressive symptoms confirmed by screening with validated	AE and high-intensity RT show promise as primary treatments for depression.<br>These forms of exercise have the potential to induce	N/A	Critically Low

et al. 2020	individuals with age range of 18 - >60 years, male to female ratios ranged from 1:1–1:3 in 11 studies and in two studies from 2:1-3:1	treatment for depressive disorders.	diagnostic and rating tools for depressive disorders	biopsychosocial effects that can improve the severity of depressive symptoms.		
Mead 2008	Meta-Analysis, 23 studies included, AE, RT, MT, 1125 individuals, mean age range of 22-82.7 years, more women than men in the studies	Determine the effectiveness of exercise in the treatment of depression.	Depression or mood scale, either as a continuous measure or as a dichotomous outcome	Exercise improves depressive symptoms in people diagnosed with depression.<br>However, when considering only methodologically robust trials, the effect sizes are moderate and not statistically significant.	Reduction depression symptoms: exercise vs. no treatment or control: -0.82 (-1.12: -0.51), exercise vs. no treatment or control (adequate allocation concealment, intention to treat analysis and blinded outcome assessment): -0.42 (-0.88: 0.03)	High
Miao et al. 2023	Meta-Analysis, 9 studies, MB, 581 individuals with depression (78% female), range of age 18 -72 years	Determine the effectiveness of mindfulness yoga intervention on major depressive disorder (MDD) patients.	Depression severity	Mindfulness yoga shows promise as a non-pharmacological intervention for MDD patients.<br>Current studies are limited in number and have varied intervention methods.<br>More rigorous intervention designs, larger sample sizes, and high-quality studies are needed to confirm the efficacy and validate findings.	Reduction depression severity: Mindfulness yoga vs. Control: -0.53 (-0.96: -0.11)	Moderate
Millman et al. 2021	No Meta-Analysis, 5 studies included, DMT, 158 individuals, range of mean age: 16-48.4 years	Review the scope and effectiveness of recent research on DMT as an intervention for psychiatric disorders like depression.	Depression severity	Studies suggest that DMT interventions were associated with reduced depressive symptoms with moderate effects.	N/A	Critically Low
Morres et al. 2018	Meta-Analysis, 11 studies included, AE, 455 individuals with mean age range of	Examine the antidepressant effects of AE vs. non exercise comparators exclusively	Reduction depression severity	Supervised AE outperformed various depression treatments regardless of delivery format, comparisons, or settings, and regardless of symptom severity or outcome measure type.	Reduction depression symptoms: AE vs. non-exercise control: -0.79 (-1.00: -0.57), AE vs. antidepressants/TAU: -0.75 (-1.01: -0.48), AE vs. psychological treatment	Low

	20.9 (±1.0) - 49.1 (±16.5) years with range of 30-100% female	for depressed adults (18-65 years) recruited through mental health services with a referral or clinical diagnosis of major depression.		The antidepressant effect of AE remained consistent across trials with lower risk of bias, short-term interventions (up to four weeks), and interventions based on individual exercise preferences.<br>Despite the limited number of trials reviewed, AE was found to be an effective intervention for depression.	(mono-therapy/part of multi-therapeutic program): -0.85 (-1.21: -0.48)	
Mura, Moro et al. 2014	No Meta-Analysis, 22 studies included, AE, ANE, 1131 indiviuals with a range of 20-424 individuals in the studies	Evaluate the effectiveness of exercise as an adjunctive treatment with antidepressants on depression.	Depression severity	The included studies showed a strong effectiveness of exercise combined with antidepressants.	N/A	Low
Mura, Sancassiani et al. 2014	No Meta-Analysis, 14 studies included, AE, ANE, RT, 2072 individuals	Establish new findings on the effectiveness of exercise on depression.	Depression severity	Limited progress has been made in demonstrating the efficacy of exercise on depression in the past 12 years.<br>Challenges include a persistent lack of high-quality research and difficulties in establishing the true effectiveness of physical activity for managing depressive symptoms.<br>Both qualitative and quantitative perspectives contribute to the complexity of assessing exercise's impact on depression.	N/A	Low
Nanthakumar 2020	No Meta-Analysis, 8 studies included, MBE, 517 individuals wwith a range of 18-122 individuals in the studies and age range of 18-72 years with range of 68-100% female	Investigate the effectiveness of classical yoga not only as a complementary therapy but also as a viable option in the management of anxiety and depression.	Depression severity assessed by different instruments	Findings indicate that yoga, both as a complementary and stand-alone therapy, is effective in managing and reducing depression.	N/A	Critically Low

Nebiker et al. 2018	Meta-Analysis, 27 studies included, AE, NT, 1452 individuals (286 NT, 508 AE, 658 control) with a mean of 53.8 individuals per study	Update the effects of physical exercise in the treatment of depression and examine the effects of endurance exercise interventions and neuromuscular exercise interventions on depression.	Mean change in depressive symptoms from baseline to post-intervention	Strong NT interventions can be slightly more effective than AE interventions. Interestingly, exercise duration and exercise intensity moderated the effect size meaningfully.	Reduction depressive symptoms: AE vs. control: -0.79 (90%CI:-1.10: -0.48), NT vs. control: -1.14 (90%CI: -1.50: -0.78)	Low
Pilkington et al. 2005	No Meta-Analysis, 5 studies included, MBE, 198 individuals	Review the effectiveness of yoga for the treatment of depression.	Depression rating scales	Initial indications suggest potential beneficial effects of yoga interventions on depressive disorders. Caution should be exercised when interpreting the findings due to variations in interventions, severity, and reporting of trial methodology.	N/A	Low
Recchia et al. 2022	Meta-Analysis, 25 studies included, 2552 individuals, range of mean age: 21 ±1 - >65	Assess the comparative effectiveness of exercise, antidepressants and their combination for alleviating depressive symptoms in adults with non-severe depression.	Treatments effectiveness measured with depression scales MADRS, BDI, HAM-D	All treatments had comparable positive effects on depressive symptoms compared to controls. No treatment demonstrated superiority over another. Antidepressant treatments had fewer dropouts than exercise in terms of acceptability. Exercise interventions can be considered an alternative for non-severe depression treatment.	Treatment effectiveness: exercise vs control:−0.45 (−0.67: −0.23), exercise + antidepressants vs. control: −0.45 (−0.76: −0.14), exercise vs. antidepressants: −0.12 (−0.33: 0.10), combination vs. exercise: 0.00 (−0.33: 0.33), combination vs. antidepressants: −0.12 (−0.40: 0.16)	Moderate
Rethorst et al. 2009	Meta-Analysis, 58 studies included, AE, RT, MT, 2982 individuals with mean age ranges of 9.00-73.00 years (>75% had >18 years of age)	Determine the effect that aerobic and resistance exercise programs significantly alleviate depressive symptoms.	Depression severity	The results provide Level 1, Grade A evidence supporting the use of exercise for alleviating depressive symptoms. A very large effect size was observed within the clinically depressed population of over 500 participants, with most studies showing clinically significant improvement.	Reduction in depression symptoms: Exercise vs. control: -0.80 (-0.92: -0.67), exercise vs. control (clinically depressed individuals) : -1.03 (-1.21: -0.85), exercise vs. control (non-clinical depressed individuals): -0.59 (-0.67: -0.50)	Critically Low
Rhyner et al. 2015	Meta-Analysis, 15 studies included, AE,	Provide a quantitative review on the effect of exercise interventions	Depression severity	These findings suggest that exercise is an effective treatment option for older individuals with depressive symptoms	Reduction depression severity: Exercise vs. control: -0.97 (-1.38: -0.56)	Critically Low

	RT, mean age range of 66-88 years	on depressive symptoms in older adults.				
Robertson et al. 2012	Meta-Analysis, 8 studies included, AE, 341 individuals with a range 11-127 individuals per study and different age groups (20-70 years, 50+, 60+ and elderly with mean age 72.5 years	Examine the effectiveness of walking as an intervention for alleviating depression in adults.	Depression severity assessed by several outcome measures (Edinburgh Postnatal Depression Scale, Becks Depression Inventory, Becks Depression Inventory-ll, the Geriatric Depression Scale, the Hamilton Rating Scale for Depression and Bech-Rafaelsen Melancholy Scale)	Walking has a statistically significant, large effect on depression symptoms in certain populations. However, the current evidence base from randomized, controlled trials is limited.	Reduction depression symptoms: AE vs. control: -0.86 (- 1.12: -0.61), AE vs. control (high quality studies): -0.69 (-0.99: -0.39)	Critically Low
Rossi et al. 2024	Meta-Analysis, 9 studies, RT, MT, 397 individuals with depression, range of age 18 - 81 years	Summarize and estimate the efficacy of strength training in people with a diagnosis of depression or subthreshold depressive symptoms and no other severe disease.	Depression severity	Strength training (ST) in individuals with depression has a high antidepressant effect based on 9 studies. ST should be considered as an adjunct strategy for treating depression or depressive symptoms.	Reduction depression severity: RT vs. control: -0.82 (-1.34: -0.3)	Moderate
Sani et al. 2023	Meta-Analysis, 8 studies included, MBE, 822 individuals, 407 intervention, 415 control, range of mean age: 18.4 ± 2 - 72.6 ± 4.2 years	Determine the effects of Tai Chi exercise on depressive symptoms in individuals with depression.	Depression level	Tai Chi intervention reducing depression in patients with depressive symptoms.<br>Tai Chi exercise reduced depression in patients receiving antidepressant therapy and in milder depression forms without active interventions like antidepressant therapy or mindfulness-body exercises.	Reduction depression symptoms: Tai Chi + TAU vs. TAU: -0.58 (-1.13: -0.03), Tai Chi vs. control: -1.32 (-2.11: -0.52)	Moderate

Schuch, Vancampfort, Richards et al. 2016	Meta-Analysis, 9 studies included, AE, RT, MT, 1487 individuals with mean age range of 18.4-76.4 years, range of 17-100% female	Establish the updated effects of exercise on depression comparing exercise versus non-active control groups.	Mean change in depressive symptoms assessed by any validated scale	Exercise has a large and significant antidepressant effect in individuals with MDD.	Reduction depressive symptoms: Exercise vs. control: -1.14 (-1.81: -0.46)	Low
Schuch, Vancampfort, Rosenbaum, et al. 2016	Meta-Analysis, 2 studies included, AE, RT, MT, 267 individuals with mean age of 69.5 (±0.7) years for exercise and 70.5 (±2.1) years for control groups	Evaluate the antidepressant effects of exercise in older adults.	Depressive symptoms using a validated measure (e.g., HAM-D, BDI, and GDS)	Adjusting for publication bias reveals increased beneficial effects of exercise in three subgroup analyses. In clinically depressed older adults there is no beneficial effect of exercise in depression treatment vs. control condition.<br>Previous meta-analyses may have underestimated exercise benefits due to publication bias.<br>Exercise should be considered as a routine component of depression management in older adults.	Reduction depression symptoms: Exercise vs. control (individuals with MDD): -1.88 (-4.21: 0.44)	Low
Seshadri et al. 2021	Meta-Analysis, 25 studies included, AE, MBE, 2083 individuals with mean age range of 30-75 years, range of 40-100% female	Synthesize the evidence for exercise, yoga, and tai chi in the outpatient treatment of MDD.	Change in depressive symptom severity assessed using standardized rating scales	Meta-analysis suggests that adjunctive exercise and yoga have small additive clinical effects in reducing depressive symptoms for major depressive disorder.<br>Insufficient evidence exists to draw conclusions about the effectiveness of tai chi.	Reduction depression severity: Exercise, yoga, tai chi vs. Control: -0.63 (-0.76: -0.50), exercise, yoga vs. control (studies with low risk of bias): -0.41 (-0.66: -0.14)	Low
Silveira et al. 2013	Meta-Analysis, 10 studies included, AE, RT, 1759 individuals with mean age range of 18-71.6 years	Evaluate the effect of aerobic training and strength training as a treatment for depression in patients diagnosed with MDD.	Depression severity	Despite study heterogeneity, the meta-analysis found that physical exercise improves treatment response, particularly aerobic training.<br>Age and symptom severity were factors influencing the efficacy of exercise in treating depression.	Reduction depression severity: AE or RT vs. control: -0.61 (-0.88: -0.33), AE vs. control: -0.52 (-0.79: -0.25), RT vs. control: -0.96 (-1.97: 0.05)	Critically Low
Sjosten et al. 2006	No Meta-Analysis, 5 studies included, AE, MBE, 318 individuals	Determine the effects of physical exercise on depression or	Depression assessed by the Diagnostic and Statistical Manual of	Physical exercise may efficiently reduce clinical depression and depressive symptoms in the short-term.	N/A	Critically Low

	with mean age range of 57-72.6 years	depressive symptoms among the aged.	Mental Disorders (DSM) or the International Classification of Diseases (ICD-10) criteria or as a self-rated condition measured by depressive symptoms	This is especially true for older individuals who are experiencing depression or a high level of depressive symptoms.		
Stanton et al. 2014	No Meta-Analysis, 4 studies included, AE, ANE, MT, 117 individuals with age range of 18-62 years	Examining the effect of exercise interventions on the health of people hospitalized with depression.	Depression severity assessed by validated rating scales	Exercise programs delivered in an inpatient setting are effective in improving mental health outcomes for people with various mental illnesses.<br>Studies with inpatient populations show that exercise is a safe and well-tolerated intervention for conditions including depressive disorders.	N/A	Critically Low
Stathopoulou et al. 2006	Meta-Analysis, 11 studies included, AE, ANE, RT, 513 individuals	Update the substantial benefit from exercise in populations diagnosed depression.	Remission rate, response rate and depressive symptom severity	Findings support the role of adjunctive exercise interventions in clinical practice.<br>Clinicians are encouraged to consider integrating exercise interventions into their practice.<br>Issues related to this integration are discussed.	Advantage depression treatment: Exercise vs. Control: 1.39 (0.89: 1.88)	Critically Low
Sukhato et al. 2016	Meta-Analysis, 5 studies included, AE, RT, MT, 490 Individuals (3 studies exercise vs. usual care: n=321, 2 studies psychological intervention + exercise vs. usual care: n=169), age range of 43.9-81.45 years, range of 30.6-	Compare the efficacy of all available home-based non-pharmacological treatments of depression.	Depression symptom scores and disease remission rates at the end of treatment	Of all the studied interventions, combined homebased psychological intervention with exercise intervention had the highest probability of resulting in disease remission.	Reduction depression severity: Home-based exercise vs. Usual care: -1.03 (-2.89: 0.82), Combined psychological intervention with exercise vs. usual care: -0.78 (-1.09: -0.47)	Low

	91.6% female in all included studies					
Tang et al. 2024	Meta-Analysis, 47 studies included, AE, RT, MB, MT, 2895 participants, mean age ranged from 59.6 - 87.9 years (median 72.6 years)	Examine the dose–response relationship between overall and specific types of exercise with depression symptoms in older adults.	Depressive symptoms	Walking, aerobic exercise (AE), yoga, Qigong, resistance training (RT), and Tai Chi (TC) effectively reduce depressive symptoms in older adults.<br>Statistically and clinically significant threshold doses were established for each exercise type.<br>Starting exercise early is beneficial, but its efficacy declines after age 80 and becomes insignificant beyond age 81.	Reduction depression severity: AE vs. CG: -0.78 (-1.1: -0.46), MT vs. CG: -0.26 (-0.56: 0.03), Qigong vs. CG: -0.68 (-1.04: -0.32), RT vs. CG: -0.68 (-0.98: -0.38), Thai Chi vs. CG: -0.66 (-1.14: -0.19), Walking vs. CG: -0.87 (-1.24: -0.51), Yoga vs. CG: -0.72 (-1.11: -0.32)	Moderate
Tian et al. 2024	Meta-Analysis, 46 studies, AE, RT, MB, MT, 3164 individuals with depression, mean age IG: 48.7 years, CG: 51.7 years	Examined the efficacy of four major types of exercise (aerobic, resistance, mixed, and mind-body) on depression, as well as the dose-response relationship between total and specific exercise and depressive symptoms.	Depression severity	Various exercise interventions (aerobic, resistance, mixed, and mind-body) significantly improve depressive symptoms in MDD patients.<br>Patients can choose their preferred exercise type, enhancing treatment outcomes.<br>A dose-response analysis revealed: 140 min/week of aerobic exercise improves depressive symptoms, 50 min/week of mind-body exercise yields similar effects.<br>Clinicians should tailor exercise doses based on individual patient needs, alongside psychological care interventions.	Reduction in depression: AE vs. CG: -0.95 (-1.28: -0.62), MB vs. CG: -0.83 (-1.24: -0.43), MT vs. CG: -0.72 (-1.17: -0.28), RT vs. CG: -0.7 (-1.2: -0.19)	Low
Tsang et al. 2008	No Meta-Analysis, 12 studies included, AE, 591 individuals with a range of 14-100 individuals per study and a mean age range of 28.2-82.4 years	Examine the effectiveness of mindful and non-mindful physical exercises as an intervention in managing depression or depressive symptoms.	Depression severity	Mindful and no mindful physical exercises were effective in reducing depression levels or depressive symptoms in the short term.<br>Methodological problems, such as small sample sizes and lack of reporting on maintenance effects, were common in the studies.	N/A	Low
Vollbehr et al. 2018	Meta-Analysis, 18 studies included, MBE, 1532 individuals with	Investigate the effectiveness of hatha yoga in treating acute,	Symptoms of depression	Firm conclusions are limited due to the heterogeneity and low quality of most included studies.<br>The meta-analysis suggests that hatha yoga does not	Reduction in depression symptoms: Yoga vs. TAU: -0.64 (-1.41: 0.13), yoga vs. active control: -0.13 (-0.49: 0.22), yoga	Low

	a range of 12-620 individuals per study and a mean age range of 36.6 ± 9.1 years, range of 22.1-50.4 years, with 78.7% female	chronic and/or treatment-resistant mood and anxiety disorders like depression.		have significant effects on acute, chronic, and/or treatment-resistant mood and anxiety disorders compared to treatment as usual or active control groups.<br>However, when compared to psychoeducation, hatha yoga showed more reductions in depression.	vs. psychoeducation control groups: -0.52 (-0.96: -0.08), hatha yoga vs. active control groups: -0.14 (-0.60: 0.33)	
Wu et al. 2023	Meta-Analysis, 34 studies included, MBE, 2341 individuals, 1269 intervention, 1072 control, range of mean age: 26.2 ± 6.3 - 51.3 ± 9.2 years	Validate the evidence that yoga could be used as an adjunctive intervention to improve outcomes in the treatment of patients with major depressive disorder.	Depression symptoms measured by BDI-II, HAMD, CES-D	Yoga enhances depressive symptoms in MDD patients, being well-tolerated and safe.	Improvement depressive symptoms: Only yoga vs. control: -0.83 (-1.94: 0.29), yoga + medications vs. control: -0.64 (-0.98: -0.30), assessment BDI-II: −0.60 (−1.00: −0.21), assessment HAMD: −0.64 (−0.98: −0.30), assessment CES-D: −0.55 (−0.89: −0.22)	Moderate
Yang et al. 2023	Meta-Analysis, 10 studies included, MB, 2216 patients overall in 22 studies, just 10 studies with individuals with depression, age range 18 - 70 years	Systematically evaluate and apply meta-analysis to determine the effect of yoga treatment on depressed patients, in order to draw objective and accurate conclusions and provide a theoretical reference for the future application of yoga in mental health treatment and to promote the integration of somatic medicine.	Severity of depression	Mindfulness meditation combined with group yoga effectively improves depression in patients.<br>This intervention enhances the quality of life for patients with depression.<br>Mindfulness yoga is a nonmedical, low-cost intervention that can complement pharmacological treatment for depression.<br>It is considered valuable for clinical application in preventing and treating depression.	Reduction in depression: mindfulness yoga vs. Control: -1.21 (-1.71: -0.71)	Critically Low
Yu et al. 2022	Meta-Analysis, 70 studies included, AE,	Compare and rank the effectiveness of various	Depression severity	Aerobic exercise, MBE, and MT exhibited significant effects on depressive symptoms compared to	Effectiveness: AE vs. Control: 0.98 (0.46: 1.50) MBE vs. the control: 0.78 (0.46:	Moderate

	ANE, RT, MBE, MT, 2341 individuals, 4023 individuals, 2062 intervention, 1961 control, range of mean age: 15.4 ± 0.9 - 75.6 ± 5.6 years	exercise types in the treatment of depression.		controls, with moderate-to-large effect sizes (all SMDs > 0.78).<br>Different exercise methods could provide clinically meaningful benefits in treating depression, prompting clinicians to consider exercise alongside medication.<br>MT had the highest likelihood of being the optimal exercise for improving depressive symptoms among different exercise types.	1.11), MT vs. the control: 2.66 (0.96: 4.37), AE vs. RT: -0.56 (-0.92: -0.20), AE vs. stretching: 0.49 (0.00: 0.99), AE vs other types of exercise: -0.06 (-0.43: 0.31)	
Zeng et al. 2023	Meta-Analysis, 12 studies included, MB, 723 participants, age range >45 - >75 years, female 50 - 65 %	Evaluate the efficacy of Tai Chi, a mind–body movement therapy originating from China, on depression in middle-aged and older adults.	Psychological well-being	Tai Chi can be an effective alternative therapy for reducing depression in middle-aged and older adults.<br>Best therapeutic effects achieved with more than 24 weeks of Tai Chi, totaling over 2400 minutes.<br>The 24-style Tai Chi is recommended for optimal results in this population.<br>Caution advised due to potential lower-quality studies in the RCTs analyzed, which may affect the conclusions' credibility.	Reduction depression severity: Thai Chi vs. control: -1.21 (-1.59: -0.83), weeks duration < 24w: -0.77 (-1.08: -0.47), weeks duration >24w: -1.58(-2.09, -1.08), intervention period <2400min: -0.68 (-1.07:-0.28), intervention period >2400min: -1.31 (-1.71: -0.92)	Moderate
Zielinski et al. 2021	No Meta-Analysis, 3 studies included, MT (climbing), 200 individuals, 97 intervention, 103 control, range of mean age: 42.4 - 57.7 years	Evaluate studies on the impact of sport climbing as a complementary form of treatment on reducing depressive symptoms.	Depression severity	Sport climbing, especially bouldering, is effective in treating depression.<br>The impact of climbing on reducing depressive symptoms is intricate and can be explained through various mechanisms.	N/A	Critically Low
Zou et al. 2018	Meta-Analysis, 15 studies included, MBE, 844 individuals with a range of 14-122 individuals per study and a mean age range	Examine if meditative movements (Tai Chi, Qigong, and Yoga) are effective for treating MDD or not.	Depression severity	Meditative movements offer therapeutic effects for treating MDD, providing an alternative to conventional treatments.<br>Meditative movements are safe, easily accessible, and clinicians may consider recommending them for symptomatic management in this population.	Reduction in depression severity: Meditative movements vs. control: -0.56 (-0.76: -0.37), improved treatment remission rate: meditative movements vs. control (passive control): Odd ratio (OR) 6.7 (2.38: 18.68), not improved	Low

of 26.6 - 72.6 years with a range of 36.7-100% female	treatment remission rate: meditative movements vs. control (active control): Odd ratio (OR) 1.06 (0.38: 2.92)

Abbreviations: AE: Aerobic exercise, AN: Anaerobic exercise, BA: Behavioral activation therapy, BDI: Beck depression inventory, BDI-II: Beck depression inventory, second edition, CES-D: Center for epidemiologic studies depression scale, CG: Control group, DASS-21: Depression anxiety stress scales, 21-item version, DMT: Dance movement therapy, DT: Dance therapy, DSM-IV: Diagnostic and statistical manual of mental disorders, fourth edition, EPDS: Edinburgh postnatal depression scale, HAM-D: Hamilton depression rating scale, HAM-D17: Hamilton depression rating scale, 17-item version, HAM-D24: Hamilton depression rating scale, 24-item version, HIIT: High-intensity interval training, MBE: Mind-body exercise, MDD: Major depressive disorder, MADRS: Montgomery-Åsberg depression rating scale, MT: Mixed training, NDST: Non-directive supportive therapy, NT: Neuromuscular training, OR: Odds Ratio, PE: Physical exercise, PHQ-9: Patient health questionnaire-9, RR: Relative risk, RT: Resistance training, SMD: Standardized mean difference, SE: Standard error, TAU: Treatment as usual.

S6 - Supplement 6 Heart rate variability and major depressive disorders

**Reference**	**Characteristics**	**Objective**	**Outcomes**	**Conclusions**	**Effects Size**	**AMSTAR 2 Review Quality**
Brown et al. 2018	Meta-Analysis, 5 studies included, 555 depressed individuals, 348 non-depressed individuals (Clinical studies), 1526 depressed individuals, 12325 non-depressed individuals (community based studies)	Examine relationship between heart rate variability and depression in adults with a mean age over 60.	Overall HRV, high-frequency HRV, low-frequency HRV	HRV functioning is reduced in older adults with depression. Clinical depression and sub-syndrome depressive symptoms are associated with HRV reductions in older adults. LF-HRV may be particularly implicated in older adults with depression.	HRV: -0.33 (-0.58: -0.09), HF-HRV: -0.33 (-0.69: 0.02), LF-HRV: -0.62 (-1.08: -0.17)	Moderate
Cukic et al. 2023	Meta-Analysis, 26 studies included, 1537 individuals with MDD, 1041 healthy controls	Introduce cardiological screening to patients with depression and apply nonlinear methods to HRV analysis for more accurate and reliable screening results.	Overall nonlinear HRV, detrended fluctuation analysis (DFA), entropy-based measures, all other nonlinear measures	Nonlinear methods showed larger effect sizes compared to conventional time and spectral methods. This suggests the use of nonlinear methods for early screening of cardiovascular abnormalities in depression patients to prevent potential harmful events.	Overall nonlinear HRV: 1.03 (0.70: 1.35), detrended fluctuation analysis:0.36 (0.24: 0.49), entropy-based measures: 1.05 (0.57: 1.52), all other nonlinear measures: 0.70 (0.42: 0.98)	Critically Low
Kemp et al. 2010	Meta-Analysis, 18 studies included, 673 depressed individuals, 407 healthy controls	Compare HRV in individuals with MDD and healthy controls.	Time-frequency HRV, low-frequency HRV, high-Frequency HRV, LF/HF Ratio of HRV	Depression without CVD is linked to reduced HRV. HRV decreases with increasing severity of depression.	Overall HRV: -0.31 (-0.71: 0.09), time-frequency HRV: -0.29 (-0.5: -0.08),	Critically Low

				Nonlinear measures of HRV show the most pronounced association with depression severity.	LF-HRV: -0.10 (-0.40: -0.20), HF-HRV: -0.21 (-0.4: -0.02),<br>LF/HF Ratio: 0.66 (0.20: 1.13)	
Koch et al. 2019	Meta-Analysis, 21 studies included, 2250 depressed individuals and 1982 healthy controls	Compare resting-state measures of HRV between unmedicated individuals with MDD and healthy controls.	High-frequency HRV, low-frequency HRV, LF/HF ratio of HRV, Time-frequency HRV with RMSSD and SDNN	HRV measures were lower in individuals with MDD compared to healthy controls.<br>The findings support the idea of lower HRV as a potential cardiovascular risk factor in patients with MDD	HF-HRV: −0.32 (−0.39: −0.25),<br>LF-HRV: −0.2 (-0.33: −0.06),<br>LF/HF Ratio: 0.2 (0.09: 0.30),<br>RMSSD: −0.46 (−0.61: −0.31),<br>SDNN: −0.27 (−0.43: −0.10)	Critically Low
Ramesh et al. 2023	No Meta-Analysis, 6 studies included, 485 individuals with depression and non-depressed individuals (53% female), range of age 35 ± 12 - 65 ± 4.8 years	Review the current literature on heart rate variability as a diagnostic and prognostic tool in psychiatric populations.	HRV frequency domains: LF, HF, HRV time domains: RMSSD, SDNN, pNN50 and resting heart rate	HRV parameters are decreased in individuals with depression.<br>These factors can influence both psychiatric disorder progression and HRV.<br>Future studies should use consistent randomization, matching, HRV determination methods, and data analysis to control for confounders.<br>There is potential for HRV to be associated with the severity of psychiatric disorders.<br>Further research may enable HRV to enhance diagnosis and prognosis of psychiatric disorders.		Moderate

Rottenberg 2006	Meta-Analysis, 13 studies included, 312 depressed individuals, 374 non-depressed individuals	Estimate depression effects on CVC.	Overall HRV	Only in 3 out of 13 studies was an overall effect in difference of HRV between depressed individuals and non-depressed individuals.	Overall HRV: 0.33 (-0.18: 0.49)	Critically Low
Wang et al. 2023	Meta-Analysis, 19 studies included, 1078 individuals with MDD, 1121 healthy controls	Compare HRV in emotional disorders like MDD with that in healthy controls.	HRV Frequency domains: LF, HF, and the LF/HF ratio and HRV time domains (RMSSD and SDNN)	Pairwise meta-analysis revealed significantly reduced HRV in patients with MDD compared to controls.<br>Frequency- and time-based parameters were reduced in individuals with MDD, while the LF/HF ratio was increased.	HF: -0.39 (- 0.59: - 0.18),<br>RMSSD: -0.66 (-1.09: -0.24),<br>SDNN: -0.41 (-0.60: -0.23),<br>LF/HF ratio: 0.14 (0.01: 0.27),<br>LF-HRV: -0.06 (-0.36: 0.23)	Moderate
Wu et al. 2023	Meta-Analysis, 43 studies included, 5906 subjects, 2359 individuals with depression, 3547 healthy controls (overall 57.6% female), range of age: 18.8 ± 0.36 - 74.1 ± 8.1 years	Providing a theoretical basis for the diagnosis of depression and the prevention of cardiovascular diseases.	HRV frequency domains: LF, HF, LF/HF ratio, HRV time domains: RMSSD, SDNN, pNN50	HRV measures are generally lower in depressed individuals compared to healthy populations, except for LF/HF ratio.<br>This suggests that people with depression may have a higher risk of cardiovascular diseases.<br>The meta-analysis provides evidence of altered HRV in depressed patients.<br>HRV testing could be an effective tool for assessing cardiovascular health and autonomic function in depressed individuals.<br>This has important implications for	SDNN: -0.87 (-1.14: -0.6),<br>RMSSD: -0.51 (-0.69: -0.33),<br>pNN50: -0.43 (-0.59: -0.27),<br>LF-HRV: -0.34 (-0.55: -0.13),<br>HF-HRV: -0.51 (-0.69: -0.33),<br>LF/HF ratio: -0.05 (-0.27: 0.18)	Moderate

	preventing and monitoring cardiovascular disease in those with depression.	

Abbreviations: CVC: Cardiac vagal control, CVD: Cardiovascular disease, DFA: Detrended fluctuation analysis, HF: High-frequency HRV, HRV: Heart rate variability, Indiv.: Individuals, LF: Low-frequency HRV, LF/HF Ratio: Ratio of low-frequency to high-frequency HRV, MDD: Major depressive disorder, pNN50: Proportion of NN50 (intervals higher than 50ms) divided by the total number of NN (RR) intervals, RMSSD: Root mean sum of squared distances, SDNN: Standard deviation of the NN interval.

64 **S7 - Supplement 7** Physical activity and heart rate variability

Reference	Characteristics	Objective	Outcomes	Conclusions	Effect Size	AMSTAR 2 Review Quality
Bhati et al. 2018	Meta-Analysis, 13 studies included, RT, 461 individuals with range of 22-72 years, 30% female	Investigate the effects of resistance training on cardiac autonomic control.	Time-based HRV (RMSSD, SDNN, Mean NN), frequency-based HRV (LF-HRV, HF-HRV, LF/HF ratio), non-linear HRV	RT has no or only minimal effects on the cardiac autonomic control in healthy individuals.	Mean NN: -0.04 (-1.46: 0.61), SDNN: 0.43 (-0.20: 1.05), RMSSD: 0.63 (-0.09: 1.34), LF-HRV: 0.08 (-0.40: 0.57), HF-HRV: 0.01 (-0.34: 0.36), LF/HF ratio: -0.02 (-0.63: 0.59), Non-linear HRV: 1.12 (-0.06: 2.29)	Critically Low
Casanova-Lizon et al. 2022	Meta-Analysis, 26 studies included, AE, AN, RT, 649 individuals intervention group, 389 participants control group	Investigate the training-induced effect on cardiac parasympathetic nervous system (PNS) activity assessed by resting heart rate variability (HRV).	Time-based HRV (RMSSD), frequency-based HRV (HF-HRV), vagal related HRV (combination of RMSSD, HF and beat-to-beat R-R interval variability)	Exercise training improves parasympathetic nervous system (PNS) modulation in sedentary individuals.<br>Exercise performed three times a week for 30 minutes with moderate intensity is sufficient to enhance vagal-related HRV indices in this population.	RMSSD: 0.57 (0.23: 0.91), high frequency (HF): 0.21 (0.01: 0.42)	Low
Corso et al. 2022	No Meta-Analysis, 3 studies included, RT, 38 participants intervention group, 48 participants control group, range of age 57-68 years	Analyze the literature according to the chronic effects of RT on HRV in apparently healthy patients.	LF,HF, ratio LF/HF, and RMSSD	Strength training over a longer period of time showed no effects on time- and frequency-based parameters of HRV in healthy individuals.		Critically Low

Grassler et al. 2021a (young and middle age adults)	No Meta-Analysis, 8 studies included, AE, ANE, RT, NT, 843 individuals with a mean age of 44 years	Give an overview of the effects of different physical training modalities on resting HRV in young and middle-aged healthy adults.	Linear HRV with time- and frequency based parameters and non-linear HRV	Higher training intensities and frequencies are more likely to improve HRV.<br>16 out of 26 studies reported significant positive adaptations in HRV after physical interventions.<br>Time-domain parameters showed significant improvement in 10 out of 19 studies.	Critically Low
Grassler et al. 2021b (middle age adults)	No Meta-Analysis, 26 studies included, AE, ANE, RT, NT, MT, 569 participants intervention, 274 participants control, 35% women, mean age 27.4 ± 7 years ranged from 19.2-42 years	Investigate if exercise interventions, especially aerobic exercise, have a positive influence on HRV.	Time-based (RMSSD and more), frequency-based (HF and more) and non-linear HRV parameters, resting heart rate	There are positive effects of various physical training types on autonomic function in healthy young to early middle-aged adults.<br>Resting HRV parameters showed an increase following different training modalities.	Critically Low
Grässler et al. 2021c (older adults)	No Meta-Analysis, 13 studies included, AE, RT, NT, MT, 289 participants intervention, 133 participants control,71% women, mean age 67.8 ± 5.4 years,	Summarize the effects of endurance, resistance, coordinative, and multimodal exercise interventions on resting HRV in healthy older adults aged 60 years in average and over.	Frequency-based parameters (HF, LF and more), time-based parameters (RMSSD and more), non-linear parameters (SD1 and SD2) and resting heart rate	There are positive effects on cardiac autonomic control in healthy older adults through endurance, coordinative, and multimodal training (not resistance training alone).<br>Exercise modalities are equally effective in older adults as in younger adults.	Moderate

	ranged from 59.2-78.8 years					
Kingsley et al. 2016	No Meta-Analysis, 8 studies included, RT, 171 individuals with a range of 19-55 years	Examine the effect of resistance exercise both acutely and after training on HRV in healthy individuals.	Linear HRV with time- and frequency based parameters and non-linear HRV	Resistance training may not improve resting HRV in healthy young adults due to normal cardiac autonomic function.<br>Conventional resistance training does not have beneficial effects on resting HRV in individuals without evidence of autonomic modulation dysfunction at rest, regardless of age.		Critically Low
Maity et al. 2024	Meta-Analyis, 6 studies, MB, 653 participants	Evaluate the effectiveness of yoga intervention in improving cardiopulmonary parameters such as blood pressure, heart rate, pulmonary function tests and psychosomatic symptoms such as depression, anxiety and stress in medical and dental students.	Resting heart rate	Yoga enhances both physical and mental well-being in medical and dental students. It improves cardiovascular and respiratory function.<br>Effective in lowering resting heart rate.	Mean difference: Decrease resting heart rate of 2.55 beats/min (-4.36: -0.75)	Critically Low
Posadzki et al. 2015	Meta-Analysis, 2 studies included, MBE, 821 individuals with a mean age 37.3 years	Summarize the effects of yoga on HRV.	E:I Ratio (Ratio of sympathetic nervous system activity "E" and parasympathetic system activity "I"), 30:15 Ratio	Overall, the evidence from RCTs on yoga's effect on HRV modulation in healthy individuals is not convincing.	E:I Ratio: 0.63 (-0.72: 1.99), 30:15 Ratio: 0.20 (-0.43: 0.84)	Critically Low

	(range of 11.5-68.5 years, >75% >18 years)		(Ratio of parasympathetic and sympathetic nervous system activity)			
Raffin et al. 2019	Meta-Analysis, 12 studies included, AE, 218 individuals with a range of 69.0 ± 3.2 years, 144 (66.1%) female	Examine the effectiveness of exercise in terms of increasing or at least preventing the decline in HRV in older adults.	Time-based HRV (RMSSD, SDNN), frequency-based HRV (HF-HRV)	Endurance exercise training effectively increases global cardiac autonomic function in older adults. Endurance exercise influence positively time- and frequency-based HRV.	Effects all studies: RMSSD: 0.09 (-0.20; 0.36), SDNN: 0.37 (0.19; 0.55), HF: 0.09 (-0.07: 0.24), Effects all controlled studies: RMSSD: 0.3 (-0.06; 0.66), SDNN: 0.61 (-0.06; 0.13), HF: 0.24 (-0.03: 0.51)	Low
Sandercock et al. 2005	Meta-Analysis, 13 studies included, AE, 322 individuals with age ≥18 years	Determine the effects of exercise training on heart rate and measures of heart rate variability.	RR intervals, high-frequency HRV (HF-HRV)	Exercise training leads to significant increases in RR interval and HF power. The effect size for HF HRV and its relationship with RR interval vary based on the age of the study population. There are smaller responses in older adults.	RR interval: 0.75 (0.51: 0.96), HF HRV: 0.48 (0.26: 0.70)	Critically Low
Sant'Ana et al. 2020	No Meta-Analysis, 2 studies included, AE, ANE, 251 individuals with a mean range of 61.7 - 73.5 years, 144 (57%) female	Synthesize findings of the cardiovascular effects of aerobic and anaerobic interval training in healthy elderly subjects.	Resting heart rate, time- (RR interval, RMSSD, PNN50) and frequency-based (HF, LF, LF/HF ratio) HRV	Interval training improves the resting heart rate (decreasing), RR intervals (increasing) and the time-based HRV SDNN (decreasing) and RMSSD (increasing). There was no effect in PNN50 and the frequency-based HRV LF and HF.		Critically Low
Tyagi et al. 2016	No Meta-Analysis, 32 studies included, MBE, 1371 individuals included	Give an overview on the long- and short-term effects of	Time-based HRV (SDNN, RMSSD, PNN50) and frequency-based HRV (LF, HF, LF/HF ratio)	Various yoga practices can improve autonomic regulation, through an interaction of breathing, muscle contraction and heart beat regulation. Due to the		Critically Low

		different yoga practices on HRV.		heterogeneous nature of the studies, it is difficult to draw concrete conclusions.		
Yang et al. 2024	Meta-Analysis, 29 studies, AE, ANE, RT, MT, 1317 participants, 48 % female, range of age 18.2-67.32 years	Use a network meta-analysis (NMA) to compare the effects of aerobic training, resistance training, combined training, and high-intensity interval training on adult heart rate variability.	Time-based HRV (SDNN, RMSSD) and frequency-based HRV (LF, HF, LF/HF ratio)	HIIT is the most effective exercise for improving SDNN, RMSSD, and LF/HF power ratio in healthy adults.<br>Resistance training (RT) is most effective for improving HF power.<br>Circuit-based training (CBT) is best for improving LF power.<br>Future studies should explore the effects of exercise modalities on HRV across different age and gender subgroups.	RMSSD: HIIT vs. CG: 0.26 (-0.07: 0.58), AE vs. CG: 0.06 (-0.11: 0.22), RT vs. CG 0.04 (-0.2: 0.28), MT vs. CG: 0.11 (-0.18: 0.4), SDNN: HIIT vs. CG: 0.51 (0.09: 0.94), AE vs. CG: 0.01 (-0.15: 0.16), RT vs. CG 0.02 (-0.19: 0.24), MT vs. CG: 0.00 (-0.22: 0.23), HIIT vs. RT: 0.49 (0.02: 0.96), HIIT vs. AE: 0.51 (0.08:0.93), HIIT vs. MT: 0.51 (0.04: 0.98), LF: HIIT vs. CG: 0.12 (-0.27: 0.51), AE vs. CG: 0.04 (-0.13: 0.2), RT vs. CG -0.02 (-0.21: 0.18), MT vs. CG: -0.03 (-0.24: 0.18), HF: HIIT vs. CG: 0.08 (-0.21: 0.37), AE vs. CG: 0.05 (-0.1: 0.19), RT vs. CG 0.09 (-0.09: 0.27), MT vs. CG: -0.02 (-0.23: 0.18), LF/HF ratio: HIIT vs. CG: -0.52 (-0.9: -0.13), AE vs. CG: 0.11 (-0.11: 0.33), RT vs. CG	Low

0.15 (-0.16: 0.46), MT vs. CG: 0.17 (-0.21: 0.54), HIIT vs. AE: -0.63 (-1.0: -0.26), HIIT vs. RT: -0.67 (-1.14: -0.19), HIIT vs. MT: -0.68 (-1.19: -0.17)

Abbreviations: AE: Aerobic exercise, ANE: Anaerobic exercise, BA: Behavioral activation therapy, BDI: Beck depression inventory, BDI-II: Beck depression inventory, second edition, CES-D: Center for epidemiologic studies depression scale, CG: Control group, DASS-21: Depression anxiety stress scales, 21-item version, DMT: Dance movement therapy, DT: Dance therapy, E:I Ratio: Expiratory-inspiratory ratio , EPDS: Edinburgh postnatal depression scale, HAM-D: Hamilton depression rating scale, HAM-D17: Hamilton depression rating scale, 17-item version, HAM-D24: Hamilton depression rating scale, 24-item version, HF: High-frequency HRV, HIIT: High-intensity interval training, LF: Low-frequency HRV, LF/HF Ratio: Ratio of low-frequency to high-frequency HRV, MBE: Mind-body exercise, MDD: Major depressive disorder, MADRS: Montgomery-Åsberg depression rating scale, Mean NN: Mean of the NN interval, MT: Mixed training, NE: Neuromuscular training, OR (Odds Ratio): A statistical measure of the association between an exposure and an outcome, PE: Physical exercise, PHQ-9: Patient health questionnaire-9, PNN50: Proportion of NN50 (intervals higher than 50ms) divided by the total number of NN (RR) intervals, RMSSD: Root mean sum of squared distances, RR interval: Mean of the RR interval, RR: Relative risk, RT: Resistance training, SD1: SD of the instantaneous beat-to-beat variability, SDNN: Standard deviation of the NN interval, SMD: Standardized mean difference, SE: Standard error, TAU: Treatment as usual, 30:15 Ratio: Ratio of the sympathetic and parasympathetic nervous systems during orthostatic adaptation
